# Altered Mitochondrial Base Excision Repair and Mitochondrial DNA Instability in Peripheral Leukocytes of Patients with MASLD

**DOI:** 10.3390/cells15151415

**Published:** 2026-08-05

**Authors:** Sylwia Ziółkowska, Marcin Kosmalski, Bianka Świderska, Agnieszka Szczypiorowska, Kinga Jarmusz, Magdalena Ejsmont, Adam Marek Wróblewski, Janusz Szemraj, Tadeusz Pietras, Aleksandra Jabłkowska, Piotr Czarny

**Affiliations:** 1 Department of Medical Biochemistry, Medical University of Lodz, 92-215 Lodz, Poland; sylwia.ziolkowska@umed.lodz.pl (S.Z.); kinga.jarmusz@student.umed.lodz.pl (K.J.); magdalena.ejsmont@student.umed.lodz.pl (M.E.); adam.wroblewski@umed.lodz.pl (A.M.W.); janusz.szemraj@umed.lodz.pl (J.S.); 2 Department of Clinical Pharmacology, Medical University of Lodz, 90-153 Lodz, Poland; marcin.kosmalski@umed.lodz.pl (M.K.); tadeusz.pietras@umed.lodz.pl (T.P.); 3 Mass Spectrometry Laboratory, Institute of Biochemistry and Biophysics, Polish Academy of Sciences, 02-106 Warsaw, Poland; bianka.swiderska@gmail.com (B.Ś.); agafab@ibb.waw.pl (A.S.); 4 Faculty of Medicine, Lazarski University, 02-662 Warsaw, Poland; 5 Department of Infectious and Liver Diseases, Medical University of Lodz, 91-347 Lodz, Poland; jablkowska@pro.onet.pl

**Keywords:** MASLD, steatosis, DNA repair, mitochondrial DNA, base excision repair

## Abstract

**Highlights:**

**What are the main findings?**

- Patients with MASLD accompanied by insulin resistance differed from healthy controls in mitochondrial and nuclear DNA damage and mtDNA copy number in peripheral blood leukocytes, but these differences may be influenced by age- and BMI-related metabolic status.
- Selected BER-related transcripts and mitochondrial proteins were altered in the MASLD group, suggesting disturbed DNA repair regulation within a broader metabolic phenotype that includes older age and higher BMI.

**What is the implication of the main finding?**

- The findings indicate that mitochondrial DNA maintenance and BER-related alterations in leukocytes may reflect combined effects of MASLD, aging, increased BMI, and systemic metabolic dysfunction rather than MASLD-specific mechanisms alone.

**Abstract:**

Metabolic dysfunction-associated steatotic liver disease (MASLD) is a multifactorial metabolic disorder that is strongly associated with mitochondrial dysfunction and oxidative stress, which may potentially compromise the integrity of mitochondrial DNA (mtDNA). However, the role of the base excision repair (BER) pathway—the main mechanism responsible for repairing oxidative lesions in mitochondria—and maintaining mtDNA stability in MASLD remains poorly understood. Here, we analyzed total mRNA expression levels of key BER components in whole-blood samples, along with mitochondrial protein levels of the selected components. Additionally, we assessed the mtDNA copy number and the damage of mtDNA and nuclear DNA in peripheral leukocytes from MASLD patients and healthy controls. We found that MASLD patients differed from controls in mtDNA and nuclear DNA damage, mtDNA copy number, and selected BER-related markers. However, because the MASLD and control groups also differed substantially in age and BMI, these molecular differences should be interpreted as potentially being associated with age- and BMI-related metabolic status rather than attributable to MASLD alone. While several BER-related genes were downregulated at the mRNA level, the corresponding mitochondrial protein levels were not consistently decreased in MASLD (ProteomeXchange: PXD075974), indicating a discordance between transcriptional and protein-level regulation. These results suggest that altered mitochondrial BER and mtDNA instability in peripheral leukocytes may reflect the combined influence of MASLD, aging, obesity, and broader metabolic dysfunction.

## 1. Introduction

Metabolic dysfunction-associated steatotic liver disease (MASLD), formerly known as non-alcoholic fatty liver disease (NAFLD), has emerged as a major global health concern, particularly in Western countries, where the prevalence of metabolic disorders such as obesity and type 2 diabetes mellitus (T2DM) continues to rise. MASLD is considered the hepatic manifestation of metabolic syndrome and is closely associated with insulin resistance (IR), dyslipidemia, and chronic low-grade inflammation. Despite its high prevalence, MASLD is often overlooked, as the symptoms of the disease are frequently non-specific [1]. Consequently, many patients remain undiagnosed until the disease progresses into more severe conditions, including metabolic dysfunction-associated steatohepatitis (MASH), fibrosis, cirrhosis, and ultimately hepatocellular carcinoma or liver failure [2].

At the molecular level, MASLD is strongly linked to mitochondrial dysfunction. Mitochondria play a central role in hepatic energy homeostasis, fatty acid β-oxidation, and ATP production. Mitochondrial dysfunction may lead to defects in oxidative phosphorylation and electron transport chain (ETC) activity, resulting in an excessive production of reactive oxygen species (ROS). Elevated ROS levels can directly damage cellular macromolecules, including lipids, proteins, and DNA, thereby contributing to oxidative stress and disease progression [3].

Both nuclear DNA (nDNA) and mitochondrial DNA (mtDNA) are susceptible to oxidative damage. Historically, mtDNA was considered particularly vulnerable due to its proximity to the electron transport chain and the absence of protective histones (increasing its exposure to ROS). However, emerging evidence challenges this view, suggesting that the differences in susceptibility between nuclear and mitochondrial genomes may be less explicit than previously assumed [4,5]. Oxidative DNA lesions are primarily repaired by the base excision repair (BER) pathway, which is responsible for maintaining genomic integrity under conditions of oxidative stress [6]. Previous studies have demonstrated altered expression of BER-related genes in animal models with diet-induced hepatic steatosis, suggesting that impaired DNA repair may contribute to MASLD pathogenesis [7,8,9]. Nevertheless, the capacity of BER in people suffering from MASLD remains insufficiently explored.

Current therapeutic strategies for MASLD focus primarily on lifestyle interventions, including adherence to the Mediterranean diet, plant-based or hypocaloric high-protein diets, and increased physical activity [10]. Several pharmacological approaches have demonstrated beneficial effects on hepatic steatosis, inflammation, and fibrosis in MASLD. These include metabolic-targeted therapies such as sodium–glucose cotransporter 2 (SGLT2) inhibitors and pioglitazone, as well as emerging treatments targeting specific disease mechanisms. Recently, resmetirom, a thyroid hormone receptor β agonist, has been approved for the treatment of noncirrhotic MASH with moderate-to-advanced fibrosis, representing a major advancement in MASLD management. In addition, glucagon-like peptide-1 receptor agonists (GLP-1 RAs), including semaglutide, have demonstrated beneficial effects on liver fat accumulation and metabolic parameters, although their role in MASLD treatment continues to evolve [11,12,13,14,15,16]. However, most of these agents target metabolic comorbidities rather than steatosis itself, highlighting that effective MASLD treatment requires a holistic approach encompassing both liver disease and its accompanying metabolic abnormalities. At the same time, a more precise understanding of MASLD-specific molecular mechanisms could facilitate the development of targeted therapeutic approaches aimed directly at impaired DNA repair.

Therefore, the aim of this study was to investigate the level of mRNA and protein expression of genes involved in BER, as well as to evaluate the mtDNA copy number and level of DNA lesions in patients suffering from MASLD. The evaluated genes encode the following proteins: APEX1 (Apurinic/apyrimidinic endonuclease 1), NEIL1 (Nei-like DNA glycosylase 1), POLG (DNA polymerase γ), LIG1 (DNA ligase I), LIG3 (DNA ligase III), XRCC1 (X-ray repair cross-complementing protein 1), PARP1 (poly [ADP-ribose] polymerase 1), FEN1 (Flap endonuclease 1), OGG1 (8-oxoguanine DNA glycosylase), EXOG (exonuclease G), and ENDOG (endonuclease G).

## 2. Materials and Methods

### 2.1. Characteristics of Studied Group

Participants with MASLD were recruited from the Norbert Barlicki Memorial Teaching Hospital in Lodz, Poland. The study group consisted of 99 individuals, while the control group included 30 participants. Hepatic steatosis was confirmed in patients by ultrasonography (USG). Additionally, all participants had been diagnosed with T2DM prior to the detection of steatosis on imaging. Exclusion criteria included individuals below 18 years of age, as well as those with a history of tumors or other liver diseases. Patients diagnosed with MASLD were consecutively recruited from the outpatient clinic during the study recruitment period.

The control group consisted of healthy volunteers without a history of liver disease, diabetes mellitus, or other diagnosed chronic metabolic disorders. A routine complete blood count did not reveal abnormalities indicative of acute or chronic disease. As the controls were recruited as healthy volunteers rather than patients undergoing hepatological evaluation, abdominal ultrasonography was not performed as part of the study protocol. The study protocol was approved by the Bioethics Committee of the Medical University of Lodz, Poland (approval no. RNN/160/20/KE), and written informed consent was obtained from all participants. Due to technical limitations, RNA could not be successfully isolated from all samples, which reduced the group available for gene expression analysis to 82 participants. Additionally, due to the method’s high costs, mass spectrometry analyses were performed only in a subset of 16 patients and 16 controls. Table 1 presents the characteristics of the studied and control groups, while Table 2 demonstrates the clinical and biochemical parameters of MASLD patients.

### 2.2. Sample Collection and Material Isolation

Whole-blood samples were collected into EDTA tubes, aliquoted (200 µL), and stored at −20 °C for DNA isolation and −80 °C for mRNA and protein isolation. DNA was extracted using the Invisorb^®^ Spin Blood Mini Kit (Invitek Molecular GmbH, Berlin, Germany), and mRNA with the NucleoSpin^®^ RNA (Macherey-Nagel GmbH & Co. KG, Düren, Germany). DNA/RNA concentrations and purity were assessed spectrophotometrically at 260/280 nm (Picodrop, Syngen Biotech, Wroclaw, Poland). Leukocytes were isolated from fresh blood by centrifugation (30 min, 400× *g*, 4 °C) in Gradisol L (AquaMed, Warsaw, Poland), washed with PBS, and processed for mitochondrial isolation using the Mitochondria Isolation Kit for Cultured Cells (Thermo Scientific™, Waltham, MA, USA). Proteins were extracted with RIPA buffer and measured by GloMax^®^-Multi+ Detection System (Promega Corporation, Madison, WI, USA) using the Micro BCA™ Protein Assay Kit (Thermo Scientific™, Waltham, MA, USA). All protein samples were stored at −80 °C until further use.

### 2.3. mRNA Level Analysis

mRNA was transcribed to cDNA using the High Capacity cDNA Reverse Transcription Kit (Applied Biosystems, Foster City, CA, USA). Gene expression of BER-related genes (*APEX1*, *NEIL1*, *POLG*, *LIG1*, *LIG3*, *XRCC1*, *PARP1*, *FEN1*, *OGG1*, *EXOG*, and *ENDOG*) was analyzed by fluorescence-based qPCR using ThermoFisher assays (Waltham, MA, USA; IDs listed in Table 3). Reactions were set up in 10 µL volumes with TaqMan™ Universal PCR Master Mix (Applied Biosystems, Foster City, CA, USA). Each sample was run in duplicate, and the average Ct values were used for analysis. *ACTB* was selected as the reference gene based on the BestKeeper method [17] evaluation of three candidates. Relative expression was calculated using the 2^−ΔCt^ method. Thermal cycling was performed on a CFX96 Touch Real-Time PCR System (Bio-Rad, Hercules, CA, USA) under standard conditions (95 °C for 10 min; 40 cycles of 95 °C for 15 s and 60 °C for 1 min).

### 2.4. mtDNA Copy Number Evaluation

The relative mtDNA copy number, expressed as the ratio of mtDNA to nDNA, was determined using fluorescence-based qPCR. The assay IDs used in the study (ThermoFisher, Waltham, MA, USA) are listed in Table 4. Each 10 µL reaction contained 10 ng of gDNA and TaqMan™ Universal PCR Master Mix (Applied Biosystems, Foster City, CA, USA). All reactions were performed in duplicate, and average Ct values were used for further analysis. The mtDNA copy number was quantified using the ΔCt method, where ΔCt = Ct(mtDNA) − Ct(PKM), and calculated as 2(2^−ΔCt^) to evaluate the mtDNA content per cell. The overall mtDNA copy number was measured as the mean of three mitochondrial genes (*mtND1*, *mtND2*, and *mtCO1*), selected according to a previously validated qPCR protocol for mtDNA copy number determination [18]. The use of multiple mitochondrial targets distributed across the mitochondrial genome increases the robustness of mtDNA copy number estimation. The thermal cycling conditions were as follows: initial enzyme activation at 95 °C for 10 min, followed by 40 cycles of denaturation at 95 °C for 15 s, and annealing/extension at 60 °C for 1 min. Reactions were carried out on a CFX96 Touch Real-Time PCR System (Bio-Rad, Hercules, CA, USA), and data were analyzed using Bio-Rad CFX Maestro v1.1 software.

### 2.5. Assessment of DNA Damage

The number of mtDNA lesions was estimated using a semi-long-run real-time PCR technique with some modifications [19,20]. The assay exploits the fact that DNA polymerase activity is inhibited by strand lesions, leading to diminished amplification of damaged templates. Therefore, the number of lesions was calculated based on a Poisson distribution using the following modified equation: number of lesions = − ln (2^ΔCt_short−ΔCt_long)^) [21]. ΔCt_short was defined as the difference between the mean Ct of the sample and the reference obtained by employing primers for 100 bp amplicons, while ΔCt_long referred to the corresponding difference obtained with primers for 1000 bp amplicons. The reference was the sample with the lowest detected DNA damage. The amplicons of nDNA and mtDNA were assessed. The sequences of the primers are presented in Table 5, while the primer design process is presented in Supplementary Data S1. The 20 µL reaction contained 10 ng of gDNA and HOT FIREPol^®^ EvaGreen^®^ qPCR Supermix (Solis BioDyne, Tartu, Estonia). All reactions were performed in duplicate, and average Ct values were used for further analysis. Thermal cycling was carried out on a CFX96 Touch Real-Time PCR System (Bio-Rad, Hercules, CA, USA) according to the manufacturer’s instructions (95 °C for 12 min; 40 cycles of 95 °C for 15 s, 65 °C for 20 s, and 72 °C for 30 s).

### 2.6. Correlation and Regression Analyses

Associations between age or BMI and the investigated molecular parameters were first assessed separately in the MASLD and control groups using Spearman’s rank correlation analysis. Correlation coefficients (r) and corresponding *p*-values were calculated for the mtDNA copy number, mtDNA and nuclear DNA damage, and the mRNA and protein levels of the investigated base excision repair-related factors. Parameters showing a statistically significant correlation with age or BMI (*p* < 0.05) were subsequently considered for additional multivariable linear regression analyses to assess whether the observed differences between the MASLD and control groups persisted after adjustment for the respective clinical variable. These regression analyses were therefore performed only for parameters identified as significantly associated with age or BMI in the preliminary correlation analysis.

To further assess whether associations between molecular parameters and MASLD status were independent of age or BMI, additional multivariable linear regression analyses were performed selectively for parameters that showed statistically significant correlations with age or BMI in the preliminary correlation analyses. Thus, regression models were not applied to all investigated parameters, but only to those for which a significant association with age or BMI had been identified. The molecular parameter was treated as the dependent variable, while the respective clinical variable (age or BMI) was entered as a continuous covariate, and group status (MASLD vs. control) as a categorical predictor. Separate models were constructed for the parameters associated with age and BMI. For parameters requiring transformation to meet the assumptions of linear regression, logarithmic transformation was applied before analysis. Regression coefficients (β), 95% confidence intervals (95% CI), and *p*-values were reported. The statistical significance of the group term was used to assess whether the association between the molecular parameter and MASLD status remained after adjustment for the respective clinical variable.

### 2.7. Mass Spectrometry Sample Preparation

Protein digestion was performed in a 96-well plate format using a modified SP3 (single-pot, solid-phase-enhanced sample preparation) protocol with minor adjustments [22]. Disulfide bonds were reduced with 10 mM tris(2-carboxyethyl)phosphine (TCEP) by incubation for 1 h at 60 °C, followed by alkylation of cysteine residues with 30 mM 2-chloroacetamide (CAA) for 30 min in the dark at room temperature. Magnetic beads were prepared by mixing equal amounts of Sera-Mag Carboxyl hydrophilic and hydrophobic particles (09-981-121 and 09-981-123, GE Healthcare, Cardiff, UK). The bead mixture was added to the samples, and proteins were captured in the presence of 0.1% formic acid in MS-grade acetonitrile, resulting in a final acetonitrile concentration of 85%. After incubation, the bead–protein complexes were collected using a magnetic rack and the supernatant was removed. The beads with bound proteins were washed three times with 87% ethanol, each time separating the beads from the solution using a magnet, followed by two additional washes with 100% acetonitrile. Proteolytic digestion was carried out overnight at 37 °C using 1 µg of sequencing-grade trypsin (Promega) in 100 mM ammonium bicarbonate (NH_4_HCO_3_). After digestion, peptides were eluted from the magnetic beads using 100 µL of MS-grade water. Peptide concentrations were determined using the Pierce Quantitative Colorimetric Peptide Assay (Thermo Scientific) according to the manufacturer’s instructions.

For targeted PRM analysis, 11 proteins were selected. Two proteotypic peptides were chosen for each protein based on established PRM design criteria and information from the PeptideAtlas and UniProt databases. For DNLI1 and APEX1, three peptides were selected to improve quantification robustness. Corresponding stable isotope-labeled (SIS) peptides, synthesized in crude quality, were obtained from JPT Peptide Technologies. The list of SIS standards is provided in Supplementary Data S2.

### 2.8. LC-MS Analysis

Targeted PRM measurements were carried out using the Evosep One–Orbitrap Astral LC–MS platform. Method development and optimization were performed using pooled study samples. During optimization, SIS peptide concentrations were iteratively adjusted to obtain stable MS1 signals in the range of ~1 × 10^7^–1 × 10^9^. Method optimization included the selection of precursor charge states (2+ or 3+), determination of fragment ion patterns, and optimization of MS1 trigger thresholds required for SureQuant acquisition. Fragment ions used for triggering were selected based on signal intensity and specificity, with 6–7 transitions per peptide. For each analysis, 1 µg of digested peptides supplemented with the SIS peptide mixture was loaded onto Evotip Pure C18 cartridges according to the manufacturer’s instructions. Peptides were separated using the 30 SPD method, corresponding to a 44 min gradient. Full MS scans were acquired at a resolution of 120,000 over an *m*/*z* range of 300–900, with a normalized AGC target of 300% and a maximum injection time of 50 ms. The method was operated with a data-dependent cycle time of 7 s. SureQuant acquisition was triggered by the MS1 detection of heavy SIS precursor ions within a ± 6 ppm mass tolerance by using precursor-specific thresholds defined during method optimization. When the trigger threshold was reached, a watch-mode MS2 scan of the SIS peptide was acquired over the 150–1700 *m*/*z* range at a resolution of 7500, with a normalized AGC target of 1000%, maximum injection time of 10 ms, isolation window of 1.0 *m*/*z*, and HCD fragmentation with a normalized collision energy of 27%. Detection of at least three predefined fragment ions was required for successful triggering. Following this event, a high-resolution MS2 spectrum of the corresponding endogenous peptide was recorded at a resolution of 60,000, with a normalized AGC target of 1000% and a maximum injection time of 116 ms. The nanospray source voltage was set to 2.1 kV, and the heated capillary temperature was maintained at 275 °C.

### 2.9. Statistical Analysis

The data were analyzed in SigmaPlot 11.0 (Systat Software, San Jose, CA, USA) and GraphPad Prism 10 (GraphPad Software, Boston, MA, USA). The normality of the data distribution within each group was assessed using the Shapiro–Wilk test, and then, accordingly, Student’s *t*-test or the Mann–Whitney test was used. In gene expression analysis, outliers were identified using the ROUT method (Q = 1%) implemented in GraphPad Prism. Outlier detection was performed independently for each gene. Correlation analysis between the evaluated values (mtDNA copy number, number of mtDNA lesions, gene expression, and levels of the studied proteins) was performed using Pearson’s or Spearman’s correlation.

Targeted SureQuant data were processed using Skyline v24.1 software. Peak integration, manual inspection and correction of chromatographic peaks, and selection of fragment ions for each peptide were performed in Skyline. For most peptides, at least four fragment ions were selected for quantification, with the exception of the peptide HVLDALDPNAYEAFK. One of the three monitored peptides for DNLI1 (DIEQIAEFLEQSVK) was excluded from further analysis due to a substantially lower signal quality compared with the remaining peptides. All three peptides selected for APEX1 were retained, while both peptides selected for the remaining proteins were included in the final analysis. Peptide-level quantitative values were exported from Skyline as ratios between endogenous (light) peptide peak areas and the corresponding SIS peptide peak areas (ratio-to-standard). These ratios were subsequently normalized by dividing by the highest observed value for each peptide, resulting in values scaled between 0 and 1. Protein-level abundances were calculated as the geometric mean of the corresponding peptide values. Statistical analyses were performed in Perseus, where descriptive statistics were calculated, and group comparisons were carried out using Student’s *t*-test.

## 3. Results

### 3.1. The Expression of BER Genes on Transcriptional Level Is Modulated in MASLD

**Figure 1:**
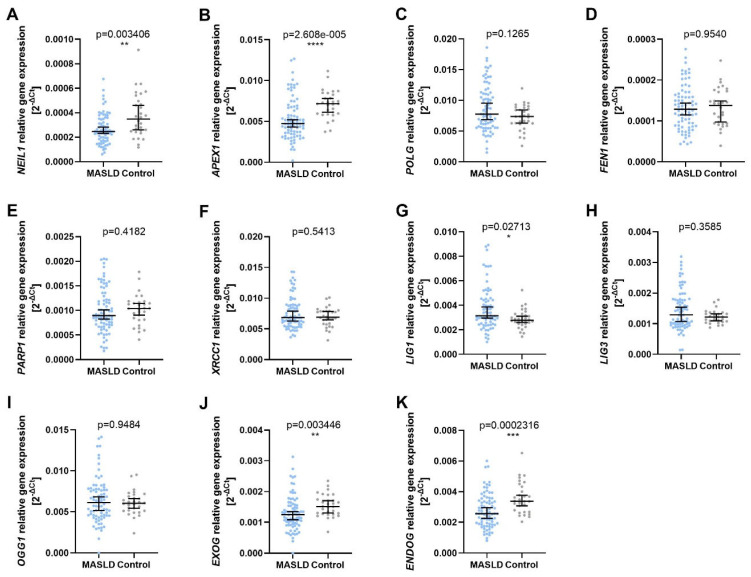
The altered expression of genes *NEIL1*, *APEX1*, *POLG*, *FEN1*, *PARP1*, *XRCC1*, *LIG1*, *LIG3*, *OGG1*, *EXOG*, and *ENDOG* (**A**–**K**) in peripheral blood cells, calculated using the 2^−ΔCt^ method (according to *ACTB* gene expression as reference) and presented as scatter dot plots (MASLD as blue and grey as controls); horizontal lines represent the median, and whiskers denote 95% CI in MASLD patients group in comparison to healthy controls. Samples that were identified as outliers using the ROUT method (Q = 1%) were excluded from the analysis. The number of samples included in the analysis is as follows: (**A**) *n*_MASLD_ = 72, *n*_control_ = 30; (**B**) *n*_MASLD_ = 81, *n*_control_ = 30; (**C**) *n*_MASLD_ = 79, *n*_control_ = 30; (**D**) *n*_MASLD_ = 79, *n*_control_ = 30; (**E**) *n*_MASLD_ = 82, *n*_control_ = 30; (**F**) *n*_MASLD_ = 77, *n*_control_ = 30; (**G**) *n*_MASLD_ = 71, *n*_control_ = 30; (**H**) *n*_MASLD_ = 77, *n*_control_ = 28; (**I**) *n*_MASLD_ = 80, *n*_control_ = 30; (**J**) *n*_MASLD_ = 80, *n*_control_ = 30; (**K**) *n*_MASLD_ = 82, *n*_control_ = 30. * *p* < 0.05; ** *p* < 0.01; *** *p* < 0.001; **** *p* < 0.0001. MASLD—metabolic dysfunction-associated steatotic liver disease.

Analysis of gene expression at the transcriptional level revealed differential regulation of selected components of the BER pathway in patients with MASLD compared with healthy controls (Figure 1). However, because the MASLD and control groups differed substantially in age and BMI, these group differences should be interpreted with caution and may reflect age- and BMI-related metabolic differences in addition to MASLD status. Among the eleven analyzed transcripts, expression of *NEIL1* (*p* < 0.01), *APEX1* (*p* < 0.0001), *EXOG* (*p* < 0.01), and *ENDOG* (*p* < 0.001) was significantly reduced in the MASLD group, whereas *LIG1* (*p* < 0.05) expression was significantly increased. In contrast, no significant differences in mRNA levels were observed for *POLG*, *FEN1*, *PARP1*, *LIG3*, *XRCC1*, or *OGG1* between the two groups.

### 3.2. The Protein Levels of BER Components Are Altered in MASLD

**Figure 2:**
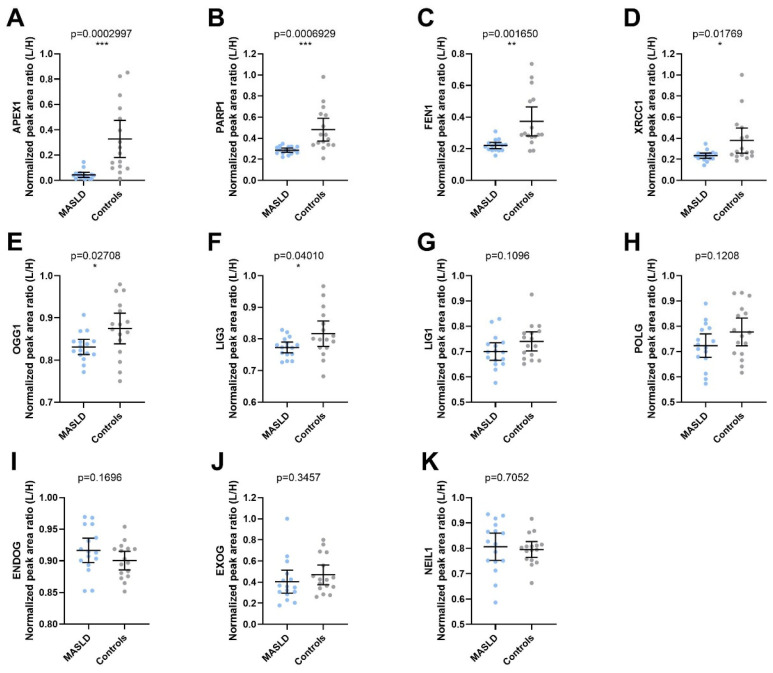
Protein relative content quantified using stable isotope-labeled peptide standards. Protein levels of base excision repair (BER)-related factors in leukocyte mitochondria from patients with MASLD and controls. (**A**) APEX1; (**B**) PARP1; (**C**) FEN1; (**D**) XRCC1; (**E**) OGG1; (**F**) LIG3; (**G**) LIG1; (**H**) POLG; (**I**) ENDOG; (**J**) EXOG; (**K**) NEIL1. Scatter dot plots (MASLD as blue and grey as controls) show protein levels in mitochondrial extracts obtained from the control group and patients with MASLD. Each point represents an individual sample (*n* = 16 per group), and the horizontal line indicates the group’s mean. Statistical comparisons between groups were performed using Student’s *t*-test. * *p* < 0.05; ** *p* < 0.01; *** *p* < 0.001. MASLD—metabolic dysfunction-associated steatotic liver disease.

Targeted PRM analysis was used to quantify the mitochondrial levels of 11 proteins associated with the BER pathway, including XRCC1, APEX1, DPOG1, PARP1, OGG1, NEIL1, DNLI3, DNLI1, FEN1, EXOG, and NUCG, in leukocyte-derived mitochondrial extracts from control subjects and MASLD patients. Statistically significant differences between the groups were observed for several BER-related proteins. Specifically, the mitochondrial levels of XRCC1, APEX1, PARP1, OGG1, DNLI3, and FEN1 were significantly lower in MASLD patients compared with the controls. Nevertheless, given the marked differences in age and BMI between groups, these protein-level alterations may be related to the broader metabolic phenotype of the MASLD group, including aging and obesity-related mitochondrial changes, and should not be interpreted as MASLD-specific effects. In contrast, no statistically significant differences were detected for the remaining proteins. Individual sample distributions and group means for all proteins are presented as scatter plots in Figure 2A–K.

### 3.3. Clinical Parameters of Glucose and Lipid Metabolism and Oxidative Stress Are Modified in MASLD

**Figure 3:**
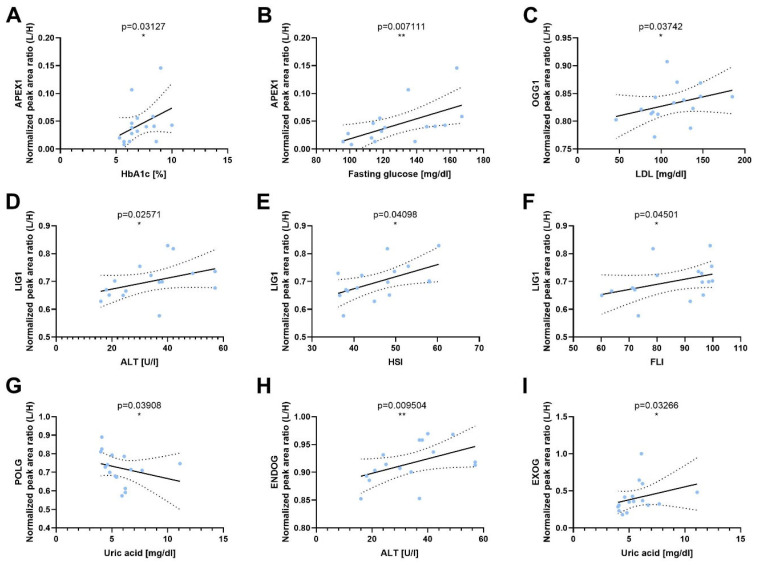
Spearman’s correlations between (**A**) the normalized peak area ratio of APEX1 and HbA1c; (**B**) the normalized peak area ratio of APEX1 and fasting glucose level; (**C**) the normalized peak area ratio of OGG1 and LDL level; (**D**) the normalized peak area ratio of LIG1 and ALT level; (**E**) the normalized peak area ratio of LIG1 and HSI; (**F**) the normalized peak area ratio of LIG1 and FLI; (**G**) the normalized peak area ratio of POLG and uric acid level; (**H**) the normalized peak area ratio of ENDOG and ALT level; and (**I**) the normalized peak area ratio of EXOG and uric acid level. Scatter dot plots show protein levels in mitochondrial extracts obtained from the control group and patients with MASLD. Each point represents an individual sample (*n* = 16), and a linear regression line was fitted to the data, with dotted lines indicating the 95% CI. * *p* < 0.05; ** *p* < 0.01. ALT—alanine aminotransferase; FLI—fatty liver index; HSI—hepatic steatosis index; LDL—low-density lipoprotein.

Spearman’s correlation analysis showed positive correlations between several studied proteins and clinical parameters. Significant positive correlations were observed between several proteins and HbA1c, fasting glucose, LDL, ALT, HSI, FLI, and uric acid (Figure 3A–F,H,I). In contrast, the POLG protein level was negatively correlated with uric acid (Figure 3G). All non-significant results are presented in the Supplementary Data S3A.

### 3.4. mtDNA Copy Number and Number of DNA Lesions Are Modulated in MASLD

**Figure 4:**
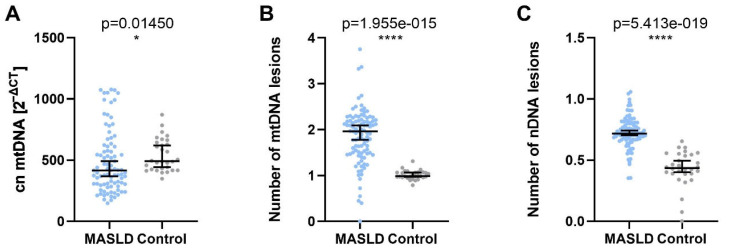
(**A**) Copy number of mtDNA calculated using the 2^−ΔCt^ method. (**B**) Number of DNA lesions in mitochondria, based on a modified Poisson distribution. (**C**) Number of DNA lesions in the nucleus, based on a modified Poisson distribution. The changes in peripheral blood cells are presented as scatter dot plots; horizontal lines represent the median, and whiskers denote 95% CI in the MASLD patient group in comparison to healthy controls. Samples that were identified as outliers using the ROUT method (Q = 1%) were excluded from the analysis. The number of samples included in the analysis is as follows: (**A**) *n*_MASLD_ = 87, *n*_control_ = 30; (**B**) *n*_MASLD_ = 99, *n*_control_ = 29; (**C**) *n*_MASLD_ = 94, *n_c_*_ontrol_ = 30. * *p* < 0.05; **** *p* < 0.0001. Cn mtDNA—copy number of mitochondrial DNA; MASLD—metabolic dysfunction-associated steatotic liver disease.

Compared with the control group, patients with MASLD exhibited a significantly decreased mtDNA copy number. Furthermore, the MASLD group demonstrated a higher number of DNA lesions in both mitochondrial and nuclear genomes. Specifically, the median number of DNA lesions was nearly two-fold higher in mitochondrial DNA (median MASLD = 1.960; median controls = 0.9878) and more than 1.5-fold higher in nDNA (median MASLD = 0.7172; median controls = 0.4356) (Figure 4). Because the MASLD group was older and had higher BMIs than the controls, these differences may be influenced by age- and BMI-related mitochondrial dysfunction and oxidative stress, in addition to MASLD-associated metabolic alterations.

### 3.5. Age and BMI Differentially Influence the Associations Between MASLD and Molecular Parameters

Spearman’s correlation analyses were performed separately in the MASLD and control groups to assess the relationships between age or BMI and the investigated molecular parameters. In the MASLD group, age was significantly negatively correlated with OGG1 protein levels, whereas no other significant associations with age or BMI were observed. In the controls, age was positively correlated with nuclear DNA damage and negatively correlated with XRCC1 and LIG3 mRNA levels. BMI was negatively correlated with the mtDNA copy number and positively correlated with nuclear DNA damage. These parameters were subsequently included in additional regression analyses performed in the entire cohort (Table 6).

The regression analyses showed that the association between MASLD status and mtDNA copy number remained significant after an adjustment for age, whereas this association was not significant after an adjustment for BMI. The association between MASLD status and nuclear DNA damage remained highly significant after an adjustment for BMI. In contrast, no statistically significant associations between group status and XRCC1 and LIG3 mRNA levels, or OGG1 protein levels, were observed after an adjustment for age (Table 7). Overall, these analyses indicate that the influence of age and BMI on the investigated molecular parameters was not uniform across the studied markers.

### 3.6. The Relative Expression of BER Genes Is Correlated with Copy Number of mtDNA and mtDNA Damage

**Figure 5:**
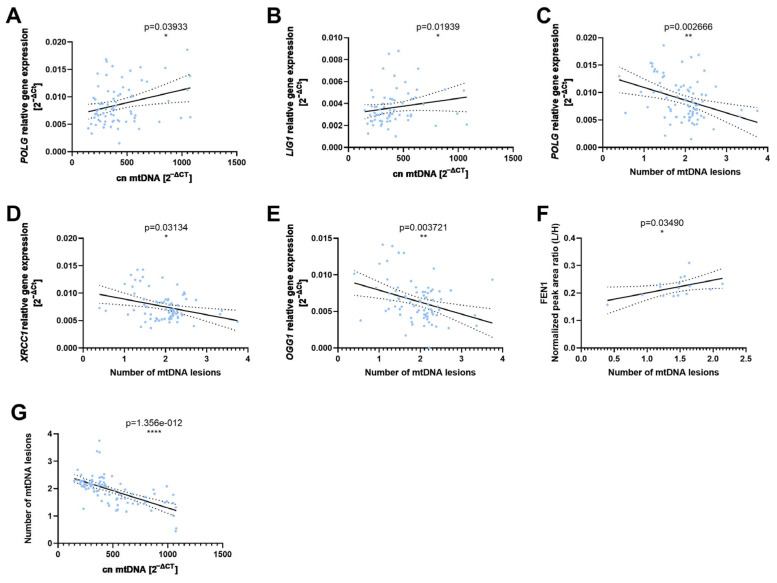
The Spearman’s correlation between (**A**) copy number of mtDNA (cn mtDNA) and relative gene expression of *POLG*; (**B**) cn mtDNA and relative gene expression of *LIG1*; (**C**) relative gene expression of *POLG* and number of DNA lesions in mitochondria (number of mtDNA lesions); (**D**) relative gene expression of *POLG* and number of mtDNA lesions; (**E**) relative gene expression of *OGG1* and number of mtDNA lesions; (**F**) normalized peak area ratio of FEN1 and number of mtDNA lesions; (**G**) cn mtDNA and number of mtDNA lesions. Analyses were performed in the leukocytes of the MASLD patient group. The cn mtDNA and relative expression were calculated using the 2^−ΔCt^ method, while the number of DNA lesions was based on a modified Poisson distribution. A linear regression line was fitted to the data, with dotted lines indicating the 95% CI. * *p* < 0.05; ** *p* < 0.01; **** *p* < 0.0001. Cn mtDNA—copy number of mitochondrial DNA; MASLD—metabolic dysfunction-associated steatotic liver disease.

Spearman’s correlation analysis was used to examine the relationships between gene expression, mtDNA copy number, and DNA damage in mitochondrial and nuclear genomes. Significant positive correlations were observed between the mtDNA copy number and the expression of *POLG* and *LIG1* (Figure 5A,B). Subsequently, significant negative correlations were found between the number of mtDNA lesions and the expression of *POLG*, *XRCC1*, and *OGG1* (Figure 5C–E). Furthermore, the mtDNA copy number was found to be significantly negatively correlated with the number of mtDNA lesions (Figure 5F). In contrast, no significant correlations were observed between the mtDNA copy number and nDNA damage, nor between mitochondrial and nuclear DNA lesions. Likewise, gene expression levels were not significantly correlated with the number of nDNA lesions. All statistically significant correlations are shown in Figure 5A–G, while non-significant results are presented in Supplementary Data S3B.

## 4. Discussion

MASLD is associated with increased oxidative stress and mitochondrial dysfunction, which promotes oxidative damage to both nuclear and mtDNA. In line with this concept, our results demonstrate increased levels of both mtDNA and nDNA damage in leukocytes from patients with MASLD. However, these findings should be interpreted in the context of the substantially higher age and BMI of the MASLD group compared with the controls, as both aging and obesity are independently associated with oxidative stress, mitochondrial dysfunction, and DNA damage. Therefore, the observed differences may reflect the combined influence of MASLD, increased BMI, aging, and accompanying metabolic disturbances rather than MASLD alone. A previous study reported enhanced oxidative mitochondrial metabolism in PBMCs from NAFLD, currently referred to as MASLD, suggesting that increased mitochondrial activity may contribute to mtDNA damage in these circulating cells [23]. On the other hand, elevated levels of DNA damage have also been observed in liver tissue in NAFLD, including increased mtDNA and nDNA lesions in hepatocytes. The alterations in both peripheral blood cells and hepatocytes highlight the systemic nature of oxidative and metabolic stress in this disease [24,25]. Notably, in our study, nDNA damage detected in leukocytes correlated with neither mtDNA damage nor the mtDNA copy number. This lack of association may reflect distinct sources and regulatory mechanisms of genomic injury, whereby nuclear and mitochondrial DNA damage occur independently and do not necessarily follow the same pattern.

The mtDNA copy number is an important aspect of mitochondrial genome maintenance, often reflecting a compensatory response to damage. Cells can increase mtDNA replication to preserve mitochondrial function despite accumulated lesions, and the presence of multiple mtDNA copies per cell provides a functional reserve that buffers against mitochondrial dysfunction. In our study, the mtDNA copy number in leukocytes from MASLD patients was lower than in controls, consistent with a previous report showing a reduced copy number of mtDNA in the blood of NAFLD patients [26]. Interestingly, the situation in liver tissue appears different: studies in human liver and in high-fat diet mouse models have reported an increased mtDNA copy number, suggesting tissue-specific regulation of mitochondrial biogenesis [27,28,29]. It is important to note that the mtDNA copy number reflects the balance between mitochondrial biogenesis and degradation rather than damage per se. However, in our data, a lower mtDNA copy number was associated with greater mtDNA damage. A decreased mtDNA copy number may exacerbate mitochondrial genome vulnerability because fewer copies reduce functional redundancy, leaving each damaged molecule more likely to impair mitochondrial function. Consequently, when the mtDNA copy number is reduced, the impact of oxidative lesions is amplified, leading to greater overall mtDNA damage [30].

The first enzymes acting in the BER pathway are DNA glycosylases, which are responsible for the recognition and removal of damaged bases. In the present study, we focused on NEIL1, a bifunctional DNA glycosylase, and we observed reduced *NEIL1* expression in leukocytes from patients with MASLD. Unlike monofunctional glycosylases, NEIL1 possesses lyase activity and cleaves the N-glycosidic bond, generating abasic (AP) sites as intermediates of the BER pathway [31]. Our previous study showed a link between single nucleotide polymorphism (SNP) in *NEIL1* and the risk of NAFLD onset [32]. Furthermore, other studies have shown that double knockout of *NEIL1* results in obesity, dyslipidemia, hepatic steatosis, and hyperinsulinemia. These metabolic disturbances are accompanied by increased mtDNA damage and a higher susceptibility to hepatocellular carcinoma, underscoring the protective role of NEIL1 in metabolic syndrome-related disorders [33,34]. Importantly, BER intermediates can be more cytotoxic than the original DNA lesions; in particular, unrepaired AP sites may lead to double-strand breaks, which are more difficult to repair due to the requirement of more complex pathways. Notably, although *NEIL1* expression was reduced at the transcript level in MASLD, no corresponding changes were observed in mitochondrial NEIL1 protein levels. It should be noted that gene expression was assessed in whole leukocytes, whereas protein abundance was measured specifically in the mitochondrial fraction, which may contribute to the observed difference. The discrepancy in findings suggests the involvement of compensatory mechanisms at the post-transcriptional or translational level, such as increased translation efficiency or enhanced protein stability. In conditions of cellular stress, mitochondria may modulate the post-transcriptional regulation of their composition. This may indicate a tendency to preserve adequate levels of proteins involved in essential processes such as DNA repair under stress conditions [35]. Maintenance of NEIL1 protein levels may reflect the need to preserve basal glycosylase activity and ensure continuity of BER under conditions of mitochondrial stress [36].

OGG1 is a bifunctional DNA glycosylase and is responsible for the removal of 7,8-dihydro-8-oxoguanine (8-oxoG), one of the most prevalent oxidative DNA lesions. In the present study, reduced mitochondrial OGG1 protein levels in MASLD patients may indicate a diminished capacity to repair oxidative damage within mtDNA, which is consistent with the increased mtDNA damage observed in this group. Experimental evidence supports a broader role of OGG1 in metabolic regulation. Mice lacking *OGG1* (*Ogg1*−/−) exhibit increased susceptibility to age- and diet-induced obesity, accompanied by impaired hepatic metabolism, as demonstrated by a reduced expression of fatty acid oxidation-related genes [37,38,39]. In contrast, transgenic models with mitochondrially targeted overexpression of OGG1 are protected against high-fat diet-induced metabolic disturbances, displaying lower body weight, reduced plasma glucose and insulin levels, decreased lipid accumulation, and improved insulin sensitivity [40]. Notably, OGG1 has also been implicated in the regulation of PARylation, because its deficiency leads to reduced PARP activity and enhanced adipogenic differentiation, while overexpression exerts the opposite effect [39]. Consistently, in vitro studies demonstrate that *OGG1* knockdown in hepatocyte models promotes lipid accumulation and reduces β-oxidation capacity under fatty acid exposure [41]. Interestingly, in our study, the observed decrease in OGG1 protein levels was not accompanied by changes in its mRNA expression, indicating a potential dissociation between transcriptional and translational regulation. This is further supported by the opposite pattern observed for NEIL1, where reduced mRNA levels were detected in MASLD despite unchanged protein abundance. Beyond alterations in transcriptional and translational regulation, additional mechanisms may contribute to the observed changes, including impaired protein import into mitochondria. Recent studies indicate that oxidative stress, which is a well-established feature of hepatic steatosis, can trigger the intermembrane space unfolded protein response (IMS-UPR). ROS can induce oxidative modifications of amino acid residues, particularly highly susceptible cysteine and methionine residues, thereby affecting the proteins involved in the mitochondrial import machinery [42,43]. These proteins are components of complexes responsible for protein translocation, located in both the outer and inner mitochondrial membranes, and their oxidative modification may impair proper function [42]. Together, these findings suggest that regulation of BER components in MASLD may occur at several levels of protein production and transport rather than being driven solely by transcriptional changes.

APEX1 acts immediately downstream of DNA glycosylases by cleaving AP sites and is therefore involved in nearly all subpathways of BER. The main exception is short-patch BER (SP-BER), initiated by the bifunctional glycosylases NEIL1 and NEIL3, where AP sites are processed by PNKP instead of APEX1 [44]. In the present study, *APEX1* expression was reduced in leukocytes from patients with MASLD, consistent with a decreased mitochondrial protein level. This consistent downregulation at both the transcript and protein levels suggests impaired processing of BER intermediates and may suggest compromised repair capacity in MASLD. Notably, complete loss of *APEX1* is embryonically lethal, highlighting its essential role in genome maintenance, while increased *APEX1* expression has been reported in hepatocellular carcinoma [6,45]. We showed in a previous study that altered genetic variants of *APEX1* may modulate the risk of NAFLD occurrence [32]. APEX1 closely cooperates with PARP1 in both short- and long-patch BERs (LP-BER) following the action of monofunctional glycosylases. PARP1 is among the earliest sensors of DNA single-strand breaks and AP sites, and its PARylation activity promotes the recruitment of key repair factors. Beyond BER, PARP1 also participates in the other pathways responsible for repairing both single-strand and double-strand breaks [46]. The upregulated PARP1 has also been associated with hepatocellular carcinoma [47], highlighting its complex role in MASLD pathophysiology. It can be observed that PARP1 activity is increased in hepatocytes from NAFLD patients as well as in mice with induced fatty livers, implicating PARP1 involvement in lipotoxicity and steatohepatitis [46,48,49,50,51]. In contrast to these reports, we did not observe any differences in *PARP1* expression in leukocytes. In parallel to OGG1, PARP1 exhibited a similar pattern, with unchanged transcript levels but significantly decreased protein levels in the mitochondrial fraction. The reduction of PARP1 protein despite stable mRNA levels points to post-transcriptional regulation, such as impaired translation efficiency, enhanced proteasomal or mitochondrial degradation, or defects in protein import into mitochondria [36,52].

Pol γ, whose catalytic subunit is encoded by *POLG*, is the only DNA polymerase operating in mammalian mitochondria and is therefore essential for both mtDNA replication and repair [53]. During SP-BER, Pol γ removes the 5′-deoxyribose phosphate moieties generated by APEX1 through its lyase activity and subsequently inserts a single nucleotide to fill the gap, whereas in LP-BER, it incorporates 2–11 nucleotides via strand displacement synthesis [31]. In the present study, we did not observe differences in *POLG* expression between MASLD patients and controls, which was paralleled by unchanged mitochondrial protein levels. This consistency suggests that both transcriptional and translational regulation of Pol γ may be preserved in MASLD. Nevertheless, pathogenic *POLG* variants represent a major genetic cause of mitochondrial hepatopathies, and POLG deficiency has been associated with impaired liver function. These disorders are considered nuclear-encoded mitochondrial diseases, and hepatic involvement is a frequent clinical feature [54,55,56,57].

FEN1 functions downstream of Pol γ only in LP-BER by cleaving the 5′ flap generated during strand displacement synthesis [31]. Although *FEN1* expression did not differ between the MASLD patients and controls at the transcript level, mitochondrial FEN1 protein levels were significantly reduced in the MASLD patients. This discrepancy is similar to *PARP1* expression, which may suggest post-transcriptional or post-translational regulation or impaired mitochondrial import of FEN1, which may contribute to compromised BER capacity within mitochondria despite preserved mRNA levels. Nevertheless, complete loss of *FEN1* results in early embryonic lethality [58,59], demonstrating its essential role in genome stability, while altered *FEN1* expression has been associated with poor prognosis in certain cancers, such as breast cancer [60].

DNA ligases catalyze the final step of BER by sealing DNA strand interruptions and restoring genome integrity. In mammalian cells, LIG1 and LIG3 fulfill distinct but complementary roles, with LIG1 participating mainly in nuclear BER and DNA replication, while LIG3 acts as the primary ligase in mitochondrial BER in cooperation with the scaffold protein XRCC1 [6,31,61]. In the present study, LIG1 transcripts were elevated in the MASLD patients, yet protein levels remained unchanged, whereas XRCC1 and LIG3 proteins were significantly reduced despite stable mRNA expression. Together, these results reinforce the notion that post-transcriptional regulation or impaired mitochondrial targeting plays a key role in modulating BER capacity in MASLD. The upregulation of *LIG1* mRNA may reflect a compensatory response to elevated DNA damage, consistent with reports showing increased hepatic *LIG1* expression in mice fed a methionine- and choline-deficient diet [62]. Importantly, *LIG1* deficiency leads to genome instability, and its increased expression may also relate to functions beyond mitochondria, including DNA replication and general genome maintenance [63].

Furthermore, evidence from hepatocellular carcinoma (HCC) indicates that alterations in the *XRCC1* gene may emerge during disease progression rather than in early metabolic liver injury [64,65,66,67]. Importantly, studies report both an increase and decrease of *XRCC1* expression in HCC [68,69], pointing to a context-dependent role of this scaffold protein that may vary with tumor stage, molecular subtype, or cellular compartment. Moreover, SNPs in *LIG3* have been shown to modulate NAFLD risk [32]. Together, these observations indicate a similar pattern to OGG1, PARP1, and FEN1, further supporting the notion that multiple BER factors are regulated beyond the transcriptional level in MASLD. Consequently, disruption of key BER components may compromise the efficiency of the repair process, which may contribute to the accumulation of DNA damage and progression of mitochondrial dysfunction.

Mitochondrial nucleases ENDOG and EXOG, despite belonging to the same enzyme family, appear to play distinct roles in cellular homeostasis. In our study, mitochondrial levels of both proteins in leukocytes did not differ between groups, which precludes confirmation of their involvement in MASLD pathophysiology. It is worth evaluating whether such nucleases contribute to liver pathology through intracellular activity rather than within mitochondria themselves. However, mRNA levels were decreased, which may overall be consistent with the assumption that post-transcriptional or post-translational modulations may influence the final abundance. ENDOG classically is associated with apoptosis through nuclear translocation, but has been implicated in metabolic regulation [70]. ENDOG can transfer to the endoplasmic reticulum, where it interacts with BiP and activates the IRE1α/PERK pathways, promoting ER stress and lipid synthesis via increased acetyl-CoA production. In line with this, ENDOG depletion has been shown to reduce lipogenesis and alleviate diet-induced steatosis production [71]. Conversely, ENDOG deficiency has also been linked to hepatic fat accumulation through impaired mitochondrial function, specifically via reduced oxidative phosphorylation [72]. In contrast, EXOG functions primarily as a mitochondrial DNA repair enzyme and has not been directly linked to liver disease or lipid metabolism. It plays a critical role in mitochondrial BER, exhibiting strong 5′-dRP removal activity and participating in the processing of flap structures generated during LP-BER [73,74,75]. Although its role in metabolic regulation appears indirect, impaired EXOG function may contribute to mitochondrial dysfunction, a key feature of MASLD pathogenesis. Thus, while ENDOG may act as a regulator linking mitochondrial function with metabolic signaling, EXOG represents a core component of the mtDNA repair machinery, supporting cellular homeostasis at the level of genome maintenance.

Aside from comparing the groups, we analyzed the correlations between protein levels and clinical parameters in patients. Despite the relatively small size of the analyzed cohort, the observed correlations between selected mitochondrial BER-related proteins and clinical parameters may provide insight into the systemic response of leukocytes to metabolic stress in MASLD. These associations appeared to cluster around several pathophysiological domains. Namely, the parameters for glucose metabolism, including HbA1c and fasting glucose, were positively correlated with APEX1 protein levels, whereas the markers related to lipid metabolism or hepatic steatosis, such as LDL cholesterol, ALT, HIS, and FLI, were associated with OGG1, LIG1, and EXOG [76,77]. In addition, uric acid, a parameter closely associated with oxidative stress, showed opposite associations with EXOG and POLG [78]. These correlations may suggest that increased levels of selected BER and mitochondrial maintenance proteins could possibly reflect a compensatory response to metabolic and oxidative burden. In this context, the positive associations between APEX1, OGG1, LIG1, and EXOG and the clinical indicators of glucose and lipid dysregulation may reflect an adaptive upregulation of mtDNA repair pathways in response to increased metabolic stress [79]. Importantly, because these measurements were performed in peripheral leukocytes rather than liver tissue, the observed changes should be considered as markers of a systemic component of MASLD rather than direct evidence of hepatic BER activity. Nevertheless, this systemic pattern is consistent with the concept that MASLD is not restricted to the liver but involves broader metabolic and inflammatory disturbances, which might affect circulating cells [80]. The inverse association between POLG and uric acid may point to a second axis of the cellular response. While the positive correlation between EXOG and uric acid fits the hypothesis of enhanced repair-related compensation under oxidative stress, the negative correlation between POLG and uric acid may suggest suppression of mtDNA replication when oxidative burden increases [81]. This interpretation is consistent with the reduced mtDNA copy number observed in MASLD patients in the present study. Thus, leukocytes may simultaneously attempt to preserve mitochondrial genome integrity by increasing selected repair-associated proteins while limiting mtDNA replication under conditions in which newly synthesized mitochondrial genomes could be prone to damage or instability.

A potential concern in this study is the substantial difference in age and BMI between the MASLD and control groups, as both factors may influence molecular markers of DNA maintenance. To address this issue, we first examined the associations of age and BMI with the investigated parameters separately within each group and subsequently performed additional regression analyses for the parameters showing significant correlations. These analyses indicated that the influence of age and BMI was not uniform across the investigated markers. Notably, the association between MASLD status and mtDNA copy number remained significant after adjusting for age, suggesting that the observed reduction in mtDNA copy number cannot be attributed solely to the older age of the MASLD group. Similarly, the association between MASLD status and nuclear DNA damage remained highly significant after adjusting for BMI, despite the higher BMI observed in patients with MASLD. In contrast, the differences in some BER-related parameters, including the expression of *XRCC1*, *LIG3*, and *OGG1*, were no longer statistically significant after adjusting for age. These findings suggest that age and BMI may contribute to selected molecular alterations, while the associations of MASLD with mtDNA copy number and nuclear DNA damage appear to be more robust to adjustments for these potential confounders. Nevertheless, given the substantial age imbalance and the relatively limited number of controls, residual confounding cannot be completely excluded, and the findings should therefore be interpreted with appropriate caution.

Our correlation analyses revealed that the mtDNA copy number was positively associated with the expression of *POLG* and *LIG1*, two factors that also play roles in DNA replication and genome maintenance. This relationship is unlikely to reflect gene dosage effects, as both proteins are nuclear encoded, but rather suggests coordinated regulation of mitochondrial biogenesis and DNA repair capacity. In parallel, mtDNA damage showed a negative correlation, not only with *POLG* expression but also with *XRCC1* and *OGG1*, supporting the concept that reduced availability of multiple BER factors may compromise repair efficiency and facilitate the accumulation of oxidative lesions within the mitochondrial genome. Importantly, POLG emerged as the only BER component linked to both mtDNA copy number and mtDNA damage, highlighting its dual role at the interface of repair and replication. Furthermore, the observed inverse relationship between mtDNA copy number and mtDNA damage supports the notion that impaired mitochondrial genome maintenance contributes to lesion accumulation. Together, these findings suggest that dysregulation of mitochondrial BER components, particularly those involved in DNA synthesis and repair coordination, may affect not only lesion removal but also the maintenance of mtDNA abundance. This pattern supports the concept of impaired mitochondrial maintenance, rather than increased lesion formation alone, as a key contributor to genomic instability in MASLD.

Although this study provides valuable insights, several limitations must be acknowledged. First, the relatively small sample size may restrict the generalizability of our findings. However, this cohort was homogeneous and rigorously characterized, which minimized confounding variables. Second, the even smaller group selected for mass spectrometry analyses could limit the depth of our proteomic-based conclusions. We mitigated this by utilizing a valid, high-resolution mass spectrometry pipeline with robust internal controls, ensuring that the generated proteomic profiles remain highly reliable and reproducible despite the smaller sample size. Third, the number of samples differed between individual experimental analyses due to technical constraints and sample availability, which may introduce variability across datasets. However, each experiment was initially processed and analyzed independently according to method-specific analytical pipelines. Subsequently, integrative correlation analyses were performed to explore relationships between the molecular parameters measured across different biological levels. Most importantly, the lack of individual matching between MASLD patients and controls, particularly regarding age and BMI, should be acknowledged as a major limitation of the study, as both factors may influence metabolic status, oxidative stress, mitochondrial function, mtDNA copy number, DNA damage, and BER-related pathways.

MASLD is closely linked to multiple metabolic disturbances, including obesity, IR, diabetes, dyslipidemia, and aging, each of which may independently affect mitochondrial function and DNA repair pathways. Therefore, the observed impairment of mitochondrial BER should be interpreted as being associated with the MASLD metabolic phenotype rather than as being definitively attributable to MASLD alone. Although additional analyses did not indicate that age or BMI fully explained the observed alterations, the independent contributions of individual comorbidities and metabolic factors cannot be completely excluded. Future studies, including larger cohorts, carefully matched controls, and stratification according to specific metabolic comorbidities, will be required to clarify which components of the MASLD spectrum contribute most strongly to mtDNA repair impairment.

Our molecular analyses were performed on peripheral blood leukocytes rather than liver tissue. While liver biopsies provide the most direct insight into hepatic metabolism, obtaining them from all participants presents major ethical and logistical complications. Conversely, leukocytes serve as a minimally invasive marker that reflects systemic inflammation and metabolic stress associated with liver disease. In MASLD, hepatocyte lipid accumulation and metabolic dysfunction are accompanied by systemic metabolic and inflammatory alterations that extend beyond the liver [3]. Increased circulating levels of free fatty acids, glucose, pro-inflammatory cytokines, and ROS may influence mitochondrial homeostasis in peripheral immune cells [30]. Chronic exposure of leukocytes to this altered metabolic environment may affect mitochondrial DNA stability and the activity of DNA repair pathways, including BER mechanisms [23,26]. Therefore, the alterations observed in peripheral blood leukocytes may reflect a systemic response to MASLD-associated metabolic stress rather than a liver-specific mitochondrial defect.

Our conclusions are based on the correlations between levels of total mRNA transcription and the mitochondrial fraction of proteins. Due to the limited number of samples, we had to choose between analyzing protein levels in whole cells or specifically in mitochondria. We therefore focused on the mitochondrial fraction, as whole-cell measurements would not allow for the direct evaluation of processes occurring within mitochondria. This is because whole-cell protein abundance may not accurately reflect compartment-specific changes. Such discrepancies are likely influenced by post-transcriptional and post-translational regulation, as well as potential alterations in protein transport into the mitochondria. Another limitation of the present study is related to the characterization of the control group. Controls were recruited as healthy volunteers without a history of diagnosed liver disease, diabetes mellitus, or other metabolic disorders, and routine clinical assessment was performed before inclusion. However, abdominal ultrasonography was not performed in control participants; therefore, the presence of undiagnosed subclinical hepatic steatosis cannot be completely excluded.

Finally, the diagnosis of MASLD was established via abdominal USG rather than liver biopsy, which remains the diagnostic gold standard but is invasive. In this study, abdominal ultrasonography was used to establish MASLD status because it is widely available, non-invasive, and feasible for evaluating large cohorts, despite its known limitations in detecting early steatosis and grading fibrosis.

## 5. Conclusions

In summary, our findings suggest that patients with MASLD differed from healthy controls in mitochondrial genome maintenance in peripheral leukocytes, reflected by increased mtDNA and nuclear DNA damage, reduced mtDNA copy numbers, and altered regulation of BER-related factors at both transcript and protein levels. However, because the MASLD group was older and had a higher BMI than the control group, these alterations may be related to age- and BMI-associated metabolic dysfunction as well as to MASLD itself. The discrepancy between mRNA expression and mitochondrial protein levels indicates that post-transcriptional regulation, protein stability, degradation, or mitochondrial import may play an important role in shaping BER capacity under metabolic stress. Moreover, the associations between selected mitochondrial BER proteins and the clinical parameters of glucose metabolism, lipid dysregulation, hepatic steatosis, and oxidative stress support the concept that leukocytes may reflect a systemic component of MASLD. Taken together, these results point to a model in which metabolic and oxidative burden, potentially driven by MASLD together with older age and an increased BMI, may influence BER regulation and mtDNA stability. Future investigations conducted in larger cohorts with carefully age- and BMI-matched controls and liver-based models will be required to validate this interpretation and further clarify the relationship between systemic metabolic dysfunction, BER dysregulation, and mtDNA instability in MASLD.

## Figures and Tables

**Table 1:** Baseline characteristics of MASLD patients and healthy controls.

Parameter	Controls (*n* = 30)	MASLD (*n* = 99)	*p*-Value
Age	26 (22–35)	66 (57.25–69.75)	<0.001
BMI. kg m^−2^	22.05 (20.07–23.66)	32.87 (28.78–36.41)	<0.001
Sex. n (%)			
Male	8 (26.7%)	47 (47.5%)	0.071
Female	22 (73.3%)	52 (52.5%)	

Data are presented as the median (IQR) or n (%). Statistical comparisons were performed using the Mann–Whitney U test and χ^2^ test, as appropriate. MASLD—metabolic dysfunction-associated steatotic liver disease.

**Table 2:** Clinical and biochemical features of patients with MASLD in the group of 99 individuals.

Parameters	Mean	SD
BMI, kg m^−2^	33.24	5.30
Fasting glucose, mg dL^−1^	129.85	24.91
Uric acid, mg dL^−1^	5.70	1.49
HbA1c, %	7.20	1.44
ALT, U L^−1^	35.18	21.58
AST, U L^−1^	29.70	14.07
HDL cholesterol, mg dL^−1^	52.94	15.94
LDL cholesterol, mg dL^−1^	101.71	38.91
TG, mg dL^−1^	173.61	94.59
GGT	59.98	103.12
HSI	45.96	7.00
FLI	85.52	12.87

ALT—alanine transaminase; AST—aspartate aminotransferase; BMI—body mass index; FLI—fatty liver index; GGT—gamma-glutamyl transferase; HDL—high-density lipoprotein; HSI—hepatic steatosis index; LDL—low-density lipoprotein; MASLD—metabolic dysfunction-associated steatotic liver disease; SD—standard deviation; TG—triglycerides.

**Table 3:** Probes chosen for the evaluation of gene expression.

Gene	Assay ID
*GAPDH*	Hs02786624_g1
*ACTB*	Hs01060665_g1
*18S*	Hs99999901_s1
*APEX1*	Hs00172396_m1
*NEIL1*	Hs00908563_m1
*POLG*	Hs00160298_m1
*LIG1*	Hs01553527_m1
*LIG3*	Hs00242692_m1
*XRCC1*	Hs00959834_m1
*PARP1*	Hs00242302_m1
*FEN1*	Hs00748727_s1
*OGG1*	Hs00213454_m1
*ENDOG*	Hs00172770_m1
*EXOG*	Hs01032857_m1

**Table 4:** Probes chosen for the evaluation of mitochondrial DNA copy number.

Gene	Protein	Assay ID
*mtND1*	NADH dehydrogenase 1	Hs02596873_s1-FAM
*mtND2*	NADH dehydrogenase 2	Hs02596874_g1-FAM
*mtCO1*	cytochrome c oxidase 1	Hs02596864_g1-FAM
*PKM*	pyruvate kinase	Hs00761782_s1-VIC

**Table 5:** PCR primers used for estimation of mtDNA and nDNA damage.

Gene	Forward Primer Sequences (5′→3′)	Reverse Primer Sequence (5′→3′)	Amplicon Length (bp)
*ND5*	Long fragment: TCCAACTCATGAGACCCACA	Long fragment: AGGTGATGATGGAGGTGGAG	1156
	Short fragment: AGGCGCTATCACCACTCTGT	Short fragment: TTGGTTGATGCCGATTGTAA	124
*HPRT1*	Long fragment: AGGGCAAAGGATGTGTTACG	Long fragment: AGTGGTTTCTGGTGCGACTT	1018
	Short fragment: TGGGAAAGGCAGATCTGGAG	Short fragment: GGGGTGTGGGAGGACATAAA	192

**Table 6:** Spearman’s correlation analyses of age and BMI with mitochondrial DNA maintenance-related parameters in patients with MASLD and the control groups.

Variable	Mitochondrial Parameter	MASLD Group r	MASLD Group *p*-Value	Control Group r	Control Group *p*-Value
Age	mtDNA copy number	0.04725	0.6638	−0.05011	0.8161
	mtDNA damage	−0.09379	0.3558	−0.2442	0.2614
	nDNA damage	−0.04355	0.6768	0.6110	0.0015
	NEIL1 mRNA level	0.2133	0.0720	−0.2018	0.3444
	APEX1 mRNA level	0.1626	0.1469	0.06902	0.7486
	POLG mRNA level	0.1517	0.1820	−0.3429	0.1010
	FEN1 mRNA level	0.06368	0.5771	−0.3722	0.0733
	PARP1 mRNA level	0.04530	0.6861	0.05583	0.7956
	XRCC1 mRNA level	0.1802	0.1169	−0.4959	0.0137
	LIG1 mRNA level	0.07923	0.5113	−0.2774	0.1894
	LIG3 mRNA level	0.1095	0.3431	−0.4452	0.0379
	OGG1 mRNA level	0.02842	0.8024	−0.002198	0.9919
	EXOG mRNA level	0.05274	0.6422	0.04352	0.8400
	ENDOG mRNA level	0.1385	0.2146	0.3191	0.1285
	NEIL1 protein	0.09010	0.7389	0.1542	0.5964
	APEX1 protein	0.2172	0.4337	−0.03222	0.9146
	POLG protein	−0.1965	0.4629	0.2693	0.3491
	FEN1 protein	−0.3516	0.1811	0.05983	0.8395
	PARP1 protein	−0.06056	0.8235	0.1496	0.6076
	XRCC1 protein	−0.4815	0.0607	0.1594	0.6014
	LIG1 protein	−0.1551	0.5636	0.1565	0.5908
	LIG3 protein	−0.1211	0.6531	0.1266	0.6647
	OGG1 protein	−0.5332	0.0354	0.4326	0.1230
	EXOG protein	−0.1670	0.5492	−0.07364	0.8025
	ENDOG protein	0.09749	0.7182	0.4948	0.0740
BMI	mtDNA copy number	−0.03484	0.7487	−0.3771	0.0399
	mtDNA damage	−0.06561	0.5188	−0.1443	0.4551
	nDNA damage	−0.09447	0.3651	0.4365	0.0159
	NEIL1 mRNA level	−0.07226	0.5464	0.2154	0.2530
	APEX1 mRNA level	−0.1154	0.3049	−0.05006	0.7928
	POLG mRNA level	0.1242	0.2755	−0.07988	0.6748
	FEN1 mRNA level	−0.01528	0.8937	−0.05207	0.7847
	PARP1 mRNA level	−0.1305	0.2425	−0.0006675	0.9972
	XRCC1 mRNA level	0.04330	0.7085	−0.07520	0.6929
	LIG1 mRNA level	0.1375	0.2529	−0.2750	0.1414
	LIG3 mRNA level	−0.04575	0.6928	−0.1284	0.5150
	OGG1 mRNA level	0.03390	0.7653	−0.1099	0.5631
	EXOG mRNA level	−0.02023	0.8586	−0.1609	0.3958
	ENDOG mRNA level	0.005605	0.9601	−0.02180	0.9089
	NEIL1 protein	−0.3382	0.2001	0.3618	0.1690
	APEX1 protein	0.4250	0.1159	−0.1147	0.6724
	POLG protein	0.4853	0.0589	0.05882	0.8308
	FEN1 protein	0.2176	0.4168	−0.02647	0.9258
	PARP1 protein	0.2559	0.3376	−0.06176	0.8222
	XRCC1 protein	0.1941	0.4701	−0.02857	0.9234
	LIG1 protein	0.4235	0.1036	−0.01471	0.9607
	LIG3 protein	−0.2765	0.2990	0.09706	0.7213
	OGG1 protein	0.4559	0.0779	0.08235	0.7630
	EXOG protein	−0.01786	0.9540	−0.3382	0.2001
	ENDOG protein	0.2206	0.4103	0.3294	0.2127

BMI—body mass index; MASLD—metabolic dysfunction-associated steatotic liver disease; mtDNA—mitochondrial DNA; nDNA—nuclear DNA.

**Table 7:** Multivariable linear regression analysis of molecular parameters associated with age or BMI, adjusted for group status.

Outcome	Predictor	β	95% CI	*p*-Value
nDNA damage	Age	0.00215	−0.00067 to 0.00497	0.134
	MASLD vs. control	0.1507	0.0387 to 0.2626	0.0088
XRCC1 mRNA level	Age	0.00116	−0.00134 to 0.00365	0.361
	MASLD vs. control	−0.01698	−0.1141 to 0.08014	0.730
LIG3 mRNA level	Age	−0.000480	−0.00187 to 0.000911	0.486
	MASLD vs. control	−0.00443	−0.0613 to 0.0525	0.875
OGG1 protein level	Age	−0.000575	−0.00180 to 0.000646	0.344
	MASLD vs. control	0.000191	−0.0497 to 0.0501	0.994
mtDNA copy number	BMI	−0.00164	−0.00908 to 0.00580	0.663
	MASLD vs. control	−0.0720	−0.1873 to 0.0432	0.218
nDNA damage	BMI	−0.00392	−0.00944 to 0.00160	0.162
	MASLD vs. control	0.2641	0.1777 to 0.3504	<0.0001

BMI—body mass index; CI—confidence interval; MASLD—metabolic dysfunction-associated steatotic liver disease; mtDNA—mitochondrial DNA; nDNA—nuclear DNA.

## Data Availability

The mass spectrometry proteomics data have been deposited in the ProteomeXchange Consortium via the PRIDE [82] partner repository with the dataset identifier PXD075974.

## Supplementary Materials

The following supporting information can be downloaded at: https://www.mdpi.com/article/10.3390/cells15151415/s1, Supplementary Data S1: The design process of primers for semi-long-run real-time PCR; Supplementary Data S2: List of stable isotope-labelled standard (SIS) peptides used for targeted PRM analysis. The table presents the target proteins and corresponding proteotypic peptide sequences selected for quantification. Please note that several proteins are referred to by alternative names or abbreviations in the main manuscript. The corresponding nomenclature used throughout the manuscript is provided in the table; Supplementary Data S3A: Non-significant Spearman’s correlations between clinical parameters and molecular markers analyzed in the study. Correlation coefficients and corresponding *p*-values are reported for all tested associations that did not reach statistical significance (*p* ≥ 0.05); Supplementary Data S3B: Correlations among molecular markers assessed in different experimental approaches (gene expression. mitochondrial protein levels. mtDNA copy number. and DNA damage parameters).

## Abbreviations

The following abbreviations are used in this manuscript:

ACTB	beta-actin
ALT	alanine aminotransferase
AP	abasic/apurinic-apyrimidinic site
AST	aspartate aminotransferase
BER	base excision repair
BiP	binding immunoglobulin protein
BMI	body mass index
cDNA	complementary DNA
CI	confidence interval
Ct	cycle threshold
EDTA	ethylenediaminetetraacetic acid
ER	endoplasmic reticulum
ETC	electron transport chain
FLI	fatty liver index
gDNA	genomic DNA
GGT	gamma-glutamyl transferase
HbA1c	glycated hemoglobin A1c
HCC	hepatocellular carcinoma
HDL	high-density lipoprotein
HFD	high-fat diet
HSI	hepatic steatosis index
IR	insulin resistance
LDL	low-density lipoprotein
LP-BER	long-patch base excision repair
MASLD	metabolic dysfunction-associated steatotic liver disease
MASH	metabolic dysfunction-associated steatohepatitis
MS	mass spectrometry
NAFLD	non-alcoholic fatty liver disease
nDNA	nuclear DNA
PBMCs	peripheral blood mononuclear cells
PBS	phosphate-buffered saline
PCR	polymerase chain reaction
PRIDE	Proteomics Identifications Database
PRM	parallel reaction monitoring
ROS	reactive oxygen species
ROUT	robust regression and outlier removal
SD	standard deviation
SIS	stable isotope-labeled standard
SNP	single nucleotide polymorphism
SP-BER	short-patch base excision repair
T2DM	type 2 diabetes mellitus
TG	triglycerides
USG	ultrasonography

## References

[1] Pouwels S., Sakran N., Graham Y., Leal A., Pintar T., Yang W., Kassir R., Singhal R., Mahawar K., Ramnarain D. (2022). Non-Alcoholic Fatty Liver Disease (NAFLD): A Review of Pathophysiology, Clinical Management and Effects of Weight Loss. BMC Endocr. Disord..

[2] Loomba R., Friedman S.L., Shulman G.I. (2021). Mechanisms and Disease Consequences of Nonalcoholic Fatty Liver Disease. Cell.

[3] Ziolkowska S., Binienda A., Jabłkowski M., Szemraj J., Czarny P. (2021). The Interplay between Insulin Resistance, Inflammation, Oxidative Stress, Base Excision Repair and Metabolic Syndrome in Nonalcoholic Fatty Liver Disease. Int. J. Mol. Sci..

[4] Liang D., Burkhart S.L., Singh R.K., Kabbaj M.H.M., Gunjan A. (2012). Histone Dosage Regulates DNA Damage Sensitivity in a Checkpoint-Independent Manner by the Homologous Recombination Pathway. Nucleic Acids Res..

[5] Lim K.S., Jeyaseelan K., Whiteman M., Jenner A., Halliwell B. (2005). Oxidative Damage in Mitochondrial DNA Is Not Extensive. Ann. N. Y. Acad. Sci..

[6] Gohil D., Sarker A.H., Roy R. (2023). Base Excision Repair: Mechanisms and Impact in Biology, Disease, and Medicine. Int. J. Mol. Sci..

[7] Hsiao P.-J., Hsieh T.-J., Kuo K.-K., Hung W.-W., Tsai K.-B., Yang C.-H., Yu M.-L., Shin S.-J. (2008). Pioglitazone Retrieves Hepatic Antioxidant DNA Repair in a Mice Model of High Fat Diet. BMC Mol. Biol..

[8] Takumi S., Okamura K., Yanagisawa H., Sano T., Kobayashi Y., Nohara K. (2015). The Effect of a Methyl-Deficient Diet on the Global DNA Methylation and the DNA Methylation Regulatory Pathways. J. Appl. Toxicol..

[9] Gao D., Wei C., Chen L., Huang J., Yang S., Diehl A.M. (2004). Oxidative DNA Damage and DNA Repair Enzyme Expression Are Inversely Related in Murine Models of Fatty Liver Disease. Am. J. Physiol. Gastrointest. Liver Physiol..

[10] Kosmalski M., Frankowski R., Ziółkowska S., Różycka-Kosmalska M., Pietras T. (2023). What’s New in the Treatment of Non-Alcoholic Fatty Liver Disease (NAFLD). J. Clin. Med..

[11] Panera N., Braghini M.R., Crudele A., Smeriglio A., Bianchi M., Condorelli A.G., Nobili R., Conti L.A., De Stefanis C., Lioci G. (2022). Combination Treatment with Hydroxytyrosol and Vitamin E Improves NAFLD-Related Fibrosis. Nutrients.

[12] Takahashi H., Kessoku T., Kawanaka M., Nonaka M., Hyogo H., Fujii H., Nakajima T., Imajo K., Tanaka K., Kubotsu Y. (2022). Ipragliflozin Improves the Hepatic Outcomes of Patients with Diabetes with NAFLD. Hepatol. Commun..

[13] Taheri H., Malek M., Ismail-Beigi F., Zamani F., Sohrabi M., Reza Babaei M., Khamseh M.E. (2020). Effect of Empagliflozin on Liver Steatosis and Fibrosis in Patients with Non-Alcoholic Fatty Liver Disease Without Diabetes: A Randomized, Double-Blind, Placebo-Controlled Trial. Adv. Ther..

[14] Harrison S.A., Bedossa P., Guy C.D., Schattenberg J.M., Loomba R., Taub R., Labriola D., Moussa S.E., Neff G.W., Rinella M.E. (2024). A Phase 3, Randomized, Controlled Trial of Resmetirom in NASH with Liver Fibrosis. N. Engl. J. Med..

[15] Della Pepa G., Russo M., Vitale M., Carli F., Vetrani C., Masulli M., Riccardi G., Vaccaro O., Gastaldelli A., Rivellese A.A. (2021). Pioglitazone Even at Low Dosage Improves NAFLD in Type 2 Diabetes: Clinical and Pathophysiological Insights from a Subgroup of the TOSCA.IT Randomised Trial. Diabetes Res. Clin. Pract..

[16] Newsome P.N., Buchholtz K., Cusi K., Linder M., Okanoue T., Ratziu V., Sanyal A.J., Sejling A.-S., Harrison S.A. (2021). A Placebo-Controlled Trial of Subcutaneous Semaglutide in Nonalcoholic Steatohepatitis. N. Engl. J. Med..

[17] Pfaffl M.W., Tichopad A., Prgomet C., Neuvians T.P. (2004). Determination of Stable Housekeeping Genes, Differentially Regulated Target Genes and Sample Integrity: BestKeeper--Excel-Based Tool Using Pair-Wise Correlations. Biotechnol. Lett..

[18] Mori K.M., McElroy J.P., Weng D.Y., Chung S., Fadda P., Reisinger S.A., Ying K.L., Brasky T.M., Wewers M.D., Freudenheim J.L. (2022). Lung Mitochondrial DNA Copy Number, Inflammatory Biomarkers, Gene Transcription and Gene Methylation in Vapers and Smokers. eBioMedicine.

[19] Rothfuss O., Gasser T., Patenge N. (2010). Analysis of Differential DNA Damage in the Mitochondrial Genome Employing a Semi-Long Run Real-Time PCR Approach. Nucleic Acids Res..

[20] Czarny P., Wigner P., Strycharz J., Swiderska E., Synowiec E., Szatkowska M., Sliwinska A., Talarowska M., Szemraj J., Su K.P. (2020). Mitochondrial DNA Copy Number, Damage, Repair and Degradation in Depressive Disorder. World J. Biol. Psychiatry.

[21] Santos J.H., Mandavilli B.S., Van Houten B. (2002). Measuring Oxidative MtDNA Damage and Repair Using Quantitative PCR. Methods Mol. Biol..

[22] Hughes C.S., Moggridge S., Müller T., Sorensen P.H., Morin G.B., Krijgsveld J. (2018). Single-Pot, Solid-Phase-Enhanced Sample Preparation for Proteomics Experiments. Nat. Protoc..

[23] Shirakawa R., Nakajima T., Yoshimura A., Kawahara Y., Orito C., Yamane M., Handa H., Takada S., Furihata T., Fukushima A. (2023). Enhanced Mitochondrial Oxidative Metabolism in Peripheral Blood Mononuclear Cells Is Associated with Fatty Liver in Obese Young Adults. Sci. Rep..

[24] Pirola C.J., Garaycoechea M., Flichman D., Castaño G.O., Sookoian S. (2021). Liver Mitochondrial DNA Damage and Genetic Variability of Cytochrome b—A Key Component of the Respirasome—Drive the Severity of Fatty Liver Disease. J. Intern. Med..

[25] Nishida N., Yada N., Hagiwara S., Sakurai T., Kitano M., Kudo M. (2016). Unique Features Associated with Hepatic Oxidative DNA Damage and DNA Methylation in Non-Alcoholic Fatty Liver Disease. J. Gastroenterol. Hepatol..

[26] Lee A.H., Oh J.H., Kim H.S., Shin J.H., Yoon E.L., Jun D.W. (2022). Peripheral Blood Mononuclear Cell Mitochondrial Copy Number and Adenosine Triphosphate Inhibition Test in NAFLD. Front. Endocrinol..

[27] Kamfar S., Alavian S.M., Houshmand M., Yadegarazari R., Zarei B.S., Khalaj A., Shabab N., Saidijam M. (2016). Liver Mitochondrial DNA Copy Number and Deletion Levels May Contribute to Nonalcoholic Fatty Liver Disease Susceptibility. Hepat. Mon..

[28] Houshmand M., Zeinali V., Hosseini A., Seifi A., Danaei B., Kamfar S. (2023). Investigation of FGF21 MRNA Levels and Relative Mitochondrial DNA Copy Number Levels and Their Relation in Nonalcoholic Fatty Liver Disease: A Case-Control Study. Front. Mol. Biosci..

[29] Malik A.N., Simões I.C.M., Rosa H.S., Khan S., Karkucinska-Wieckowska A., Wieckowski M.R. (2019). A Diet Induced Maladaptive Increase in Hepatic Mitochondrial DNA Precedes OXPHOS Defects and May Contribute to Non-Alcoholic Fatty Liver Disease. Cells.

[30] Alexeyev M., Shokolenko I., Wilson G., LeDoux S. (2013). The Maintenance of Mitochondrial DNA Integrity—Critical Analysis and Update. Cold Spring Harb. Perspect. Biol..

[31] Rong Z., Tu P., Xu P., Sun Y., Yu F., Tu N., Guo L., Yang Y. (2021). The Mitochondrial Response to DNA Damage. Front. Cell Dev. Biol..

[32] Ziółkowska S., Kosmalski M., Kołodziej Ł., Jabłkowska A., Szemraj J.Z., Pietras T., Jabłkowski M., Czarny P.L. (2023). Single-Nucleotide Polymorphisms in Base-Excision Repair-Related Genes Involved in the Risk of an Occurrence of Non-Alcoholic Fatty Liver Disease. Int. J. Mol. Sci..

[33] Minko I.G., Luzadder M.M., Vartanian V.L., Rice S.P.M., Nguyen M.M., Sanchez-Contreras M., Van P., Kennedy S.R., McCullough A.K., Lloyd R.S. (2024). Frequencies and Spectra of Aflatoxin B1-Induced Mutations in Liver Genomes of NEIL1-Deficient Mice as Revealed by Duplex Sequencing. NAR Mol. Med..

[34] Vartanian V., Lowell B., Minko I.G., Wood T.G., Ceci J.D., George S., Ballinger S.W., Corless C.L., McCullough A.K., Lloyd R.S. (2006). The Metabolic Syndrome Resulting from a Knockout of the NEIL1 DNA Glycosylase. Proc. Natl. Acad. Sci. USA.

[35] Tsuboi T., Leff J., Zid B.M. (2020). Post-Transcriptional Control of Mitochondrial Protein Composition in Changing Environmental Conditions. Biochem. Soc. Trans..

[36] Christmann M., Kaina B. (2013). Transcriptional Regulation of Human DNA Repair Genes Following Genotoxic Stress: Trigger Mechanisms, Inducible Responses and Genotoxic Adaptation. Nucleic Acids Res..

[37] Sampath H., Vartanian V., Rollins M.R., Sakumi K., Nakabeppu Y., Lloyd R.S. (2012). 8-Oxoguanine DNA Glycosylase (OGG1) Deficiency Increases Susceptibility to Obesity and Metabolic Dysfunction. PLoS ONE.

[38] Yuzefovych L.V., Noh H.L., Suk S., Schuler A.M., Mulekar M.S., Pastukh V.M., Kim J.K., Rachek L.I. (2024). Mitochondria-Targeted DNA Repair Glycosylase HOGG1 Protects Against HFD-Induced Liver Oxidative Mitochondrial DNA Damage and Insulin Resistance in OGG1-Deficient Mice. Int. J. Mol. Sci..

[39] Komakula S.S.B., Blaze B., Ye H., Dobrzyn A., Sampath H. (2021). A Novel Role for the DNA Repair Enzyme 8-Oxoguanine DNA Glycosylase in Adipogenesis. Int. J. Mol. Sci..

[40] Komakula S.S.B., Tumova J., Kumaraswamy D., Burchat N., Vartanian V., Ye H., Dobrzyn A., Lloyd R.S., Sampath H. (2018). The DNA Repair Protein OGG1 Protects Against Obesity by Altering Mitochondrial Energetics in White Adipose Tissue. Sci. Rep..

[41] Tu H., Willems I., Mircheva A., Bastos V.C., van den Beucken T., Dubois L., Remels A.H.V., van Schooten F.J., Godschalk R.W.L., Langie S.A.S. (2025). The Role of DNA Repair Deficiency in Lipid Accumulation: A Proof-of-Concept Study. DNA Repair.

[42] Eckl E.M., Ziegemann O., Krumwiede L., Fessler E., Jae L.T. (2021). Sensing, Signaling and Surviving Mitochondrial Stress. Cell. Mol. Life Sci..

[43] Opalińska M., Meisinger C. (2014). Mitochondrial Protein Import under Kinase Surveillance. Microb. Cell.

[44] Dianov G.L., Hübscher U. (2013). Mammalian Base Excision Repair: The Forgotten Archangel. Nucleic Acids Res..

[45] Lu X., Zhao H., Yuan H., Chu Y., Zhu X. (2021). High Nuclear Expression of APE1 Correlates with Unfavorable Prognosis and Promotes Tumor Growth in Hepatocellular Carcinoma. J. Mol. Histol..

[46] Huang K., Du M., Tan X., Yang L., Li X., Jiang Y., Wang C., Zhang F., Zhu F., Cheng M. (2017). PARP1-Mediated PPARα Poly(ADP-Ribosyl)Ation Suppresses Fatty Acid Oxidation in Non-Alcoholic Fatty Liver Disease. J. Hepatol..

[47] Zai W., Chen W., Han Y., Wu Z., Fan J., Zhang X., Luan J., Tang S., Jin X., Fu X. (2020). Targeting PARP and Autophagy Evoked Synergistic Lethality in Hepatocellular Carcinoma. Carcinogenesis.

[48] Szántó M., Gupte R., Kraus W.L., Pacher P., Bai P. (2021). PARPs in Lipid Metabolism and Related Diseases. Prog. Lipid Res..

[49] Bai P., Cantó C., Oudart H., Brunyánszki A., Cen Y., Thomas C., Yamamoto H., Huber A., Kiss B., Houtkooper R.H. (2011). PARP-1 Inhibition Increases Mitochondrial Metabolism through SIRT1 Activation. Cell Metab..

[50] Mukhopadhyay P., Rajesh M., Cao Z., Horváth B., Park O., Wang H., Erdelyi K., Holovac E., Wang Y., Liaudet L. (2014). Poly (ADP-Ribose) Polymerase-1 Is a Key Mediator of Liver Inflammation and Fibrosis. Hepatology.

[51] Mukhopadhyay P., Horváth B., Rajesh M., Varga Z.V., Gariani K., Ryu D., Cao Z., Holovac E., Park O., Zhou Z. (2017). PARP Inhibition Protects against Alcoholic and Non-Alcoholic Steatohepatitis. J. Hepatol..

[52] Thakur S., Sarkar B., Cholia R.P., Gautam N., Dhiman M., Mantha A.K. (2014). APE1/Ref-1 as an Emerging Therapeutic Target for Various Human Diseases: Phytochemical Modulation of Its Functions. Exp. Mol. Med..

[53] Prakash A., Doublié S. (2015). Base Excision Repair in the Mitochondria. J. Cell. Biochem..

[54] Squires J.E., Miethke A.G., Alexander Valencia C., Hawthorne K., Henn L., Van Hove J.L.K., Squires R.H., Bove K., Horslen S., Kohli R. (2023). Clinical Spectrum and Genetic Causes of Mitochondrial Hepatopathy Phenotype in Children. Hepatol. Commun..

[55] Kristensen E., Naess K., Engvall M., Klingenberg C., Rasmussen M., Brodtkorb E., Ostergaard E., de Coo I., Pias-Peleteiro L., Isohanni P. (2025). Liver Involvement in POLG Disease—A Multicentre Cohort Study of 202 Patients. J. Inherit. Metab. Dis..

[56] Rötig A., Gaignard P., Barcia G., Assouline Z., Berat C.M., Barth M., Damaj L., Laborde N., Abi-Warde M.T., Chabrol B. (2024). Distinct Clinical Courses and Shortened Lifespans in Childhood-Onset DNA Polymerase Gamma Deficiency. Neurol. Genet..

[57] Anagnostou M.E., Ng Y.S., Taylor R.W., McFarland R. (2016). Epilepsy Due to Mutations in the Mitochondrial Polymerase Gamma (POLG) Gene: A Clinical and Molecular Genetic Review. Epilepsia.

[58] Kucherlapati M., Yang K., Kuraguchi M., Zhao J., Lia M., Heyer J., Kane M.F., Fan K., Russell R., Brown A.M.C. (2002). Haploinsufficiency of Flap Endonuclease (Fen1) Leads to Rapid Tumor Progression. Proc. Natl. Acad. Sci. USA.

[59] Larsen E., Gran C., Sæther B.E., Seeberg E., Klungland A. (2003). Proliferation Failure and Gamma Radiation Sensitivity of Fen1 Null Mutant Mice at the Blastocyst Stage. Mol. Cell. Biol..

[60] Lam J.S., Seligson D.B., Yu H., Li A., Eeva M., Pantuck A.J., Zeng G., Horvath S., Belldegrun A.S. (2006). Flap Endonuclease 1 Is Overexpressed in Prostate Cancer and Is Associated with a High Gleason Score. BJU Int..

[61] Mortusewicz O., Rothbauer U., Cardoso M.C., Leonhardt H. (2006). Differential Recruitment of DNA Ligase I and III to DNA Repair Sites. Nucleic Acids Res..

[62] Han M., Piorońska W., Wang S., Nwosu Z.C., Sticht C., Wang S., Gao Y., Ebert M.P., Dooley S., Meyer C. (2020). Hepatocyte Caveolin-1 Modulates Metabolic Gene Profiles and Functions in Non-Alcoholic Fatty Liver Disease. Cell Death Dis..

[63] Bentley D.J., Selfridge J., Millar J.K., Samuel K., Hole N., Ansell J.D., Melton D.W. (1996). DNA Ligase I Is Required for Fetal Liver Erythropoiesis but Is Not Essential for Mammalian Cell Viability. Nat. Genet..

[64] Al-Qadhi Z.T.A., Babaresh W.M.A., Rehman I.U., Hassen R.S.A., Allah A., Ali S.A., Al-Mansoub H., Al-Samai F., Retes Y.N. (2025). Genotype and Association of XRCC1 Gene with Liver Disease in Chronic Hcv Patients. Asian Pac. J. Cancer Prev..

[65] Ghorab R.A., Fouad S.H., Elsaadawy Y., Hamdy M., Taha S.I. (2024). Association of XRCC1 p. Arg194Trp Gene Polymorphism with the Risk of Hepatocellular Carcinoma in HCV Egyptian Population: A Pilot Case-Control Study. Int. J. Immunopathol. Pharmacol..

[66] Saad A.M., Abdel-Megied A.E.S., Elbaz R.A., Hassab El-Nabi S.E., Elshazli R.M. (2021). Genetic Variants of APEX1 p.Asp148Glu and XRCC1 p.Gln399Arg with the Susceptibility of Hepatocellular Carcinoma. J. Med. Virol..

[67] Hassen R.S.A., Babaresh W.M., Ur Rehman I., Talal Z., Nayyar M., Rettas Y., Shakeel S.J., Huriya S., Begum A., Khan O. (2025). Association of XRCC1 Gene Polymorphism with an Increased Risk of Hepatocellular Carcinoma in the Peshawar Population. Asian Pac. J. Cancer Prev..

[68] Mei J., Wang H., Wang R., Pan J., Liu C., Xu J. (2020). Evaluation of X-Ray Repair Cross-Complementing Family Members as Potential Biomarkers for Predicting Progression and Prognosis in Hepatocellular Carcinoma. BioMed Res. Int..

[69] Krupa R., Czarny P., Wigner P., Wozny J., Jablkowski M., Kordek R., Szemraj J., Sliwinski T. (2017). The Relationship Between Single-Nucleotide Polymorphisms, the Expression of DNA Damage Response Genes, and Hepatocellular Carcinoma in a Polish Population. DNA Cell Biol..

[70] Wang W., Li J., Tan J., Wang M., Yang J., Zhang Z.M., Li C., Basnakian A.G., Tang H.W., Perrimon N. (2021). Endonuclease G Promotes Autophagy by Suppressing MTOR Signaling and Activating the DNA Damage Response. Nat. Commun..

[71] Wang W., Tan J., Liu X., Guo W., Li M., Liu X., Liu Y., Dai W., Hu L., Wang Y. (2023). Cytoplasmic Endonuclease G Promotes Nonalcoholic Fatty Liver Disease via MTORC2-AKT-ACLY and Endoplasmic Reticulum Stress. Nat. Commun..

[72] Xu X., Penjweini R., Székvölgyi L., Karányi Z., Heckel A.M., Gurusamy D., Varga D., Yang S., Brown A.L., Cui W. (2025). Endonuclease G Promotes Hepatic Mitochondrial Respiration by Selectively Increasing Mitochondrial TRNAThr Production. Proc. Natl. Acad. Sci. USA.

[73] Szymanski M.R., Yu W., Gmyrek A.M., White M.A., Molineux I.J., Lee J.C., Yin Y.W. (2017). A Domain in Human EXOG Converts Apoptotic Endonuclease to DNA-Repair Exonuclease. Nat. Commun..

[74] Szymanski M.R., Karlowicz A., Herrmann G.K., Cen Y., Yin Y.W. (2022). Human EXOG Possesses Strong AP Hydrolysis Activity: Implication on Mitochondrial DNA Base Excision Repair. J. Am. Chem. Soc..

[75] Karlowicz A., Dubiel A.B., Wyszkowska M., Hossain K.A., Czub J., Szymanski M.R. (2025). Mitochondrial Exonuclease EXOG Supports DNA Integrity by the Removal of Single-Stranded DNA Flaps. Nucleic Acids Res..

[76] Parrinello C.M., Selvin E. (2014). Beyond HbA1c and Glucose: The Role of Nontraditional Glycemic Markers in Diabetes Diagnosis, Prognosis, and Management. Curr. Diab. Rep..

[77] Ahn S.B. (2023). Noninvasive Serum Biomarkers for Liver Steatosis in Nonalcoholic Fatty Liver Disease: Current and Future Developments. Clin. Mol. Hepatol..

[78] Gherghina M.E., Peride I., Tiglis M., Neagu T.P., Niculae A., Checherita I.A. (2022). Uric Acid and Oxidative Stress—Relationship with Cardiovascular, Metabolic, and Renal Impairment. Int. J. Mol. Sci..

[79] Huang J., Zhang C., Huang C., Deng K., Xiao Y., Gao W., Wu M., Lei M. (2025). Mitochondria Metabolism Regulates Glucose–Lipid Homeostasis in Neurodegenerative Diseases. Research.

[80] Miller D.M., McCauley K.F., Dunham-Snary K.J. (2025). Metabolic Dysfunction-Associated Steatotic Liver Disease (MASLD): Mechanisms, Clinical Implications and Therapeutic Advances. Endocrinol. Diabetes Metab..

[81] Scarpulla R.C. (2011). Metabolic Control of Mitochondrial Biogenesis through the PGC-1 Family Regulatory Network. Biochim. Biophys. Acta BBA Mol. Cell Res..

[82] Perez-Riverol Y., Bandla C., Kundu D.J., Kamatchinathan S., Bai J., Hewapathirana S., John N.S., Prakash A., Walzer M., Wang S. (2024). The PRIDE Database at 20 Years: 2025 Update. Nucleic Acids Res..

