# Supplementary material for: Altered Mitochondrial Base Excision Repair and Mitochondrial DNA Instability in Peripheral Leukocytes of Patients with MASLD
Source: Cells. 2026 Aug 5;15(15):1415. doi: 10.3390/cells15151415 (PMC13463988; doi:10.3390/cells15151415)
Supplement: Supplementary file 1 [file cells-15-01415-s001.zip › Supplementary Data S1.pdf]

Supplementary Data S1: The design process of primers for semi-long-run real-time PCR.

ND5 gene

***ND5* gene sequence from Ensembl genome browser 95:**
https://www.ensembl.org/Homo_sapiens/Gene/Sequence?db=core;g=ENSG0000019878
6;r=MT:12337-14148;t=ENST00000361567

```
   1 ATAACCATGCACACTACTATAACCACCCTAACCCTGACTTCCCTAATTCCCCCCATCCTT
  61 ACCACCCTCGTTAACCCTAACAAAAAAAACTCATACCCCCATTATGTAAAATCCATTGTC
 121 GCATCCACCTTTATTATCAGTCTCTTCCCCACAACAATATTCATGTGCCTAGACCAAGAA
 181 GTTATTATCTCGAACTGACACTGAGCCACAACCCAAACAACCCAGCTCTCCCTAAGCTTC
 241 AAACTAGACTACTTCTCCATAATATTCATCCCTGTAGCATTGTTCGTTACATGGTCCATC
 301 ATAGAATTCTCACTGTGATATATAAACTCAGACCCAAACATTAATCAGTTCTTCAAATAT
 361 CTACTCATCTTCCTAATTACCATACTAATCTTAGTTACCGCTAACAACCTATTCCAACTG
 421 TTCATCGGCTGAGAGGGCGTAGGAATTATATCCTTCTTGCTCATCAGTTGATGATACGCC
 481 CGAGCAGATGCCAACACAGCAGCCATTCAAGCAATCCTATACAACCGTATCGGCGATATC
 541 GGTTTCATCCTCGCCTTAGCATGATTTATCCTACACTCCAACTCATGAGACCCACAACAA
 601 ATAGCCCTTCTAAACGCTAATCCAAGCCTCACCCCACTACTAGGCCTCCTCCTAGCAGCA
 661 GCAGGCAAATCAGCCCAATTAGGTCTCCACCCCTGACTCCCCTCAGCCATAGAAGGCCCC
 721 ACCCCAGTCTCAGCCCTACTCCACTCAAGCACTATAGTTGTAGCAGGAATCTTCTTACTC
 781 ATCCGCTTCCACCCCCTAGCAGAAAATAGCCCACTAATCCAAACTCTAACACTATGCTTA
 841 GGCGCTATCACCACTCTGTTCGCAGCAGTCTGCGCCCTTACACAAAATGACATCAAAAAA
 901 ATCGTAGCCTTCTCCACTTCAAGTCAACTAGGACTCATAATAGTTACAATCGGCATCAAC
 961 CAACCACACCTAGCATTCCTGCACATCTGTACCCACGCCTTCTTCAAAGCCATACTATTT
1021 ATGTGCTCCGGGTCCATCATCCACAACCTTAACAATGAACAAGATATTCGAAAAATAGGA
1081 GGACTACTCAAAACCATACCTCTCACTTCAACCTCCCTCACCATTGGCAGCCTAGCATTA
1141 GCAGGAATACCTTTCCTCACAGGTTTCTACTCCAAAGACCACATCATCGAAACCGCAAAC
1201 ATATCATACACAAACGCCTGAGCCCTATCTATTACTCTCATCGCTACCTCCCTGACAAGC
1261 GCCTATAGCACTCGAATAATTCTTCTCACCCTAACAGGTCAACCTCGCTTCCCCACCCTT
1321 ACTAACATTAACGAAAATAACCCCACCCTACTAAACCCCATTAAACGCCTGGCAGCCGGA
1381 AGCCTATTCGCAGGATTTCTCATTACTAACAACATTTCCCCCGCATCCCCCTTCCAAACA
1441 ACAATCCCCCTCTACCTAAAACTCACAGCCCTCGCTGTCACTTTCCTAGGACTTCTAACA
1501 GCCCTAGACCTCAACTACCTAACCAACAAACTTAAAATAAAATCCCCACTATGCACATTT
1561 TATTTCTCCAACATACTCGGATTCTACCCTAGCATCACACACCGCACAATCCCCTATCTA
1621 GGCCTTCTTACGAGCCAAAACCTGCCCCTACTCCTCCTAGACCTAACCTGACTAGAAAAG
1681 CTATTACCTAAAACAATTTCACAGCACCAAATCTCCACCTCCATCATCACCTCAACCCAA
1741 AAAGGCATAATTAAACTTTACTTCCTCTCTTTCTTCTTCCCACTCATCCTAACCCTACTC
1801 CTAATCACATAA
```

Long fragment:

Primer 3 – primers design

http://bioinfo.ut.ee/primer3-0.4.0/

```
No mispriming library specified
Using 1-based sequence positions
OLIGO            start  len     tm     gc%   any    3' seq
LEFT PRIMER        577   20  60.09   50.00  8.00  1.00
TCCAACTCATGAGACCCACA
RIGHT PRIMER      1732   20  59.92   55.00  3.00  0.00
AGGTGATGATGGAGGTGGAG
SEQUENCE SIZE: 1812
INCLUDED REGION SIZE: 1812
```

```
PRODUCT SIZE: 1156, PAIR ANY COMPL: 6.00, PAIR 3' COMPL: 2.00
TARGETS (start, len)*: 869,3

    1 ATAACCATGCACACTACTATAACCACCCTAACCCTGACTTCCCTAATTCCCCCCATCCTT

   61 ACCACCCTCGTTAACCCTAACAAAAAAAACTCATACCCCCATTATGTAAAATCCATTGTC

  121 GCATCCACCTTTATTATCAGTCTCTTCCCCACAACAATATTCATGTGCCTAGACCAAGAA

  181 GTTATTATCTCGAACTGACACTGAGCCACAACCCAAACAACCCAGCTCTCCCTAAGCTTC

  241 AAACTAGACTACTTCTCCATAATATTCATCCCTGTAGCATTGTTCGTTACATGGTCCATC

  301 ATAGAATTCTCACTGTGATATATAAACTCAGACCCAAACATTAATCAGTTCTTCAAATAT

  361 CTACTCATCTTCCTAATTACCATACTAATCTTAGTTACCGCTAACAACCTATTCCAACTG

  421 TTCATCGGCTGAGAGGGCGTAGGAATTATATCCTTCTTGCTCATCAGTTGATGATACGCC

  481 CGAGCAGATGCCAACACAGCAGCCATTCAAGCAATCCTATACAACCGTATCGGCGATATC

  541 GGTTTCATCCTCGCCTTAGCATGATTTATCCTACACTCCAACTCATGAGACCCACAACAA
                                          >>>>>>>>>>>>>>>>>>>>
  601 ATAGCCCTTCTAAACGCTAATCCAAGCCTCACCCCACTACTAGGCCTCCTCCTAGCAGCA

  661 GCAGGCAAATCAGCCCAATTAGGTCTCCACCCCTGACTCCCCTCAGCCATAGAAGGCCCC

  721 ACCCCAGTCTCAGCCCTACTCCACTCAAGCACTATAGTTGTAGCAGGAATCTTCTTACTC

  781 ATCCGCTTCCACCCCCTAGCAGAAAATAGCCCACTAATCCAAACTCTAACACTATGCTTA

  841 GGCGCTATCACCACTCTGTTCGCAGCAGTCTGCGCCCTTACACAAAATGACATCAAAAAA
                                  ***
  901 ATCGTAGCCTTCTCCACTTCAAGTCAACTAGGACTCATAATAGTTACAATCGGCATCAAC

  961 CAACCACACCTAGCATTCCTGCACATCTGTACCCACGCCTTCTTCAAAGCCATACTATTT

 1021 ATGTGCTCCGGGTCCATCATCCACAACCTTAACAATGAACAAGATATTCGAAAAATAGGA

 1081 GGACTACTCAAAACCATACCTCTCACTTCAACCTCCCTCACCATTGGCAGCCTAGCATTA

 1141 GCAGGAATACCTTTCCTCACAGGTTTCTACTCCAAAGACCACATCATCGAAACCGCAAAC
```

```
 1201 ATATCATACACAAACGCCTGAGCCCTATCTATTACTCTCATCGCTACCTCCCTGACAAGC

 1261 GCCTATAGCACTCGAATAATTCTTCTCACCCTAACAGGTCAACCTCGCTTCCCCACCCTT

 1321 ACTAACATTAACGAAAATAACCCCACCCTACTAAACCCCATTAAACGCCTGGCAGCCGGA

 1381 AGCCTATTCGCAGGATTTCTCATTACTAACAACATTTCCCCCGCATCCCCCTTCCAAACA

 1441 ACAATCCCCCTCTACCTAAAACTCACAGCCCTCGCTGTCACTTTCCTAGGACTTCTAACA

 1501 GCCCTAGACCTCAACTACCTAACCAACAAACTTAAAATAAAATCCCCACTATGCACATTT

 1561 TATTTCTCCAACATACTCGGATTCTACCCTAGCATCACACACCGCACAATCCCCTATCTA

 1621 GGCCTTCTTACGAGCCAAAACCTGCCCCTACTCCTCCTAGACCTAACCTGACTAGAAAAG

 1681 CTATTACCTAAAACAATTTCACAGCACCAAATCTCCACCTCCATCATCACCTCAACCCAA
                                      <<<<<<<<<<<<<<<<<<<<
 1741 AAAGGCATAATTAAACTTTACTTCCTCTCTTTCTTCTTCCCACTCATCCTAACCCTACTC

 1801 CTAATCACATAA


KEYS (in order of precedence):
****** target
>>>>>> left primer
<<<<<< right primer


(primer3_results.cgi release 0.4.0)
```

Primer-BLAST - Detailed primer reports
https://www.ncbi.nlm.nih.gov/tools/primer-blast/

Primer pair 1

	Sequence (5'->3')	Length	Tm	GC%	Self complementarity
**Forward primer**	TCCAACTCATGAGACCCACA	20	58.27	50.00	8.00
**Reverse primer**	AGGTGATGATGGAGGTGGAG	20	58.50	55.00	3.00

Products on target templates

>NC_012920.1 Homo sapiens mitochondrion, complete genome

```
product length = 1156
Forward primer  1      TCCAACTCATGAGACCCACA  20
Template        12913  ....................  12932
```

```
Reverse primer  1       AGGTGATGATGGAGGTGGAG  20
Template        14068   ....................  14049
```

PCR Optimization

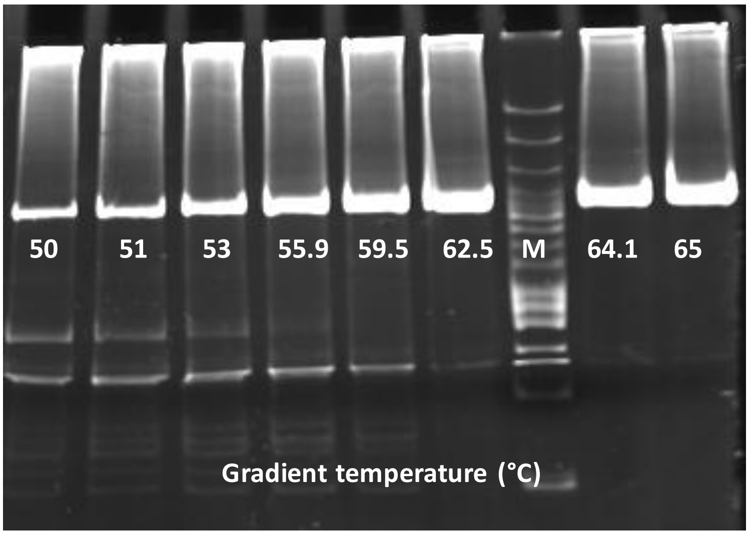

Gradient optimization of a PCR assay for *ND5* gene (long fragment). Gel photo showing PCR products formed at different annealing temperatures in a gradient optimization.

Real-time PCR Optimization

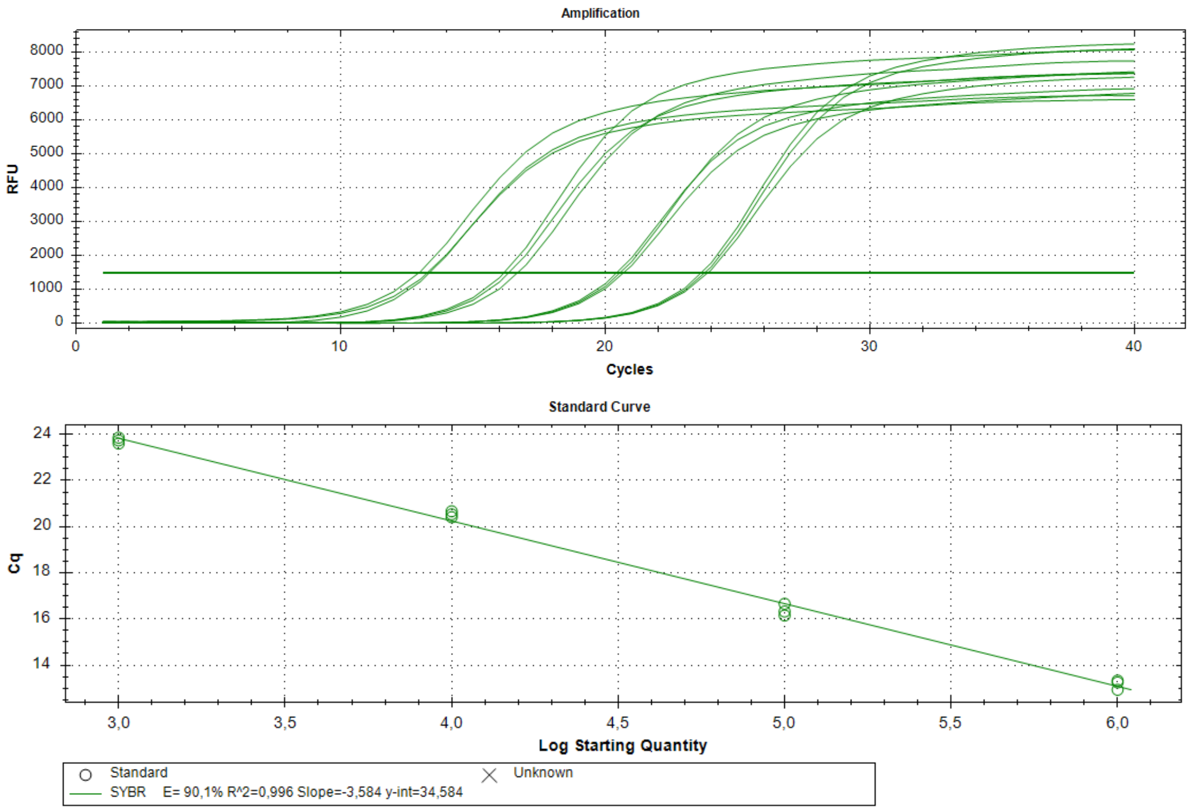

Standard curve with diluted sample: 1/1; 1/10; 1/100; 1/1000.

Small fragment:

Primer 3 - primers design

http://bioinfo.ut.ee/primer3-0.4.0/

```
No mispriming library specified
Using 1-based sequence positions
OLIGO            start  len      tm     gc%   any    3' seq
LEFT PRIMER        840   20   59.90   55.00  4.00  1.00
AGGCGCTATCACCACTCTGT
RIGHT PRIMER       963   20   59.93   40.00  2.00  2.00
TTGGTTGATGCCGATTGTAA
SEQUENCE SIZE: 1812
INCLUDED REGION SIZE: 1812

PRODUCT SIZE: 124, PAIR ANY COMPL: 5.00, PAIR 3' COMPL: 1.00
TARGETS (start, len)*: 869,3

    1 ATAACCATGCACACTACTATAACCACCCTAACCCTGACTTCCCTAATTCCCCCCATCCTT

   61 ACCACCCTCGTTAACCCTAACAAAAAAAACTCATACCCCCATTATGTAAAATCCATTGTC

  121 GCATCCACCTTTATTATCAGTCTCTTCCCCACAACAATATTCATGTGCCTAGACCAAGAA

  181 GTTATTATCTCGAACTGACACTGAGCCACAACCCAAACAACCCAGCTCTCCCTAAGCTTC

  241 AAACTAGACTACTTCTCCATAATATTCATCCCTGTAGCATTGTTCGTTACATGGTCCATC

  301 ATAGAATTCTCACTGTGATATATAAACTCAGACCCAAACATTAATCAGTTCTTCAAATAT

  361 CTACTCATCTTCCTAATTACCATACTAATCTTAGTTACCGCTAACAACCTATTCCAACTG

  421 TTCATCGGCTGAGAGGGCGTAGGAATTATATCCTTCTTGCTCATCAGTTGATGATACGCC

  481 CGAGCAGATGCCAACACAGCAGCCATTCAAGCAATCCTATACAACCGTATCGGCGATATC

  541 GGTTTCATCCTCGCCTTAGCATGATTTATCCTACACTCCAACTCATGAGACCCACAACAA

  601 ATAGCCCTTCTAAACGCTAATCCAAGCCTCACCCCACTACTAGGCCTCCTCCTAGCAGCA

  661 GCAGGCAAATCAGCCCAATTAGGTCTCCACCCCTGACTCCCCTCAGCCATAGAAGGCCCC

  721 ACCCCAGTCTCAGCCCTACTCCACTCAAGCACTATAGTTGTAGCAGGAATCTTCTTACTC
```

```
 781 ATCCGCTTCCACCCCCTAGCAGAAAATAGCCCACTAATCCAAACTCTAACACTATGCTTA
                                                                >

 841 GGCGCTATCACCACTCTGTTCGCAGCAGTCTGCGCCCTTACACAAAATGACATCAAAAAA
     >>>>>>>>>>>>>>>>>>>         ***

 901 ATCGTAGCCTTCTCCACTTCAAGTCAACTAGGACTCATAATAGTTACAATCGGCATCAAC
                                                <<<<<<<<<<<<<<<<<

 961 CAACCACACCTAGCATTCCTGCACATCTGTACCCACGCCTTCTTCAAAGCCATACTATTT
     <<<

1021 ATGTGCTCCGGGTCCATCATCCACAACCTTAACAATGAACAAGATATTCGAAAAATAGGA

1081 GGACTACTCAAAACCATACCTCTCACTTCAACCTCCCTCACCATTGGCAGCCTAGCATTA

1141 GCAGGAATACCTTTCCTCACAGGTTTCTACTCCAAAGACCACATCATCGAAACCGCAAAC

1201 ATATCATACACAAACGCCTGAGCCCTATCTATTACTCTCATCGCTACCTCCCTGACAAGC

1261 GCCTATAGCACTCGAATAATTCTTCTCACCCTAACAGGTCAACCTCGCTTCCCCACCCTT

1321 ACTAACATTAACGAAAATAACCCCACCCTACTAAACCCCATTAAACGCCTGGCAGCCGGA

1381 AGCCTATTCGCAGGATTTCTCATTACTAACAACATTTCCCCCGCATCCCCCTTCCAAACA

1441 ACAATCCCCCTCTACCTAAAACTCACAGCCCTCGCTGTCACTTTCCTAGGACTTCTAACA

1501 GCCCTAGACCTCAACTACCTAACCAACAAACTTAAAATAAAATCCCCACTATGCACATTT

1561 TATTTCTCCAACATACTCGGATTCTACCCTAGCATCACACACCGCACAATCCCCTATCTA

1621 GGCCTTCTTACGAGCCAAAACCTGCCCCTACTCCTCCTAGACCTAACCTGACTAGAAAAG

1681 CTATTACCTAAAACAATTTCACAGCACCAAATCTCCACCTCCATCATCACCTCAACCCAA

1741 AAAGGCATAATTAAACTTTACTTCCTCTCTTTCTTCTTCCCACTCATCCTAACCCTACTC

1801 CTAATCACATAA
```

```
KEYS (in order of precedence):
****** target
>>>>>> left primer
<<<<<< right primer
```

(primer3_results.cgi release 0.4.0)

Primer-BLAST - Detailed primer reports

https://www.ncbi.nlm.nih.gov/tools/primer-blast/

Primer pair 1

	Sequence (5'->3')	Length	Tm	GC%	Self complementarity
**Forward primer**	AGGCGCTATCACCACTCTGT	20	60.97	55.00	4.00
**Reverse primer**	TTGGTTGATGCCGATTGTAA	20	55.64	40.00	2.00

Products on target templates

>NC_012920.1 Homo sapiens mitochondrion, complete genome

```
product length = 124
Forward primer  1      AGGCGCTATCACCACTCTGT  20
Template        13176  ....................  13195

Reverse primer  1      TTGGTTGATGCCGATTGTAA  20
Template        13299  ....................  13280
```

PCR Optimization

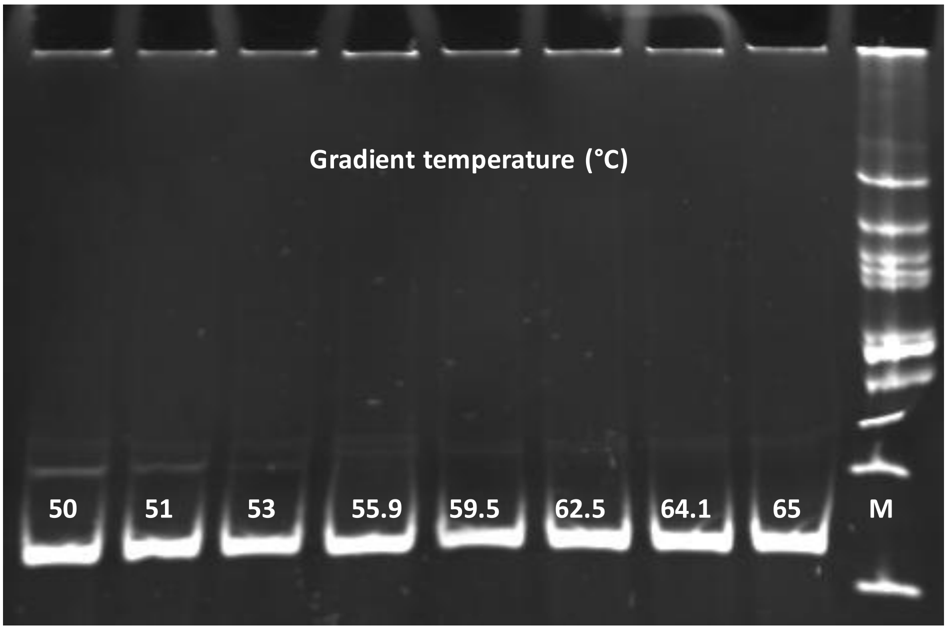

Gradient optimization of a PCR assay for *ND5* gene (small fragment). Gel photo showing PCR products formed at different annealing temperatures in a gradient optimization.

Real-time PCR Optimization

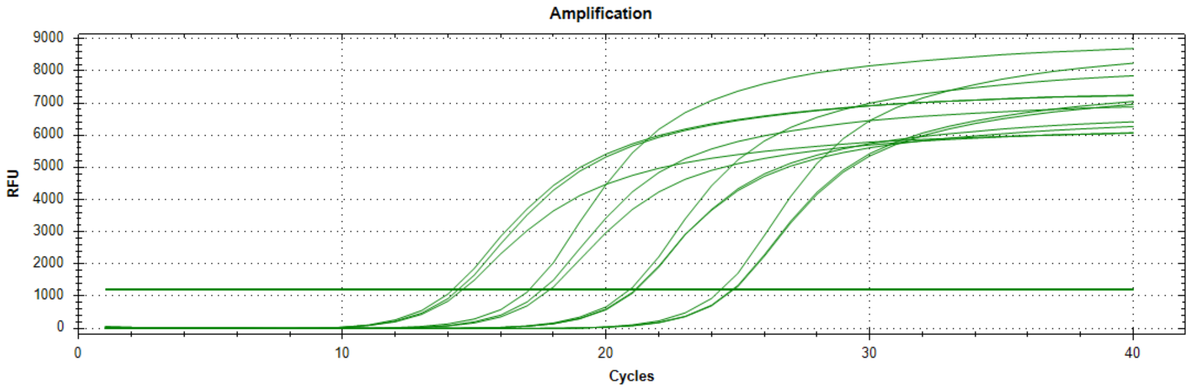

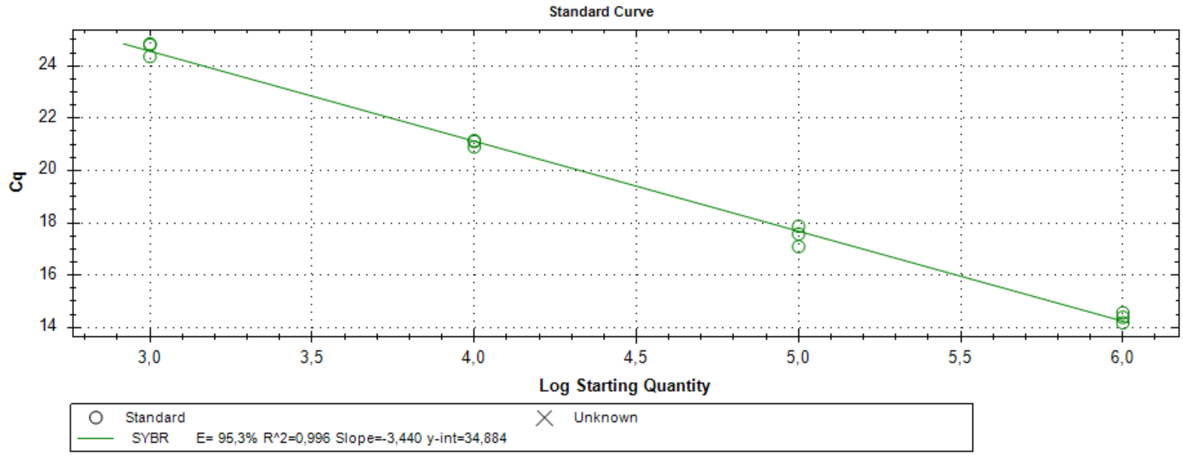

Standard curve with diluted sample: 1/1; 1/10; 1/100; 1/1000.

HPRT1

https://www.ensembl.org/Homo_sapiens/Gene/Sequence?db=core;g=ENSG00000165704;r=X:134460155-134520513

>chromosome:GRCh38:X:134459555:134521113:1
GCCAGGCTGGTCTCGTACTCCTGACCTTGAGTGATCCACCTGCCTCGGCCTCCCAAAGTG
CTGGGATTACAGGCATGAGCCACCGCGCCGGGCTAATATGCTCATTTTAGTGAGGCAAAA
ATAGAGGCTCAGAGTCTGATTTGTACAAAACTACAGAGCAGTTAAGTGTCCTCTCAGATG
TGTACCCTGATCTGGGTGACTCTAGGACTCTAGGTCTCAACTGTTACAACCAGTTAAGGG
TTTGGGGAAGCACTGGGCCAAGAGTCAGGAAAATGGAAGCCACAGGTAGTGCAAGGTCTT
GGGAATGGGACGTCTGGTCCAAGGATTCACGCGATGACTGGAACCCGAAGAGCCGGGGCC
CGGTTTACGGCCGCCATGAAGCAACGCGCGCCGGTAGGTTTGGGAATCAGGGAGCCCTCT
GAATAGGAGACTGAGTTGGGAGGGAAAGGGGCTTCGCTGGGGGAGCCTCGGCTTCTTCTG
GGAGAAAATTCCCACGGCTACCTAGTGAGCCTGCAAACTGGTAGGCGCCGGCGTAGGCGC
GCGGGCGGGGCCGGGGGCGGGGCCTGCGGGGCGTGGCGGGGCGGGCAGAGGGCGGGGCCT
GCTTCTCCTCAGCTTCAGGCGGCTGCGACGAGCCCTCAGGCGAACCTCTCGGCTTTCCCG
CGCGGCGCCGCCTCTTGCTGCGCCTCCGCCTCCTCCTCTGCTCCGCCACCGGCTTCCTCC
TCCTGAGCAGTCAGCCCGCGCGCCGGCCGGCTCCGTTATGGCGACCCGCAGCCCTGGCGT
CGTGGTGAGCAGCTCG**GCCTGCCGGCCCTGGCCGGTTCAGGCCCACGCGGCAGGTGGCGG**
CCGGGCCCTGAGGCGCGGGATCCGCAGTGCGGGCTCGGGCGGCCGGGCCCAGGGAACCCC
GCAGGCGGGGGCGGCCAGTTTCCCGGGTTCGGCTTTACGTCACGCGAGGGCGGCAGGGAG
GACGGAATGGCGGGGTTTGGGGTGGGTCCCTCCTCGGGGGAGCCCTGGGAAAAGAGGACT
GCGTGTGGGAAGAGAAGGTGGAAATGGCGTTTTGGTTGACATGTGCCGCCTGCGAGCGTG
CTGCGGGGAGGGGCCGAGGGCAGATTCGGGAATGATGGCGCGGGGTGGGGGCGTGGGGGC
TTTCTCGGGAGAGGCCCTTCCCTGGAAGTTTGGGGTGCGATGGTGAGGTTCTCGGGGCAC
CTCTGGAGGGGCCTCGGCACGGAAAGCGACCACCTGGGAGGGCGTGTGGGGACCAGGTTT
TGCCTTTAGTTTTGCACACACTGTAGTTCATCTTTATGGAGATGCTCATGGCCTCATTGA
AGCCCCACTACAGCTCTGGTAGCGGTAACCATGCGTATTTGACACACGAAGGAACTAGGG
AAAAGGCATTAGGTCATTTCAAGCCGAAATTCACATGTGCTAGAATCCAGATTCCATGCT
GACCGATGCCCCAGGATATAGAAAATGAGAATCTGGTCCTTACCTTCAAGAACATTCTTA
ACCGTAATCAGCCTCTGGTATCTTAGCTCCACCCTCACTGGTTTTTTCTTGTTTGTTGAA
CCGGCCAAGCTGCTGGCCTCCCTCCTCAACCGTTCTGATCATGCTTGCTAAAATAGTCAA
AACCCCGGCCAGTTAAATATGCTTTAGCCTGCTTTATTATGATTATTTTTGTTGTTTTGG
CAATGACCTGGTTACCTGTTGTTTCTCCCACTAAAACTTTTTAAGGGCAGGAATCACCGC
CGTAACTCTAGCACTTAGCACAGTACTTGGCTTGTAAGAGGTCCTCGATGATGGTTTGTT
GAATGAATACATTAAATAATTAACCACTTGAACCCTAAGAAAGAAGCGATTCTATTTCAT
ATTAGGCATTGTAATGACTTAAGGTAAAGAGCAGTGCTATTAACGGAGTCTAACTGGGAA
TCCAGCTTGTTTGGGCTATTTACTAGTTGTGTGGCTGTGGGCAACTTACTTCACCTCTCT
GGGCTTAAGTCATTTTATGTATATCTGAGGTGCTGGCTACCTCTTGGAGTTATTGAGAGG
ATTATAAGACAGTCTATGTGAATCAGCAACCCTTGCATGGCCCCTGGCGGGGAACAGTAA
TAATAGCCATCATCATGTTTACTTACATAGTCCTAATTAGTCTTCAAAACAGCCCTGTAG
CAATGGTATGATTATTACCATTTTACAGATGAGGAACCTTTGAAGCCTCAGAGAGGCTAA
CAGACATACCCTAGGTCATACAGTTATTAAGAGAAGGAGCTCTGTCTCGAACCTAGCTCT
CTCTCTCTCGAGTAATACCAGTTAAAAAATAGGCTACAAATAGGTACTCAAAAAAATGGT
AGTGGCTGTTGTTTTTATTCAGTTGCTGAGGAAAAAATGTTGATTTTTCATCTCTAAACA
TCAACTTACTTAATTCTGCCAATTTCTTTTTTTTGAGACAGGGTCTCACTCTGTCACCTA
GGATGGAGTGCAGTGGCACAATCACTGCTCACTGCAGCCTCGACTTCCCGGGCTCGGGTG
ATTCTCCCCAGGCTCAGGGGATTCTCCCACTTCAGCCTCCCAAGTAGCTGGGACTACAGG
TGCGCACCACCATCCCTGGCTAATATTTGTACTTTATTTTATTTATTTATTTATTTATTT
TTTGAGATGGAGTTTCGCTCTTGTTGCCCGGGCTGGAGTACAGTGGCATGATCTCGGCTC
AGTGCAACCTCTGCCTCCCGGGTTCAAGCGATTCTCCTACCTCATCCCCCTGAGTAGCTG
GGATTACAGGCGCCTGCCACCATGCCTGGCTAATTTTTTGTATTTTTAATAGAGACGAGG
TTTCACCATGTTGGCCAGGCTACTCTCGAACTCCTGATCTCAGGTGATCCACCCGCCTTG
GCCTCCCAAAGTGCTGGGATTACAGGCGTGAGCCACTGCGCCCGGCCTAATATTTGTATT
TTTTGTAGAGATGGTGTTTTGCCATGTTGTCCAGGCTGGTCTTGAACTCCTGAGCTCAAG
CGATCTGCCCGCCTCTGCTTCCCAAAGTGCTGGGATTACAGGCATGAGCCACCGTGCCTG
GCCTAGGTAGACGCTTTTAGCTTTGGGGTGTGATGCCTGCCCCAGTATATAGTGAATTTA
ATTATTGCTAGAGCTGGCTGTTTGTTAGTTTTCTTTGAACATAAGATACTCATTGTTTTT
AGTTTGCAAATCCCTCTTCCTTTTTAAAAAATTTCTTTCCCTTAAATTGTTTGCATGTTA
GCAATAACAAATGCTTAAATGGTGCTATGTGCTAGATACTCTTCTAAGCCCTGTTATGTA
TATTAACTAATTTTTTAAATTACACAAATCAGAGAGGTTAAGTAACTTGCCCAAGATTAC
CCAACAATACTAGGATTTGAACCTAAGTTTGTCTCACCCCAGATTCTGCTCTTAATCTCT
AAACTTTTAAGTTAGTAGTGACAATAGTAGGTATTTATTGAATACTTAACTATGTTTTAG

GCGTTGAAGTAAATATTTTGCAGGCATTATCTAATGTAAACACCCTAAAGTTACATAACA
GGTACCCTTTAGGTAAATAAACACTAGTATGACCTTGGAGGCACAGATAGTTGAAGTAAC
TTGCCCAATATCACTTACATGAAATTGGCCCTCAAATGTGTCTGATACAACCCATGCTGC
TTGTAACTATCGTTTTAAACTGCCAGGGTAAACTTGGACACACTTGAGCTAAGAAAAAGC
TTTTAGATTTTTGCAAATTAATGTGAAAGATATGCTTTATGTGGATATAATATCTTCTAA
ATTTCGGGGATGGTAGTCCTAGAAATGTAATCCTGCCCTAGCCGAGCTTACCCTGCCAAT
AATTTTTTACAGAATTGGTAAAACGGAGCACCTTTTTTTTGTCCTTGGCCACACTGTTAT
CAACAGGGTGTAGATTGACATCAATCTGTAGGTGTAAACCAGAATTACTCTTTGTGACCA
CCAGGAAATAGAGCAGTTCAGTTCAGGGGTTTCTTTCTGTGAATTTAGCACTGTGACCTG
CATACTACAAGTCTACTTTGTTTTCTATCCATTGTTTGTATCTGGGTATTGCAAAAGGTA
GGAAAAGGACCAACCAGATCAGCAGAGAAGAGTTGCCTTGGAGTTTTCTTTTAGTTTTCT
GCAGTTCATTAGATAGTAACTAGGCCATGTCATTTTACTCCCTTGTAGTGAAGATATGTT
GAAGTTGTACTGGTATACTCTTCTACCTTTCTGTAATTTTATATTGTGTAGACTTGATAA
AATTTATGTGTCAATCACCACCATTAATATCAATATTGAGCCTCAATTCTTATTTTTCTG
CCCAGTGGCTGCCAAATTACTAACATTTACAATAATTCACTACTACTAAGATAATCTACT
AGTTCGATCACATACTTCAAATTGTTATGGAACTACTGTCTTCAGCATTGTGCTTCTGAT
AACTGATAAGTATAATTTTTTTTTTTGTCCAGAGTGAACATGTCTATTCTTCCACTGTACA
CACTAATAAAAGGAAAAATTGTAATATTGGGTAAATTCATGTCCTTACACATGTAGTAGT
TATGAGCCCATGTCCCTAGAATGAGTAATAATTTATCCCTCCCTTGGTTGAATAGTCAAG
AATGCTGATTTTAATTCTTCTAACAGCTTTATCCCTCAGAAGGGAAGGCAAGCAAGTTAT
ATATGTAGTTTATTTGTAAGACTGATATGAAATTGGAAGATGAATCTACTATTAGCTTTA
ATTATTTTTACATTTAGGAATATTGCATCAGTAACTCATAATTTTGGTTTTCTGTTATCC
TGAGTTAACACAAATTATCCAAGGAGATGGCGGATCATCTGCTTTGAGGTGTTTTTTTTT
GAGAATTTTAATGTATCTGAATATAAAAGGTAAAAATATGCCAACTAGCAATTTCTGCCC
ATTCCAGAAGTTTGGAAATATTACTCATTACTAGGAATTAAATAAAATATGGTTTATCTA
TTGTTATACCTCTTTTAATTCACATAGCTCATTTTTATCTTTTATTTTTGTTTGTTTTTT
TTGAGATGGAGTCTTGCTCTGTCACCAGGCAGGAGTGCAGTGATGCAATCTCGGCTCACT
CTAGCCACCGACTCCCTGGTTCAAGCGATTCTCCTGCCTGAGCCTTCTGAGTAGCTGGGA
TTACAGGCAGGCACCACCACGCCCAGCTAATTTTTGTAGAGACAGGATTTCACCGTGTTG
GCCAGGATGGTCTCCATCTCCTGACCTCATGATCTGCCTGCTTCGGCCTCCCAAAGTGCT
GGGATTACAGGTGGGAGCCACTACGCCTGGCCCACATAGCTCATTTTTAGACTCACTTCC
ATTAAGTCTTGTTTGGACCCACGAACATTGTCTTTTTTTTTTTAAGATGGAGTTTCACTT
TTGTTGCCCAGACTGTAGTGCAATGGTGCAATCTCAGCTCACTGCAATCTCTGCCTCCTG
GGTTCTAGCAATTCTCCTGCCTCAGCCTCCCGAGTAGCTGGAATTACAGGCGCCCGCCAC
CACGCCCAGCTAATTTTTGTGTTTTTAGTAGAGACGGGGTTTCACCATGTTGGGCAGGCC
AGGGGTGATCCGCCCACCTCAGCCTCCCAAAGTGCTGGGATTACAGGTGTGAGCCACCGC
ATCTGGCCAACATGTCTTTTTTTTTTTTTTTCCTTTTTAACCACAAAGAGACTTAAGCAGT
CCTTGTCACAGATGATGAATTGATGTTGCAAGTATTGTCTTAGCTTGGATTAATTTTCTT
GCTTACTGTAATTTTAGATAATATAGCTTTGTAATTAGAGATTTTATGTGTAAACCACAA
AAATGTTTACATGAAGGCCATTATTACAGATGTGACGTGCATAATTATTAGTAATTTGTA
TGTTTACATGGGTCAGTCTGGCAAAAAATTATGAAGTTTTAAAAATTAAAAAAAATTATA
ATGCCAGTTTTACTGGAAAGTAAAATTATTTCAGTAATCGATTATAGCAAAAGTATTGAT
TTTCATTCCAGACAAAAGTCAGAATGAAAGGTAATTTCTCAATACTCTTTCAGATTAATA
AAAGTACCTGTAGCGATTTTTATCATTCACAAGTATATCACAAGTAAGTTAGAATTTGAG
AACTGTGTTCTAGATCTCTGAGGAGATGCAGTCAGATTTCTGAACTGTCTCAGCAAATGG
TAAGTAACTTAGAGCTAGTAATTAATAACCTGTCCTTTGATTTCTGATTCAGCCAAGAAT
GGCCATATTTGGGAAAGGCAGATCTGGAGAGTAACCACGTTTTCATTCATTTACCACTTC
TAGGCCCCTCCAGAGCTCTCAGATATTTTGGGGTTGAGCCCTTCCCCAAAGCCATACAGG
ACCTTTTTTTTGTGATCTGTTCTAGCCATTTTTATGTTGGGTGCTTGTTATGGACTGAGC
ATTTATGTCCTCCCACACCCCCCCCATACCTTTTTTGAAGTCCTAACCCCCAGTGTGATG
GTATTTGGAGACAGGGCCTTTGGAAGGTAATTACAGTTAGAAGAAGTCGGGAGGGTTGGG
CCCAGGTCTGATTGGATTAGTGCCCTTATATGAAAAGACACCAGGACGGGCGCAGTGGCT
CACACCTGTAATCCCAGCACTTTGGGAGGCCAAGGTGGGTGGATCACGAGGTCAGGAGTT
TGAGACCAGCCTGGCCAATGTAGTGAAACACCATCTCTACTAAAAATACAAAAATTAGCT
GGGTGTGGTAGCGGGCTCCTGTCATCCAAGCTACTCGGGAGGGTGAGGCATGAGAATCAC
TTGAACCCGGGAGTTGGAGGTTGCAGTGAGCCCAGATTGTGCCACTGTACTCCAGCCTGG
GTGACAGAGTGAGACTCTGTCTCAAAAAAGAAAAAAAAAAAAAAAAGAGACACCAGAGAGC
TTGTTAGAAGAGGTCATGTGAGCACACAGTTAGAAGACCTTCAAGCCAAAGAAGAGGCCT
GAGATTGAAACCTACCTTGCAGGTACCTTAATTTTGGACTTCCCAGCCTCCAAAACTGTG
AGAAATAAGTTTCTGTTAAGTCACTCAGTCTGTGGTATTTTGTTATGGCAGCCTGAGCAG
GTAGTTGTTCTTTCAGAAGGTGTTGATAATAACCACATGCAACACCAAGTCACAAATAAT
AAAACAGATGTAACTTATATTCATACAGAAAGTTGGGCACTGCCATTGCCTTGTTGGTTT
ACACGGCTGTGCTAGTTCAGTAGCAGAAAGGTGCTGGTCTCCTTTACTCAGTTTACAATC
TAGGCAGTAGAATGTAATCACTGCTTTAAACTTGATACTGCTTAGGGAGAGAATCATTGG
TGCTGGGTAACTTTGGGTTCTAGGTTTACTTTTTGTGTATATATAACTGTTTTTGGTAAA

TCACAAGTTTCTGGGCTTGTCGAATTAGATTTTGTTACAGATTATGAGCTTTATTATGCT
ATACAGTTAGTTGTATGTATATATGCCTTTCCCACTAGATTTTAAGCTTTTTTTTTTTTT
TTTTTTTTGTGACGGAGTCTTGCTCTTGTCGCCCAGGCTGAAGTGGAGTGCAGTGGCACA
ATCTCGGCTCACTGCAGCCTCCACCTCCTAGGTTCAAGCGATTCTCCTGCCTCGGCCTCC
CAAGTAACTGGGACTACAGGCACGTGCCACCACACCCGGCTAATTTTTGTATTTTTTGTA
GAGACAGGGTTTCGCCATGTTGGCTAGGCTGGTCTTGAACTTCTGGCCTCAGGTGATCCA
CCCGCCTCAGCCTCCCAAAGTGCTGGGATTTACAGGCATGAGCCACCACGCCCAGCTATA
AGCTCTTTAAGGGTTGTAAATTTATAATCATTCTTTTACTCTCCTGCAAATTCTGTTGCA
CACTGCCTTAATCAAGGTAGATGCTGAATGCATTTTTGTATAATTGAATATGTTGCAATC
CCCAACTCTCTCCAACTGTTCCTGTCAAAGCAGCCACTGGATTGTTAACTAATCCATATT
AGATGGGGTTAATTAATATCAGATGGGACAAGTAAGGGCTAATAAGATTATAGGCCACCA
AGTAGATTTCTGTCTAGCTCTTATAGAGATTGAGTTTATTGGACCTGTTTGATAGGAAGT
TTTGGTGTTTGGGATGATTAAAACTGAAGTTCCTATTTATTGAATTATACCTATTTATAT
TATTTCATATCAGTGGTCCACATGCAAGTGAGGCTTCTGAGACAGAGTTTGAGTTCTCTC
TTCAACTACCATAACACTTAACCTGTATCTTTTTTTTTTTTTTTTTTTTTAGACGGAGTC
TCGCTCTGTCACTCAGGCTGGAGTGTAGTGGTATGATCTCGGCTCACTGTAACCTCTGCC
TCCTGGATTCAAGCAGTTCTCCATGTCTCAGCCTCCCTAGTAGCTGGGATTACAGGCCTG
TGCCACCATGCCTGGCTAATTTTTTTTTTGTATTTTTAGTAGAGACGGGGTTTTACCACG
TTGGCCAGGCTGGTCTCGAACTCTTGACCTCGAGCGATCAACTTGCCTTGGCCTCCCAAA
GTGCTGGGATTACAGGCATGAGCCACAGCGCCCAGCCGTCTTTTTTTTTAAATAGCAATT
TAACACTGTTCACAGTTACTCATGTACATGTCATGCCATCTATTACACTGTAAGTTCTGT
GAGGGTAGCTGTATCAAATTTATCTAACTCTCTCTAGTATGCATGACATAGTAAGTATTC
AATAAATATTTGCATATTAGTGATAAGGATACAGGTTCTGAATAGTGGGTCCTTACCATT
TAAGAATTAGTATTTGATGGCCGGGCGGGGTGGCTCACGCCTGTAATCCCAGCACTTTGG
GAGGCTGAGGCGGGCGGATCATGAGATCAGGAGATCGAGACCATCCTGGCTAACATGGTG
AAATCCCGTCTTTACAAAAAAAAATACAAAAGAATTAACCAAGTGTGGTGGTGGGTGCCTG
TAGTCCCAGCTACTGCTTTGTGAGGCTGAGGCAGGCAGATCACCTGAGGTGGGAAATTCA
AGACCAGCCTGACCAACATGGAGAAACCCCATCTCTACTAAAAATACAAAATTAGCCGGG
CGTGGTGGCGCATGTCTGTAATCCCAGCTACTCGGGAGGCTGAGGCAGGAGAATGGCGTG
AACCCGGGAGGCGGAGCTTGCAGTGAGCCAGGATCGCGCCACTGCACTCCAGCCTGGGCG
ACAGAGCGAGACTCCGTCTCAAAAAAAAAAAAAAAAAAAAAAAATTAGTATTTGATATTTGA
TCATTAAATATGAATTAAGAGGACTTAGACTTTTTGTTAAATGTCAAGCTGGGAAAAGTT
GTCATTTAAATGAATTGCCTCTTATTTAATTTCGTCTGATGATACATTTTGTTTTTATTT
TGTAAAAAATTATTTTTTTTCTTTTTGGAGACAGGGTCTTGCTCTGTTGCCCAGGCTGGT
CACAAACTCCTGACCTCAAGCAATCCTCCTGCCTTAGCCTCCCAAAATGCTGGGATTACA
GGCGTGAGCCACCTCGCCCGGCCTTGTATTATGATACATTTTGAACAACTACAAGTAGAC
TTGGTATAATGAACCTGCACGTACCCATTGCCAAGTTCTGACAACTGTCTGTCTATAGCC
AATTATGCATTTCTTAAATTAGAACCCCCCCAATATACCCAAATATATATATATGTGTGC
ATATATATAGTAAGTTGTAACAAAGTTGTGAATTCATACCTGAAGTATCTCAAGTGATGC
AAGTTTTATGAATTTTTGTTTATGCCTTTTGGGAAGAGTTGTATTGACAAATTTTTTATG
CTTAAAGTAAACCATAAATCAAAAAAATAAAATCTAGGATGCAATAAAACAAAACAACTT
CTTGACATAAGTATGGTATGTAAATCTGTTTTGATTGGAAATCAATTTGTTATATTGCCA
GAATTCCTGTTTTAGAATACATCTCTGCTGATCTGTCTGTATTCTTAGACTGCATATCTG
GGATGAACTCTGGGCAGAATTCACATGGGCTTCCTTTGAAATAAACAAGACTTTTCAAAT
TCTTAGTCGATCTGCAGAACCTGTAGCCAGGCACTGAACCATTTTGATAGATGCAGTAAT
CGTTGCAAGTGTATATTTCAAGGAGTTCTGGCTGGGTCCTAGTTTATGCTTGTGGCAGAA
GCAGTGAGTAACTGGGAGGAAGTTGGTGAGTAAGCTTCAAGGAAGAAGTCATTTTTAGTA
CTCTGGATCTTCCTGATTTTAAAGCACTACAAAATGGTGCATTTTCATTCTTGTCAAGTG
ATAACAGATATATTCTGATGAGCCTGAAATGAATATATATTGTATCATTTTTATAATATC
TAGCAAGGTTTGTATTTTCCTAGAACTTGAACTAAATTTCAGTTCATAAAATTTATAAAA
TACTTAGTTGTTGTAAAATATTTTTGGAATGTTCACATAGGTGACACACAAATGTCCCAT
TTTCATTCTTTCTATAGTAAATATGTTCTGATATGTGAAGGTTTAGCAGATGCATCAGCA
TTTAATCCTAGAGGATCTGGCATAATCTTTTCCCCCAAGAATAGAAATTTTTTCTGCTTA
TGAAAGTAGTACATGTTTCTTTAAAAACAAATCAATATTGACTTCTGCCTGCTGTATAGC
ACTATGCCTCCACCTGGCCATGACCAGGGGCATGTCCTGGTCCACCTACCTGAAAATGTT
TGCAACCAGCCTCCTGGCCATGTGCACAGGGGCTGAAGTTGTCCCACAGGTATTACGGGC
CAACCTGACAATACATGAAGTTCCACCAAAGTCTGAGAACTCAGAACTGAGCTTTGGGGA
CTGAAAGACAGCACAAACCTCAAATTTCTCAGCACTGGAAACCTCAAAATATAACTGAAT
TCCATAAATAAGATTTTAAGTCTTAAATATGTATTTTTAAATGTATTAAAAGTCAAGCTG
CTTGTATTTAAGCACCTAATACAATGCTTAGGTTGTAAAAGGAGATGCTCAATAGGTACT
AACTGATATATTGAGATTTAATTATGGTTTGACCAATATTTATTGGAAACCGCCAAAGCT
TAAATCATCAGCTTCTTGAATGTGATTTGAAAGGTAATTTAGTATTGAATAGCATGTGAG
CTAGAGTATTTCATTCTTTCTGGTTTATTTCTTCAAATAGACTTTGAATATAATGGTGAA
TGGGTATTATAAATTAACTAATAAAAATGACATTGAAAATGAAAAAATATATATATTAAA
GTGTAGAAAGTGACCAGGCGTGGTGGCTCACACCTGTAATCCAAGCACCTTGGGAGGCTG

AGGCAGGAGGATCTCTTGATCCCAGGAGTTCAAGACCAGCCTGGGCAACATAGCGAGACT
TCGTCTCTAAAAAAAAAAAAGAGAGAGAAAAAAATTTTTTTATTTAAAAAAAGTGTAGA
AAGTGTCAAGACCCCACTTCTTACCATTATTTGGTATATTTCTCTATACCCACCCACCCT
TCCTCCTTACTCCCTCCCTCCCTTCCCAATCTTTTTATCTTTTTGTATTCTGATTTTTTG
TTTGTATATTTTGCTTTAATTTAATGTATCCTTTAAAAATTTCCCATACATTTTATATGT
ATATATAAAAACGCATGCTGCCAAAGATAATTTATAAGAAAGACCATTGAATTTTTTTAA
AAGTGATATATATTCATTGAAAAAAATTTAGAATATATAGCAAAGCAATAAAGAACTAAA
TAAAATTGCTGTAACTCCTCTTTCAAAGATAAGTGCTTTTATGATTTTGTTGTATTTTTT
TCTGTATATAGGTACATATATAGTATTTATAAAGCTGTACTCATAGTACATTTTCACATC
ACAGGTACCATATCAGTGTTATTAAATATTTTGTATGCCAGGGGCTAGACATACCAAGAC
AACCAATATGTGGTTCTACTTAAATAATATTAGAGTATCTTTTATGATGACACTTCATGA
GTTGACTATAATAATCTTAGACTTCTAAGAGTTTGGGTTTTCAAAAGATCACTTAGCTTT
TTTGGGTGATTTTTCCCCCTTACTGTGAGATGAGAGAGGCTGTTTGGATTTGGGATTGGG
GTAGCGGGGACAGCAACTTTTCTTTTCTTTTTCTTTTTTATTTTGAGGTAGGGTATTGCT
GTGTCACCCAGGCTGGAGTGCAGTGGTGTGATCTCGGCTCACTGCAACCTCCACCTCCCG
GGCTCAGGTGATCCTCCTGCTTCAGCCTCCCCAGTAACTGGGACTACAGGCGCGTGCCAC
ATGCCTGGCTAATTTTGTATTTTTAGTAGAGATGGGGTTTCACCATGTTGGCCAGGCTGG
TCTCTAACTCCTGACCTCAGGTGATACGCCCACCTGGGCCTCCCAAAATACTGGGATTAC
AGGCATGAGCCGCTGCATCAGCCAGCAGTTTTTCTTGTGGTTTTTTTTGTTTGTTTTGTT
TTGTTTTGTTTTTGAGATAGGGTCTTACTCTGTTGTCCACGCTGGAGTGCTGTGGTATGA
TCGTAGCTCACTGCAGCCTCAAACTCCTGGGCTCAAGTGATTCCTTCTGCCTCCGCCTCC
CGAGTAGCTGGGACTACAGGTATGCACCACCATACCTGGCAAATTTTTACAAAGTTTTTT
GTAGGGACGGGGTCTTGCTACATTCCCCATGTCGGTCTTGAACTCCTGGCCTCAAGCAAC
TCTCCTGTCTCAGCCTCCCAAAGCACTGGGATTACAAGTGTGAGCCACCACACCATGCCA
GTTTTTCCTGTTCAGTGTGATATTTTATCTTGTTAGACTACAGTGTGTTAAAACTTGTTT
TACTAAATTTTCAAACATACTCAAAAGTGGAGAGAATAGTATAATGAATACCCGTATGTT
CATCACCCATGTTTAGAATATTATTAAATATAAAGATTTTGCTGCGTTTGTCTTAGCTCT
TTAAAATTTTTCTTTTTCTCTTTGTGACCTAAAGGAAATTCCATATCTTATCACTTTACT
TCTACATTCTTGACTAAGATGACTAAGACATATAGTTACATGGTTTTTTGTTTTGTTTTT
TGTTTTTTAAAGACGAAATCTCGCTCTTGTCCCCCAGGCTGGAGTGCAATGGTGCCATCT
CAGCTCAGTGCAACCTCTGCCTTCTGGGTACAAGCGATTCTCCTGCCTCAGCCTCCCAAG
TAGCTGGGATTACAGGCTCCTGCCACCACGCCTGGCTAATTTTTGTATTTTTAGTAGAGA
CGGCGGGGGGAGGTTTCACCATGTTGACAAGGCTGGTCTGGAACTCCTGACCTCAGGTGA
TCCACCCGCCTCGGCCTCCCAAAGTGCTGGGATTACAGGCGTGAGCCACCGCGCCCAGCC
TGTTTTTTTGTTTGTTTGTTTTGTTTTTTTTGAGACAGAGTCTTGCTCTGTTTCCCAGGC
TGGAGTGAAGTGGCGCATTCTTGGCTCACTGCAACCTTCACCTCCCAGGTTCAAGTGATT
CTCCTGCCTCAGCCTCCCAAGTAGCTGGGACTACAGGCATGTGTCACCACACCCGGCTAA
TTTTTTTGTATTTTTAGTAGAGACGGGATTTCACCGTGTTGCCCAGGCTGGTCTCGAACT
CCTGAGCTCAGGCAGTCTGCCTGCCTCAGCCTCCCAAAGTGCTGGGATTACACGTGTGAA
CCAACCCGCCCGGCCTGTTGTTTTCTTACATAATTCATTATCATACCTACAAAGTTAACA
GTTACTAATATCATCTTACACCTAAATTTCTCTGATAGACTAAGGTTATTTTTTAACATC
TTAATCCAATCAAATGTTTGTATCCTGTAATGCTCTCATTGAAACAGCTATATTTCTTTT
TCAG**ATTAGTGATGATGAACCAGGTTATGACCTTGATTTATTTTGCATACCTAATCATTA**
TGCTGAGGATTTGGAAAGGGTGTTTATTCCTCATGGACTAATTATGGACAGGTAAGTAAG
ATCTTAAAATGAGGTTTTTTACTTTTTCTTGTGTTAATTTCAAACATCAGCAGCTGTTCT
GAGTACTTGCTATTTGAACATAAACTAGGCCAACTTATTAAATAACTGATGCTTTCTAAA
ATCTTCTTTATTAAAAATAAAAGAGGAGGGCCTTACTAATTACTTAGTATCAGTTGTGGT
ATAGTGGGACTCTGTAGGGACCAGAACAAAGTAAACATTGAAGGGAGATGGAAGAAGGAA
CTCTAGCCAGAGTCTTGCATTTCTCAG**TCCTAAACAGGGTAATGGACTGGGGCTGAATCA**
CATGAAGGCAAGGTCAGATTTTTATTATTATGCACATCTAGCTTGAAAATTTTCTGTTAA
GTCAATTACAGTGAAAAACCTTACCTGGTATTGAATGCTTGCATTGTATGTCTGGCTATT
CTGTGTTTTTATTTTAAAATTATAATATCAAAATATTTGTGTTATAAAATATTCTAACTA
TGGAGGCCATAAACAAGAAGACTAAAGTTCTCTCCTTTCAGCCTTCTGTACACATTTCTT
CTCAAGCACTGGCTATGCATGTATACTATATGCAAAAGTACATATATACATTTATATTTT
AACGTATGAGTATAGTTTTAAATGTTATTGGACACTTTTAATATTAGTGTGTCTAGAGCT
ATCTAATATATTTTAAAGGTTGCATAGCATTCTGTCTTATGGAGATACCATAACTGATTT
AACCAGTCCACTATTGATAGACACTATTTTGTTCTTACCGACTGTACTAGAAGAAACATT
CTTTTACATGTTTGGTACTTGTTCAGCTTTATTCAAGTGGAATTTCTGGGTCAAGGGGAA
AGAGTTTATTGAATATTTTGGTATTGCCAAATTTTCCTCTAAGAAGTTGAATCATTTTAT
ACTCCTGATGTTATATGAGAGTACCTTTCTCTTCACAATTTGTCTCTTTTTTTTTTTTTT
TTGAGACAAGGTCTCTGTTGCCCAGGCTGGGGTGCAGTGCAGCAGAATGATCACAGTTCA
CTGCAGTCTCAACCTCCTGGGTTCAAGCGATCCTTCCACCTCAGCCTCCTGAGTAGCTGG
GACTATAGGTGTGCGCCACCACTCCCAGCTAATATTTTTATTTTGTAGAAACAGGGTTCG
CCATGTTACCCAGCCTCCCAAAGTGCTGGGATTACAGGCATGAGCCACTGGCCCAGTTTC
TACAGTCTCTCTTAATATTGTATATTATCCAAGAAATTTCATTTAATCAGAACCTGCCAG

TCTGATAGGTGAAAATGGTATCTTGTTTTTATTTGCATTTAAAAAAAATTATGATAGTGG
TATGCTTGGTTTTTTTGAAGGTATCAAATTTTTTACCTTATGAAACATGAGGGCAAAGGA
TGTGTTACGTGGAAGATTTAAAAAAAATTTTTAATGCATTTTTTTGAGACAAGGTCTTGC
TCTATTGTCCAGGCTGGAGTGCAGTGGCACAATCACAGTTCACTCCAGCCTCAACATCCT
GCACTAAAGTGATTTTCCCACCTCACCTCTCAAGTAGCTGGGACTACAGGTACATGCTAC
CATGCCTGGCTAATTTTTTTTTTTTTTGCAGGCATGGGGTCTCACTATATTGCCCAGGTTG
GTGTGGAAGTTTAATGACTAAGAGGTGTTTGTTATAAAGTTTAATGTATGAAACTTTCTA
TTAAATTCCTGATTTTATTTCTGTAG**GACTGAACGTCTTGCTCGAGATGTGATGAAGGAG**
ATGGGAGGCCATCACATTGTAGCCCTCTGTGTGCTCAAGGGGGGCTATAAATTCTTTGCT
GACCTGCTGGATTACATCAAAGCACTGAATAGAAATAGTGATAGATCCATTCCTATGACT
GTAGATTTTATCAGACTGAAGAGCTATTGTGTGAGTATATTTAATATATGATTCTTTTTA
GTGGCAACAGTAGGTTTTCTTATATTTTCTTTGAATCTCTGCAAACCATACTTGCTTTCA
TTTCACTTGGTTACAGTGAGATTTTTCTAACATATTCACTAGTACTTTACATCAAAGCCA
ATACTGTTTTTTAAAACTAGTCACCTTGGAGGATATATACTTATTTTACAGGTGTGTGT
GGTTTTTTAAATAAACTCCTTTTAGGAATTGCTGTTGGGACTTGGGATACTTTTTTCACT
ATACATACTGGTGACAGATACCCTCTCTTGAGCTACATCGGTTTGTGGGGAGTCAAAAGT
CCTTTGGAGCTAGGTTTGACAAATAAGGTGGGTTAACACTTGTTTCCTAGAAAGCACATG
GAGAGCTAGAGTATTGGCGAATTGAAGAAATCCCCCTTTTTTTTTAACACACTTAAGAAA
GGGGACTGCAGGTATACTCAAGAGAGTAAGTCGCACCAGAAACCACTTTTGATCCACAGT
CTGCCTGTGTCACACAATTGAAATGCATCACAACATTGACACTGTGGATGAAACAAAATC
AGTGTGAATTTTAGTAGTGAATTTCATTCATAATTTGATCGTGCAAACGTTTGATTTTTA
TTACTTTAGACTATTGTTTCTGATTTTATGTTGGGTTGGTATTTCCTGTGAGTTACTGTT
TTCCTTTAAAATAGGAATTTTTCATACTCTTCAAAGATTAGAACAAATGTCCAGTTTTTG
CTGTTTCATGAATGAGTCCTGTCCATCTTTGTAGAAACTCGCCTTATGTTCACATTTTTA
TTGAGAATAAGACCACTTATCTACATTTAACTATCAACCTCATCCTCTCCATTAATCATC
TATTTTAGTGACCCAAGTTTTTGACCTTTTCCATGTTTACATCAATCCTGTAGGTGATTG
GGCAGCCATTTAAGTATTATTATAGACATTTTCACTATCCCATTAAAACCCTTTATGCCC
ATACATCATAACACTACTTCCTACCCATAAGCTCCTTTTAACTTGTTAAAGTCTTGCTTG
AATTAAAGACTTGTTTAAACACAAAATTTAGACTTTTACTCAACAAAAGTGATTGATTGA
TTGATTGATTGATTGATGGTTTACAGTAGGACTTCATTCTAGTCATTATAGCTGCTGGCA
GTATAACTGGCCAGCCTTTAATACATTGCTGCTTAGAGTCAAAGCATGTACTTAGAGTTG
GTATGATTTATCTTTTTGGTCTTCTATAGCCTCCTTCCCCATCCCCATCAGTCTTAATCA
GTCTTGTTACGTTATGACTAATCTTTGGGGATTTGTGCAGAATGTTATTTTAGATAAGCA
AAAACGAGCAAAATAGGGGAGTTTAACTTTAATATTTTCTTTTAAAAAGCATTTCATGTT
ATAAGATCAATTCTGAGTGGTAGAAATGCTTTGACATTTTATTTCCATTTTCTACTTTTA
GTTTTTTTCCTATTTGTTTAAGATCTTAGAGGATTATTAAGCTGAACTCCTCAACTGATA
AAAAGCATGACATCTTAAACATAAGCAAAGCATATTTTTAGGTTAATTTTCACATAGAAA
ACAGTTTATTTTATGTGAAATTCTATGTAGATATACTATTTTTTTGGTATTTATTGATAT
GTTTATTTTATTTTATTTTATTTTATTTTATTTTATTTTATTTTATTTATTTATTTTTTT
TTTTGAGACAGAGTCTCACTCTGTTGCCCAGGCTGGAGTGCAGTGGCATGATCGTAGCTC
ACTGCAACCTCCACCTCCCGGGTTCAAGCAATTCTTCTGTCTCAGCCTCCCGAGTAGCTG
GGACTACAGGTGCCTGCCACTATGCCCGGCTAATTTTTGTGTTTTTAGTAGAGATGGGGT
TTCACCTTGTTGGTCAGGCTGGTCTCGAACCCCTGACCTCAGGTGATCCACCCACCTCAG
CCTCCCAAAGTGCTGGGATTATAGGCATGAGCCACCGTGCCCGGCCGACATGTTAATTTT
TTAAAAAAGGCTTTACTGGGGTATATTTTATATAATATAATAATCACATGTTTTAACTAT
ACAATTCCAAGCTTTTTAGTATATTTATAGGGCTATGCAAGGAAGATATACTGTTAAACA
GTAGAAATTGAGAAAGCTCTTCTGATAATATCTCTTGATTTGATGATGGCTCATGCCTGT
AATCTCAGTGCTTTGGAAGGCCAAGACAGCAGAATCACTTGAGGCCAGGGGTTCGAGACC
AGCCTGGGCAACACAGCAATACCCTATCTTTACAAATAATAAAAATATCTGTTGATTTGA
AGTAAAGTTTTTTTTTAAAGACAAGGTCTCATTCTGTCACCCAGGCTGGAATGCAGTAGC
AAGATCACAGCTCACTGTGGCCTTGACCTTCTGGGCTCAAGTGATTCTCCCACTTCGGCC
TCCCGAGTAGCTGGGACTAACAGGTGTGCACCACCATGGCTGGCTAATTTTTTTTTATGT
TTGTAGAGATTGGGTCTTACTGTGTTGCCCAGGCTGATCCCGAACTCCTGGGCTCAAGCA
GTCTTCCTGCCTCAGCCTCTAAAATTGCTGGGATTACAGGCTTGAGTCACCATGCCCAGC
CTGAAGTAGCATTTCTACCCTGTTTAATAATTCAGCAGCTTGTCATGTAAGATATTCATA
TATGCATATAAAACATTAGGCAGCTTAATTTGGTAAAACTGTAAAATGGAAATTTTAAAT
TGTTTGCAGCATCAATAACATTGATGTCAGTATGATTTTTACATGCTGATCTTGACCAAT
TTGAAACAGTGAGTTAAAATCTGGCTGATCCGTACTAATCCTAAAGAAATATTCTATGAA
CTATTAAATGTTTCCAGAATATATAAAGAAACATTATGATGTCAACACACCCATCTATTT
TTTTTTGGAAATAAAAACTCCATTTTTCTTATTAAAGAAAACATGCTTATTAGAAAACAT
ACGGCTGGGTGCAGTGGCACACATGTAATTCCAGTGCTTTGGGAGATCGAGGTGGGAGAA
TCACTTGAGGCCAGGAGTTTGAGACCAGCCTAGACAACATAATGAGACCCCCTCTCTACA
CAAAAAGAATTAGTTGTGCATGGTGGCGTGCACCTGTAGTCCCAGCTACTTGGGAGGCAG
AGGCAGGAGCATCCCTTGAGCCTAGGAGTTTGAGACTGCAGGAGTTCGAGACTGAGTGGA
ATGCAGTGGAACTGCATTCCAGCCTGAGTGACAGAGGGAGACCCTGTCTTAAAAAAATAA

GAAAGAAAACACAACTGCAGAAAATTATAAAGGATTTAAGTCATTCCAAATATCACTGCC
ACTTTTTATTTAGAATATTCTAAAGAATTCTCTCTCTGTGTACACACACACATATGCGTA
CTCTTAATCCAAGTAGCTTGGTAGGATTTTATTTACCTAGTGCCTAGATGGGAAATTGCC
TGGGGATTCCAAATACCTATTTCATTAAATTAAAGATGTCACTGATTTTAAGACTTAACA
CTATTTTTCATACTGCCAAGAAAGAAAACACTACCAGTTATAAATGTAAATTGCCATCAA
TTGTAATACATCAATTTTAGAGCTATTATTAATAAAATGTGAATGTGCATCTTAGAGCAA
TGAAATATAGTACTATATATTTGATGACCTTTTCTGCCCTGTGATATTCAGAAAGTGAAA
GTTAAATATGGGCTGAGCATGGTGGCTCACACCTGTAATCCCAGTACTTTGGGAAGTCAA
GACGGGAGGCTGGCTTGAACCCAGGAGTTCAAGACCAGCCTAGGCAATGTAGCGAGACGC
CATCTCAAAATATTAAAAATAAGTAAATAAGTAAATAAAAAGAAGGTTAAGTATACAAAT
GTATTTCCTTTGTTGTGAATTTATTTCAATTTTATAGTGATTTTTTTTTTTTGAGACGAA
GTCTCACTCTTGTCCCCCAGGCTGGAGTGCGATGGCGTGATCTCAGCTCACTGCAACCTC
TGCCTCCCAGGTTCAAGCTATACTCCTGCCTTGGCCCCCCGAGTAGCTGGGATTACAGGC
GCCTGCTACCATGCCTGGCTAATTTTTGTATTTTTAGTTGAGATGGGGTTTCACCATGTT
GGCCAGGCTGGTCTAGAACTCTTGACCTCTGGTGATCCACCCGCCTCGGACTCCCAAAAT
GCTGGGATTACAGGCGTGAGCCACCGTGCCTGGCCAGTGGTTTTTTGTTGTTGTTGTTGT
TGTTGTTTTGTTTTTGTTTTTGTTTTTGTTTTGAGACAGGATCTTGCTCTGTCACCCAGG
CTGGAGTGCAGTGGTGCCATCTTGGTTCACTGCAACCTCTGCGTGGGCTCAAGCAATCCT
CCCACCTCCCTTTCCAGAGTAGCGGGGACCACAGGTGTGTGCCACCACACCTGACTAATT
TTTGCATTTTTTTTTGTAGAAACAGGGTTTTGCCATGTTGCCCAGGTTGGTCTGAAACTC
CTGAGCTCAAACAATCCAACTGCCTTGGCTTCCCTAAGTGAAATTACAGGCATGGGCCAC
TGTACCCAGTCTAGTGATTTTTTTATTTTTATTTTTATTTTATTTTATTTTATTTTTTTA
CCAAAAAAACAACAAAGCCTCAGGAGGAAAAGTTGATACACAAGTAAATTTTATTGGAAA
TGTTTTTGTGTGGACCTTAAGCAGAGGGAAAATTAGTCTGCATTATGGTGTATCCAGACT
AAATGACTGATATTAAAATGAAATTATTCTTAGGATTTGCAATCTTAGAGAAAACTTTTT
CATTTTTATTTTTTTGAGTTACAAATTATCTTCATTTACATTTGAGAACAGTGAGTCACA
GAGGGATTAAGTAACTTACTCAAGATCATACAAGTCTTTGATTTGAACCCAATCTTTTAA
CTCTGCAGAACTCAGAGTCACTCTTATTTGGAAAAACTTTTTAACTGATGTGGATCCTCT
AATATGGGCTTCCTATTATTCATTCTCTATTAGTCAGAAGTTTTGCAAGCAGACAGAATT
CATTTTGCCAATTACGGGATTTTCCCTCAGTTGCAGTCAAGGTTCATAAAACTATAACTC
TTTATCTTTAATTAGAAATGTTTTTTTTTTTGAGACAAGGTCTTGCTCTGTTGCCCAGAC
TGGAATGCAGTGGCATAGTGGCCCATTGCAGCTTTGAACTCCTGGGCTCAAGGGATCCTC
TGCCTCAGCCTCCCAAGTATCTGAGACTACAAGTGCGTGCCATCACCCATGGCTATTTTA
AAAAAAAAAAAAATTGTAGAGATAGGGTCTTGCTGTGTTGCCCAGGCTGGTCTCAAACTC
CTGGTCTCAAGCAATCCTTCTGCCTTGGTCTCCCAAAGTGCTGAGATTACAGGTGTCAGC
CGTTGCACCTGGCCAAAACGATAACTTAAAATACACACACACACACACACACACAAACAC
ATATGTGTATTTGTGTGTGTGTGTGTGTGTGTGTGTGTCTCAAAAGGTATCAAAAGAGAATA
GCTATAACTTTAGTGTTGATCTTGATAGTGACTTGATTAGGCTCTGTTTAACATCAAAGA
TGCAAATTAATACTTTCTTTGAACATATTAAAAATGCAGAAAATATTGGAGTATTTTATT
TTAAATAAATTGTATTCTGTATATTTAAGGTATACAACATGATGTTATGGGATACATATA
GGTGGTTAAAAGATTACTGCAGTGAAGCAAATTAACGTATCCCTCAACTCACATAGTTAC
CCATTTTTTTTTTGTTTTGGTGGCAAGAGGAGCTTAAAATCTCATTTAGTGTGAATCCCA
AATACAGCACAATTTTATTACCTATATACTTCATGTTGTACATTATATTTCTAGACTTGT
TCATCCTACATATCTGCTACTTTGTATCCTCTGAGCTACATCTCCCCATTTTCTCACTTG
CCCCCCAAGTAGTTTCTTAAAGTGTCTCATGTAAGAGGGCAGTAGCTTTCAGCTTAAACT
TTTTCTCTGTATGTAGTCGATTTCTTTGAGGTATACTTTTCTCTCCAGAATAGTTAGATG
TAGGTATACCACTTTGATGTTGACACTAGTTTACCTAGAACTTATCTTCTGTAAATCTGT
CTCTATTTCCATCTCTGTCTCCATCTTTGTCTCTATCTCTATCTGTCTATCTCTATCTAT
CTATCTATCTATCTATCTATCTATCTATCTATCTATCTATCTATCTAAAGCAAATTCATG
CCCTTCTCCTATTTATTGAATCGAGACCATAGACAGGGGTGAGAGAAAGAATTTGGCAGG
AATGGGGATGTGTATTATCTGTGGCATAAGGAAACTTTACAGAACTAGGTTCAAAAGTAT
ACTTTCTAGTTCTTTCCCATGGCTTTTCACTTTGATGTAGTCCTTATCAGGTAACTGAGG
TTTTATATAAGTCCCCTGATTCTTAGAACATGAAGGTGTAGTAGTCAAGGTTGGTCCCTT
GAAACCACAAATTTTGTGAAAAAAAATTAAGAAAATTGAATAATTTCCTCAGCAAATACA
TATTGATCATCTGTTATACAGCCATGAGAAGTGGTTCTGTTGCACACGTTTATTTTATCA
GATCCTAATCCCAAACCAGGCATAAAATGGAAACCATGAAGATAGGATGAAATAACTTCT
GAATGTTTGAATGTTTGAAAATAGTGTACTTAAAAATACCAGGTGGTTTTTGTTTGTTTT
TTGTTTTTTTCTTTTTTTGAGACAGGGTCTCACTCTGTCACCCAGGCTGGAGTGTAGTGG
TGCAATCTCATCTCATTGCAGTCTTGACCTCCCAGGCTCAGGTTATCTCCCACCTCAGCC
TCCCAAGTAGCTGGGACTACAGGCACATGCCACCACGCCCAGCTAATTTTTTGTATTTTT
TGTAGAGACGGGGTTTCACCCTGTTGCCCAGGCTGGTCTAGAACTCCTGGGCTTAAGCGA
TCCTCCCACCTCAGCCTCCCAAAGTGCTAGGATTACAGGCATGAGCCACCATGCCTGGCA
GAAAATACCAGGTTTTTAAGTATCAGCACTTACTCTTCAATCTTTTCTATTACTATGTTG
TGCTAAATGGTATTTTTTATTTAATTAGAGCAATGCTGTTCAATAGAACTTTCTTTGAGG
ATGGAAATCTTTTATGTTTCTGCTATGTGGTACAGAGCCACTAGTGACATGTGGCTTTTG

AGCGCTTGACACATCTTGTGCAACACAGGAACTGAATTTTTAAGTAATTTATATTGCCAC
ATGTGGCTACCGTATGGGACAGTGTAGTACTAGATGATCTGTAAGGGCTGTGCTTCATCA
GTGTCGTTTTTTAACTGACAAAAACCTTTAGTTTTTTTTTTAGTAATGTGTTTATTTAAA
AGAATTCATAAAATACAAGTAAACAAATTAACTTGTTACCTGAGCATATGTCCTTTCATA
CTTATTTTTTCTGCATACATATTTTGGAAAATGGAATATCTGCCCTTTTTTTTTATCTGA
GATACAGTCTACCTCTAAAAATACATGATTCTAACATTCTCACTTTTGTTGGCATTTGAT
CAGGGTATAGAAAAACAGTTAAAAGGACAGAGAATGGTTGAGAGATTATGATATGAAGAG
AAAATGTGATTGAGTGTGGTAGACTTGGGGCCTGCTTGAATGTTGAGAGAATGACTGTTT
TCCGATAAAAAAAAAAAGTCCATTCTAGGATCCTAAAAGAAGGGTCTGAAGTTCACTGCA
GAAAGCAAGCTACATAGTACTAAGCCACTAAGGGGACATGGAGCCCTTAGTAATTCCTAC
CTTAGTAATAGTCTCATCATGCCCTCTTGGGAACCCAGCCTTGTTGATTAGCCTCTCTGC
TTTCTCTCCTTATAGTTCAACCTCCCTGTTTGTTCCAAGCAGTTCTTTTCCTGCCCATTT
ATTATGCATTTCTATACAGCTTTCCTCCTCTTTTTCTATACCATGCTGCAGTTCTTATTG
CTACCTAGAGGTTTTCAAAATTCCTAGGGGCGGATAAGTAGGCATAAACAAAGTTCTTCC
CTATTATCCTTCCTATTTTTTCACCTAGACTGAAGAGGTAGACAAAATAGAAATAAAGAC
ATTAAGGGTATGTGTTTGTAGTCCCAAAGAGCTTCTCTGGCAATTTTATGTAGTTGACAG
TGACGCTCTGAGTTCAGGACAGATTGGACTCCTTGGCTGAGAGGAGTGAGGAGATAGGAC
GGTAGAGGAGAGGGTAGAGCAACTCTGGAGGAAGCTTTCCCCTCACCTTTGCCAGTCCTG
TTATCCTAGACTTAACCATAATTAAAGATGAGGGAGGCACTCAGTAAAGGGATCTAGTGG
GAAGCTTGTTCCAGACAGCCAAGGAGGGAGGTTCGCGCAGTTCCTTTGGCCACCCAGGTG
GGGTAATTGATCCATGTATGCCATTCATGTACAATGTAGGCACTTATACCTGTATTCCAA
TGTAGTGAACTATACCATTACTCTTAAATTAATATTCTTTATTAGCTTCCATGGTGGCTA
TAGGCCAGGCAAGAGAGTTAAGAAAAAATAAATAGCCAGGTATGGTGACTCAAGCCTGTA
ATCTCGGCACTTTAGGAGGCCGAGGCAGGAGGATCGCTTGAGTCCAGGAGTTCAAGACCA
GCCTGAGCAAAATAGTGAGATCCTGTCTCTATTTTTTAAAAAAGCCTTGGGGCAAACAGG
AGTATGGAGGTTTGGATGCTAATAGAACAGCAGTGTCTTACTGCTTGGAGTTCTCTTGTT
TCTTGTCCTATCACCGTAGCCTTTGGATCACAGCAATTTTTCCATGACTCCATACTTTTC
AGTTCTTGAATATTTTTTCCTTTATTCCTCTTGTCTCTGTAAAGACATCAACTGGAGTTG
GACTGTAATACCAGGTATCTCCAGAAGATGGCACTATTTAACAGATTTTATAAATAATTT
GATGTGAGTCACTGTCATCTGAAGCTTGTTGCCTTTTCTTTCTTTCTTCTTTCTTTTTTT
TCCCCATCAATTCTGTATGTTTGAAATGCTGGGTTTTAAGTTAGTTAGAATAAGGGATGT
CTGTAATTTCCCTAAATTGAGAAGTAATATGCAAAGGTTGATATCAGAAGTCATATGCTC
ACCTTGCAACACCAAATAATACTGGCCCATTTGTGATTTTTGAAAGTAACACTCCATAAT
AAATGGATGTATATATAGAAGCATAACAAAAATAGAAGCACATAAAAGTGAAAAGTCTCA
TAAACGCCATTGTCACTACTCATGTAATTGCTGTTACAAATTTGTTTAAATGTTGAATAA
AAATGGTGTCATAGGCAACACAGTGTTCCACTACTTGGTGTTTTTAATAGCATTATTCTG
TCTCAGTGTGCTTTGGATTATCAGGTGCTTTTTAATAGTTGCATGGTATTACATTGTGTA
GATGAACTTGATTAATTTAAATGGTTCCCTGTTAATGGACATGTTGGTTTGTTTTTGTGA
ACAACTGATACAGTGAACATTTATTTTTTAAATAAAAAAAAGAGAGACAGGGTCTTGCTG
TGTTTCTCGGGCTGGCCTTGAACTCCTGGGGTCAAGCGATCGTCTTGCCTCTGCCTCCCT
GGGATTACAGGCATGAAGCCACCGCACCCGGCCCAGTGAACACTCTTGAATGTATCTTTG
TATACTTGTCAGTGTTTTTGTAGCATTGATTCCCAGAAGTGGGATTACTGGATTAAGTGA
CATGCATGTTTGCAATTTTAACAGGTATTGCTATGTCATTTTCAAAAGAAGCTATGCCAA
TTAATACTCTCACCAACAAGAGTGCTTATTTCCCCTCAGCATATTATCAGGCTTAAGTTT
TGCCAGTATGGGTGGGAGAACAGTAGAATCACATTGTTTTAGTGTTTGTTTCTCAGATAG
ATATAATTTTACACCTTATAACCTTCTCTTCTATAAATTGTCTATTTGTGTTCATTCTCC
ATTTTCCTATGGGTTCTTATTGTTGGAGCCCAATATATAAAAGGGGGTATTTGTTACAGA
ACCTCTTCAGTTTTGGTTCATGTCATGCCTGGGTTTTTACCCTTTCTACGGATGTTAAAA
AAAATTCTCTATTTTCTTCCAGTCCACTTATGGCTTTATTTTTTACATTTAGATTTTAAT
CCGTCTGGAATTTATTTTTGTGTATGCTGTGAGGTAGGGACCATACTTTTATTTTTTCCC
AAATGGGTTACTAGTTGGCCAAACATCATTTATTGAATAATTCATCTTTTCCCTACTGAC
TCGAAATACCATCTTTATTGTATACTAAATCCTCATATAGTTCTGGGTCTGTTTCTGGGC
TCTACTTTGTTCATTTACTGTGCTGGTACTGCACCGTTGTAATTGCTGTGGCTTTGTGGT
ATGGTATGGCTTGCTCTCTGCTAGGGCAAGTCGAAGCTCTTTTGTTCACCTGCTCTTTCA
CCCAAATTTTCTGTCCTGAATCCAGCACAGCCAAATTATGGTCATTGTCACCACCAACTA
CAGTGGGTGTTGAGCATTTCCCATTGAATCTCCTGTAAGGGTTTTATTGGATTCTGTGAT
AGCAGTAAAATGGGAGCCTAAGAGGTATTCCTTAAAGGACTACTAATCAGACCTGGTTTC
CCAGATGATGCTGAAGATGACGGGGCCTGGGCTAGACTTTTGAGGGACATATCCTTGGGG
TTGGGTGTGATATAGACCAGCCCTTACAATTTGCTTGACTCATGGGAATCGTACAGGGCC
AGAACCAGACACCTGTCATGCTAATAACTTCCCTCACAATTCAGAAATCACTGTGATTGA
AGATGGGTGGCTGTTATAATACTACCCACTTAAAAATGGATGTAACCCATTTTTTAGGAC
TCTTAAAAACATCAAATCAGTAATGGCCGATTAGGACTTTTTAATTTTTACTAATCTCTA
CTTGAAAGTTTTCTAGTCATTCATTTCAGGAAACCTAATTCTTATAATTCATATCATTTA
GAATATCATAATGCTATGGATATTAGCTAGCTAACTTCTCAAATCTTCTAGTTCTCATTT
AATTTGAAGTTTGTGTGTGTACATAAGGATATACATATACATATGTGTGTGTAGATATAT

ATATATATAGTTTTTTTTTTTTTTAACTAG**AATGACCAGTCAACAGGGGACATAAAAGTA**
ATTGGTGGAGATGATCTCTCAACTTTAACTGGAAAGGTATGTATCTTGAAAGGGAAGAAA
AAAAAGCACTTCATACCGAGTCAATTAGTAACAGTGTGCTTTCAATCAATCACTAAGAGA
TAATTTACATAGTATAACTAAATGGGTTATTTAACCCTTGGAAGCAGTCTAGGTTAATTA
TCGTTCCCTAGGTCATGTAGTAAAAAGACAGTAGAATCCAACATTAACCTTAAATGTCCA
TATTGTCAAGTACTGCTGTCTGCCTCTGTGGGACTCTAATTTGGGATCCTTCAAAAAACA
TTGATGGGGGAAAAGATAGCCTTTAAAAAAAAAAAAAAAACAAACCTATGTGAGTCTATG
TGAGGTAGACTCACATAGTTTCCTAAAAGATAGCAAAGCAGTATTATGTAGTGGCTGAAA
GTGTGAGTTCCGGAGCCTGACAACTGATTCAAAGCATGGCTTAGTACTTCCTAACTCTGA
CCTTGGGCAAGTTACTTAACCTCTCTGTGTCCCATATGTGATTAGGGTGAGGTTGATAAT
AGCAGCCATAGAGTTAAGAGGATTAAGTGCTATAATGCAAGTAGAGCTCTTACAACAGTT
TCTGGTAAATCACTCAATAAATTCAGACATACTATTATTTTAAGAAATCTCAAAGAGTTT
TCTTGTACCTTAAAATTCTCCTAGTGTGAACCATTGGTTTTGGTATATTGTGCTTCCATG
TAGTTTAATATCAAGATGTTTTTAGATTTCCCTTTTAATTTATTTGTTGACCCATTGGTT
GTTCAGGAGCATGCTGTTTACCTGAAAATAATGGAGATATTAAGGTATTTGAATATTTAT
CTTCTAGTACATTGAAAAACTTTTTGAGAGTAACCAATAATAAATGATGGAATGCTACTG
CTTTTTTTTTTTGAAGCTGCCAGTTATTGTTTACTTACACTATGCCAAATATAAAGGCAT
TAATCTCATAAAAGTTTCACAACAATCCTGTGAGGGAGACGATATCCCCATTTTACAAAT
CAGGAAATTAAGACTTAATAAGGTTAAAAGACTTGCCCAAAGTCACAGAACCAGTAAGTG
GTAGAGCTTGAATTTGAATACAGACCTGACTCTAAAGCTCTTTTCTTTCTTTAGATTTTA
GTGTTCATTGCTTACTTGAATGAGTATCTATAAGAAAACTTTAACATGTAAAACTTCTGT
GAAATTATCTTGTCCCATATCAGGGTCATGTCAAACTAATGTCCTCCTCAGCATCTTTGG
AAAACTTCAGAGGAGAAATGAGCTTTGCCCCTCCTGTTCATTTCATATACCACTGTTAGA
CCTGTCCTTCCCTTTCAGCATGCTTTGTCCATATTTAGAAGCTGTTGAAGCCATTACTTG
TCTGGTCAGTTTTTAGTGCTGGAATGGACCTAGCCTTTTAGGCCTTCTGAGATTTAGTTT
GATCTCGTCTTTCCCACCTAATGGCTCTGTTCTACTACATAGATTTGATCTGAAACAGTT
CTCTGTTTCTAAAATAACTTTCTTTTCATGATAGTCACAGTAAAGTACATTTATTATGGA
AAAATCAATAAGTATAACGAGTGAAAGTTATTTCTTGGTGGTAAGATTATGGATTATTT
GAACTTTCTGTTTCATTGTATTTTATTTATTTATTTATTTTTGTGATGGAGTCTCACTCT
GCTGCCCAGGCTGGAGTGCAGTAGTACGATCTTGGCTCACTGCAACCTCCCCTTCCCAGT
TCAAGTGATTCTCCTGCCTCAGACTCCCAAGTAGCTGGGATTACAGGCGCACGCCACCAT
GCCTGGCTAATTTTTTTATCTTTAGTAGAGACAGGGTTTCACCATGTTGACCAGGCTGAT
CTCCAACTCCTGATCTCAGGTATCCACCTGCCTCAGCCTCCCAAAGTACCGGGATTACGG
GTGTGAGCCACCCTGCCTGGCCTCATTTTGTCTTTTGGGGGTATTTTTGTGTGCAGATAT
ATATGTATATAAATATTTTTCCCTCTTTTCCCCAGTTAGTATTTGAGCAGATGAACTTTG
GACCCGAATACCTGTATTCAAGTCTCTAATACCACTTCTTGGCTATTTTCATTTTATCAA
ATGGCCTCTTATCCTCGTTTTTCTCATTTATTAAGTAGAGATGTAACTACTTGATATAAT
TCAAAAACTCAATAATGGCATTCTTTTGTTTTTTAGACTCTAGTGTCTGTACTCGTTGTA
CCATGCTGGGATTCATTTGAACAATTGCATGGCTTTTTTAGTGTATTATTAAATTTGCAG
TTTACTTAGAATTTACTGGGACCTCATACAAATGGGAAAAAAACATAACTGTGTTACTCA
TTTGCTGTGTGCCTTTGGATTGACCCTATTTTTTGTATTCATTTTCTCCCCATGTCCTGA
GTTCCACTTTGAATAAAAAGTAATTTTTTTCCTGCCTGTAAAATAGGCTACCAATAGGCT
GCAGTTGTCTATAGTAGCTGCTTCACTGAGGAGAGCTCAGCATGAGAGAAATAGTATGAA
TTGCTTGCCACAAGTTATGGGCTAGCCTTACTTCATTCTGTACTTGGACCTGTTTAGGCT
TCTAAGAGATCTTACCTCCAACAATAAACTGCTTTGAGACATGAAAAGGTGGAAGCTTTA
CTTGGTTATAACTTTACTTTTAATACCTAGAACAGTGAGTCTTCAAACTTGTATTTGCAT
GCCCAATTTATAAAAAGTTTCCTGAGCATTTACCCCTAATATATGCATTTTAAATTATAT
ATGATTTATGGTAATAATAATATATATGTTACAAAATACATACAAAAATATAGATTAAAC
AAGGTGAGGTAAAAAATTTAAAAGTTCTAATCTTTCTTGCAAACCAGTGGATCTTTTGTG
CCTTACTCTGGTAAACACTGTCTTAGAAGAATATATAGAACATTAAAATCTTAATGCTAT
AGTTATATGACAGAGTATGATGAGAGCTACAGATAAACAACACATCATGAATCTTCTTGT
GGCAGTGTTTATAACCATTATGTGAAATGCTGCCTCATTCTTATAACTAGCATAAGAACA
GATAGGACTTTCTCGATTTTGAGGGGTAATTATTAGATGGTATTTTCTGTTAAGGACTCT
TCCAGCTATAAAATTCTTAAATGTAGAAAGCGAAGTGAGGGTTTATGGTGAGAGGAAGCA
TTGGTATCATGTTTTAGTGTAGTCCAAGAATATGGACACATCCAGAAAATGCAGATCAAG
TTTAGCCTAATGAGAAAATATATTTTGGAGTCCATATGGTAAATTAAATTATGTGATTTT
TGAGTTATTGTACAAATATAATTCTTAGAATGTTAGAGTCAGGAGACTATAAGAGACCAA
CTGCTTCAAGTTTCATTTAACACATGGGAAACTAAGGCCAGAGAAATTTCAAGACTTGCC
CAAGATTAGACCTCTTGTTAAGTAATGAAAGTGTTTTAAAAACAGGTGGGTCAAATTCTG
TTTTTAAAATTTCCATTATGATGAAAATTTCAGTATTACAGGCTTCCAAATCCCAGCAGA
TGGGCCACTTGTTTAAAGGAGAGTTTGATATAATAAAGCATCTAAAAACAAGAGTTTGGA
TAATTCCTTAGGGTTGTTATGATGTGATTTGACTTATAATTGGAAATACCGTTTTATTCA
TTGTACTGATTTTCATTTCTCTTTTTCTTCTAG**AATGTCTTGATTGTGGAA**GTAAGTTCA
CATTTACTTTTAATATAACATTTATGACTTTTCTAACTTAGTATGCACCATCCTAAAGGT
AAGCCAGGGAGAGAAATTCCTCTGCATCAGTTTTAATGGTGGCTTGTGTTCTAAAGGAG

TGAGATTGGTTTTTTGTAAAGACTACTTAGTAATTTGTTTTTACCAATAATGGAATGGTA
TACTTCCTACCTCTCTTTTTTTAGTTTGAAGTATTTTCTTTCTAAACATAACTCTCTCTC
TCTATTTATCTATATATAATATATACATATATATCTTATATTTTATGTATATATATATAT
ATCTTGCTTAGATTTTGTCTTATGTAATATTTGGTACATAAAAAATAATATTTATAATTT
ATAGACTATTTTCCATGTGTTATTATGTGCTAAAGTATTTTGTATCTTAGCACCGAGAGG
CTAAGCAGTTTCCTAGGGTTACCAGCTAGTAAACTAAGGGAAACCTTTACTTCCTTTAGC
TCAGTGGTTCTCAAAATGTGGTTCCCTAGACCAAAAGTATTAATATCAGACAAGAACCTA
CCGAATCAAAATATCTGTGATGAGGCCCAGCAAGCTATGCTTTAACAAGTTTCCGAGTGA
TTCTGATGCATGCTAAGGTTTAGGATCCCTTGTTTTTACTCATAAGTCACTTTCTCATTA
AGGCCTTCCCTGGCCATCCTATATAAAATCTCATGTTTTCACACCGTCAACTTCGTATTC
CTCCTCAATACTTTTATTTTCCTGATCACTTATCACTAACAGCCTCTCTCTCTCTCTCTC
TCTCTCTCTATGTATATATATATATATATCACTTATCACTGTCTAACAGCCTCTCTTTAT
ATATATATAATCTATAGATTATATATATATGCAGCATTGTGCAATCATTATCACGCTCAA
TTTTAAAACATTTTCATTGCCCCACAAAGAAACCCAATCCCCTTAGCCATCACTCCCCAT
TTCCCCTTCCCCCAGCACCTAGCAAACTGATCATCTACCTACTTGCTGTCTATAAGATTT
GCCTATTCTGGACATTTTGTATAAATAGAATCATACAATATGTGGCCTTTTGTATCTGGC
TTCTCTCACTTAATGTTTTCAAGGTTCATTCATGTTGTGGAGTATATCTGCACTCATTTC
CTTTTTATTGCCAAATTGTATGGATAGACAGGTGTTCCTCAACTGTGTCCTGATAAACCC
ATCTGAAGTTGAAAATATCATAAGTTGAAAATGGATTTACTACTTTGATAAATCTATCCT
AAAGTCAGAAAAATCTCATGTTGAACCATCGTAAGTTGGATACCATCTGAATTACATTTT
TGTTATCCATTCACTGGTTGACAGACGTTAGGTTGTTTCCACTGTTTGCTCCTTATTTCT
CGTACCTGAAATGTCCTTATTCCCTCCCTTCTTATCCCATGTTTAAGTCATTTAAGACCC
AGCTCAAACGTCACCTCCACAAAACCTTCCTTGATACCCCTTTCCTCTTCAATTCACTTG
GACCTTTTGCATTTAATTTTAATTTTTATTTTTTTTAAGACAGAGTCTCACTCTGTCACC
AGGCTGGAGTGCAGTGGTATGATCTCAGCTCACTAACTACTCTGCCTCCCAGGTTCAAGC
AATTCTCATGTCTCAGCCTCCCAAGTAGCTGGGACTACAGGTGTGCGCCACCATGCCTGG
CTAATTGTGTGTGTGTGTGTGTGTATGTATGTATGTATATATGTGTGTGTGTGTATATAT
ATATATACACACACATATATAAATATATATACATATATATATATACACACATATATAAAT
ATATATACATATATATATATACACACACACACACATATATATATATATAGTTTTTTTTTT
TTTTAAGTAGAGATGGGGTTTTGCCATGTTGGCCAGGCTGGTCTGGCCTCAAGCCATCCT
CCCACCTCGGCCTCGCAAAGTGCTGGTATTATAGGCATGAGCCACTGTGCCTGGCCTGCA
TTTCATTTTAATTATAAAATATTTTGAACTCAGAAAAAAGGGTATGCTGAATACCTACGT
ACCCACAAAAGTATTAACATTTTGCCATATTTGCTTCTGATCTTATTTTTTTTGAGAAAT
TAAAGATCATAATACAACTAAAGCCCCATTTCTTTCCCTTCATTCCCAGAAGTATGACAA
TTATCCTTAAAGTTGATATATATCATTCCCATGCATGTTTTTTATACTTCCCTAGTACAA
GTTAGCTGTATCCTCTGCTCAGGGGCTCATCAAGCTGAATCAAGGGACTCATGATCCTCT
TCAAAGTTCCTTCAGGTTGTTGGCAGAATTTAGTTCCTTGTGATTGTAGGACTGAGGGCC
CGTTTTCTCACTGGCTGCTGGCCAGGGGTTGCTCCCAGATATTTAAAGGCTCATGCCCTA
GCCCATGACAGTCTCACAACATGGCAGCTGACTTCTTCAAAACCAGCAGGAGAATCTTGC
TCTAGTCTACCACATAACCTAATCACAGGAGCGGCTATCCCGTTATTTTCACAGATCCTG
GTCACATTCAAGGGGAGGGAACCCTTCTGTGTGTGTACACCAGGAGGCAGGAATTTTTTT
TTTCTTTTTCTTTTTTGTTAAAAAGTCTTAAAGTCTTTTATCCCTAAAGGAGGCAGGAAT
TTTGAGAGCCATCAGAATTCTGCCTACCACAGCCCAGAAATCTGCATTTTTCACAAGTCT
CCAGCCATGATGTTTCTGATGGCTCACACTGCTTTATTCCATTTTTAAAGAGTATTTTTA
TTGAAAAGCATTAGGGTTATGGTTTAAAAAATATTTTCCCTAACAAAGATGGGTTTGTTT
AGAGTCCTACTTTTGACTAAATAGCTGAGATTCACTTTTATGTAAAGTCATTTTATAGCG
TTATTAATTTGGGTGCCTTTAAAAATAGTATAAAGCATGTTTCTCGAGTGTAGTCTGTTA
GCCACCTATATTGGAGAGTTGGGAGGAGAGAGTCTCTATCTTGAATTTATGGGAAAAATT
CTAAAATACTTTTTATAATGAAGGACAACATCATAACTCCCTAATAAAATGTGCATGTAT
ATATTCAAATTTGCTGTCATTGATCCTGCACCTACAAAATCCAGTCCTGGGGGCTGGCAT
TCTTACTGCTTGCTGAGGGCCAGATGATATAGATTCCAGAATATCTCCATGTAGATTTTG
GTGAGAATTACTGTGCTGAAAAGAATGACAGTATTGCAGTTATACATGGGGGTTTTGGTA
CTTTATATTGTGACTCTGAATTTAAAGCTATGCAATGTCTTCTTTTTTGAAAG**GATATAA**
TTGACACTGGCAAAACAATGCAGACTTTGCTTTCCTTGGTCAGGCAGTATAATCCAAAGA
TGGTCAAGGTCGCAAGGTATGTATGACATTTTGACACAGAATATTTTCCTCATTTGAAGG
GGGATTAAGTGATTGCTTCTTTTTAAGGATAAATGTTTTCAACTGTCATTTTATCTTCGA
AAAGTAATGTAATCTCATATAAGACTTAAGATATAATCCTTTTAAATAATTTTGTCATGT
GTTAATAAAGCTCATAATTACAGTCACTTCCTTGCCTAATATTAACATTTGGTTTTTCAG
CATGCTAATTATATCAGTTTGTCCTGAATAGCATGGCAGAGGATTTTGGGCCCCCTTGCA
AAATTAAGAATAAGGATTCCAAAGCGGGTGAGGAAGTGATAGGAAGGGGTGGGCCCTGAA
GATCTGGACCTCCTGGAATTGAGTGATGAATGCTGCATCTTCTTTGTGTCTGTAGTGAAA
TTTTATAATGCCTGCTTCCTTTTTTATTAAGTCGGCCTCACCTCCTCACCTTACCTATGC
TGTTTTACTTTTGCTTTTATAGTTCTACCTGTGTTTATTTCTCATTTTCGTTTCATCTCT
CAACAACTCTGGGGTGGCATTATTATTCCCACTTTTCAGATAAGGTTACTGAGGCATAGG
GAATTGTCCAAAGGTACAGAGCTAGTCCGCTATAGAGATGAGATTTGAACCCAGGGAACC

TGGCTCACAGTTTATGCTTTTGCTACCTTAAGTTTTTAATAGAGTGACATCAAACAAACA
TTTAAGAATATGTTTTTCTTTTCCTTTTATAATTTCATTAAAAACATTAAGTCTCTGATC
AGTCTGCAGTTTTTATGTAGGGGTCAGGTAATGTTCTAACTTCTGCTTTTTCCTAAGTGA
TTAACAGGTTTTTATAAGCCCTTTTGAAAAAATCACGGTATCTGTCGAGCATCTTTGAAT
CAGAGTAAGCCTTCTAGTGAGTCATATGTCAGCAGTTTGACTGTATGGGCTTTTCTAATA
TCCAGTTCAAGTGTTTATCAGTGAGTTTTTCTTTTAAATAGATTTGGGACAGGTACTATG
AGAGTATATAAGTGATACGTTATAGGACACTAACTAGTATCCTATGAAATGGCAAAAACT
GCAATCACTTTTGCACCAACCAAATAGAAACTAATCAGTGCACTTGCTTATTTTTCTACA
TGCTCTTTAGGGTTTAAATGTCAACCTACTGTGGCATAGACTTTAATCCTCTGGGTATT
CTTTTGTTGTTCTTTCCTGGTATATGCTGTGGAATTGAGATAG**ACTGGTTCGTGAGCGAG**
AGATTTTGTGTTGCCACAGGTAGGACATGCTCAAACAATACTTGGGTCATTTCTTGACCC
AAGTCATCTATTCACCATAGTTTTGTAGCACCGATCTTGCATACATTTCATGTATCTTCT
TTGAACCCCACGTCAGTGCTGCTTATATGATACTCAGAAATTAAACACTAAGGAATAAGA
TTTTCAGGTAGGATTGAGTTTTGGAGGGTCACAAATCTTGTAATGTCTAATATTTCCACT
CTCCCTGCTGAGAATTAGTTTTGGCTTCCTTGGAGGTGATATCGCCTCTGTTGAGTATAA
GTGGCCTACTGTGATCACACCACTGCACTCCAGCCTGGGTGACAGAGTGAGACCCTGTCT
CAGAAAAAAAAAAAAAAAAAAAAGAATGCATGGCCTAGATGACTTCTAAGGTTTTTCCCAC
CCAGTTCCAGTTTTCATGTTCTAGGCAGAGCAGTAAAGTGAGAAACACATGGACTTGGGA
GTTTAGTCTCGCATTTCACTGCCACTTAATCTGAGCGACTATTCCATATTTAATCTCTCT
GAATGTATTTACTCATCTTTAAAGGGGAATGATTATTAACATCTTTTTCTCAGGGAAACT
ATATGAGTCAAGGAGATAATATATTTGAAAATCTTTTTAACTGCAAAGCGCTGTTTCACT
GTTGGTTATAATGTGATTGATCTCATTGTAGTGAGCAGCTGCTTAATTGCGTTTTAGAAT
GTAGGGAAGATAGTAATATTTTTCACATTATATATGTAGCTGGTTCTGGAACTGTAAACA
TACTCCTTTTTTATGGAGATCTGAGTCACGTACCATAAAATTCACTCTTTTAAAGTTGTA
CAATCCAGTGGTTTTTGATATATTCAGAGTTGTGCATCTGCTACCACTATTTCATTTTGG
AACCCAAAGAAACCTTGTACCCATTAGCAGTCATTCTCCCTTCTCCCAGCCCCTGGCAAC
TACTAATCTACTTTCTACAGAAAGTCCGTACAGATTTGTGTATTATGGACATTCCATATA
AATGGACTCATGCAATATCCTGTCTTCTTTCACTTAGCATAGTGTTTTCAAGGTTCATCT
AGGTTGGGGCATGTATCAGTACTTCATCCCTTGTTTTGGCTGAATAATATTTCATTGTAC
AAATATATCACATTTTGCTTATCCATCTGTTGGTGAACATTTGAGTTTCTACCTGTTGGC
TTTTATGAATAATGTTGATTTGAATGTTTGTGTACAAGTATGAATACCTGTTTTCAGGTC
TCTTGAGTATATAGTTGCTAGGTCATATAGTAACTCTGTGTTTAACATTTTGAGGAATTG
CCCGACTATTTAACAAGGTATATGTACTGTTTTACACCAGTAACATATGAGGGTTCCAAT
ATCTCCACATCCTTGACAACACTTGTTACTGTCCTTTTTATTGTAGCCATCCTAGTGGCT
ATGATGTGGTATCTCATTGTGGTTTTGATTTGTGTTTCTCTGATGCTGATGATGTTGAAC
ATGTTTTCATCTGCTTATTGGCCATTTACATATATCTTCTTAAGAACGGTTACCCATTTA
CAGTATGGAAAATGCTTCAGATGCAACTCTAGTCATGCCTTAGAGATGGAGCTTTATTAA
ACATTCAGATCTCTAGGCATATGAAGTGCTGAGTTCTCTTGAACTCCTAATACAGATTGC
ACTGAGTTTAGTGATACCTTTTCTGGAGCATTCCTGAGTTCAGGTAGGGAGAAGGGTTTT
TGCTGTGATTGGCTTGTTATGTTCTTTCTAAATGGAAATAGAATTGAAGTGTCTCCTCTC
TCCATTTATTGGAAGAGTCATGAGGGACATAATTAGATGATCCCTTGGAGTCTCCGGCTT
AGGTCAGTGGTTATCTACTTAGGCTGCACATTGGAATCACCTGAGAGTTAAAAAACCAGG
ATAACCTCTGCCTGTGTCTCATCTCCAGCAATTCTGATGTAATTGGTCAGGGCTGTGGCC
CGAGTAGGTGAGTTCTGGTTTTTTAAAGCTCCCAGGTGATTCTGATGTGCAATCCAGGTT
GAGATCACTTTGGGCCCTTTCCAGCTCTTTAAACATATATATTTATCTAGGAAGGTATGA
AAGCATAAGTTTTCTTGAGACTGCCTTTAACATCTGTAAAGGCTTTCAAAGCAGCTTCTG
TAGTTTTTTTTAAATGGCTGAATATTTTTCAACAGGCAGCATTTGGGTTATAAAATTAGC
TTTTGGTAGAGTTGACTTATACCACCTCCAGCTTTTGTTCCAAAAATAAATACTGGTTCT
TTTGGCACACTAGTTGTTTTACCCTAAAGTTCCTCTTTGTAAGCCAGTTATTAAAAGTTG
TGATGCAGCCAGGGCGAAGTGGTACACATCTGTAGTCCCAGCTACTCGGAAGGCTGAGGG
GGGAGGATCGCTAGAGCCCAAGAAGTCAAGGCTGCAGTGAACTGTGATTACACCACTGCA
CTGCAGCCTGGGCCACAGAGCGAGACTCATCTCTTTAAAAAAAAGAATGTTGTGAGGCCG
GGCGCAGTGGCTCACGCCTGTGATCCCAGCACTTTGGGAGGCCGAGGTGGACGGATCACC
TGAGGTTGGGAGTTCGAGACCAGCCTGACCAACATGGAGAAACCCTGTCTCTACTAAAAA
AAATACAAAATTAGCCGGGCGTGGTGGCACATGCCTGTAGTCCCAGCTACTCGGCAGGCT
GAGGCAGGAGAATCGCTTGAACCTGGGAGGCAGAGGTTGTGGTGAGTTGGGCGAGCCATT
GCACTCCAGCCTGGGCAACAAGAGCAAAACTCCATCTCAAAAAAAAGAAAAGAAAAGAAA
AGAATGTTGTGGCCAGGCGCGGTGGCTTACGCCTGTAATTTCAGCACTTTGGGAGACCGA
GGTGGGCGGATCACGAGGTCAGGAGATCAAGACCATCCTGGCTAACACAGTAAAACCCCA
TCTCTACTAAATACAAAAAAAAATTAGCCGGGAGTGCTGGCGGGTGCCTGTAGTCCCAGC
TACTCAGGAGGCTGAGGCGGGAGAATGGCGTGAACCCAGGAGGCAGAGCTTGCAGTGAGC
GGAGATCGCGCCACTGCACTCCAGCCTGGGCAACAGAGCGAGATTCCGTCTAAAAAAAAA
AAAAAAGAATGTTGTGATAAAAGGTGATGCTCACCTCTCCCACACCCTTTTATAGTTTAG
GGATTGTATTTCCAAGGTTTCTAGACTGAGAGCCCTTTTCATCTTTGCTCATTGACACTC
TGTACCCATTAATCCTCCTTATTAGCTCCCCTTCAATGGACACATGGGTAGTCAGGGTGC

AGGTCTCAGAACTGTCCTTCAGGTTCCAGGTGATCAACCAAGTGCCTTGTCTGTAGTGTC
AACTCATTGCTGCCCCTTCCTAGTAATCCCCATAATTTAGCTCTCCATTTCATAGTCTTT
CCTTGGGTGTGTTAAAAGTGACCATGGTACACTCAGCACGGATGAAATGAAACAGTGTTT
AGAAACGTCAGTCTTCTCTTTTGTAATGCCCTGTAGTCTCTCTGTATGTTATATGTCACA
TTTTGTAATTAACAG**CTTGCTGGTGAAAAGGACCCCACGAAGTGTTGGATATAAGCCAGA**
CTGTAAGTGAATTACTTTTTTTGTCAATCATTTAACCATCTTTAACCTAAAAGAGTTTTA
TGTGAAATGGCTTATAATTGCTTAGAGAATATTTGTAGAGAGGCACATTTGCCAGTATTA
GATTTAAAAGTGATGTTTTCTTTATCTAAATGATGAATTATGATTCTTTTTAG**TTGTTGG**
ATTTGAAATTCCAGACAAGTTTGTTGTAGGATATGCCCTTGACTATAATGAATACTTCAG
GGATTTGAATGTAAGTAATTGCTTCTTTTTCTCACTCATTTTTCAAAACACGCATAAAAA
TTTAGGAAAGAGAATTGTTTTCTCCTTCCAGCACCTCATAATTTGAACAGACTGATGGTT
CCCATTAGTCACATAAAGCTGTAGTCTAGTACAGACGTCCTTAGAACTGGAACCTGGCCA
GGCTAGGGTGACACTTCTTGTTGGCTGAAATAGTTGAACAGCTTTAATATACAATAATTG
TTGCATTATTATTTCAGATGATAAATGTGGTCATAAGTAAGAAATAAATGATCGAGTTTA
GTCTTTTAATTCACTGTCCTTTGAATACCTGCCTCTTACTCTGGAGGCAGAAGTCCCATG
GATGTGTTTATGAACATGGTTGAGGAAGATTTAGGAAGACTGCAACAGTACACTACCTAA
AGCAGGTTTTTACTCCATCTTTTTTTGCCACGTACACTGGCCTCCCACTTTGATATGCT
TGAAATTATCTCCTTGATTTGTCTTTCAAAACTACATATTGAGGCTGGTTGCGGTGGCTC
ACACCTGTAATCCTAGCACTTTGGGAGGCCAAGCCGGACAGATCACTTGAGGTCAGGAGT
TCGAGACCAGCCTGGCAAACATGATGAAACCCCACCTTTACTAAAAATACAAAAATTAGC
CAGGCGTAGTGGTGTGTGCCTGTAACCCAGCTACCTGGGAGGCTGAGGCAGGAGAATCAC
TGGAACCCGGGAGGCAGAGGCTACAGTGAGCCAACATCACGCCACTGCACTCCAGCCTGG
GTGACAGAGCAAGACTCTGTCTCAAAACAAAACAAAAAACAAAAAACTACGTATTAAGAC
AAGAAACAGACTGGGCGCGGTGGCTCACGCCTGTAATCCCAGCACTTTGGGAGGCTGAGG
CGGGCGGATCACAAGGTCAGGAGATCGAGACCATCCTGGCTAACACGGTGAAACCCCGTC
TCTACTAAAAAATAGAAAAAATTAGCTGGGGTGGTGGCGGGCGCCTATAGTCTCAGCTAC
TCGGGAGGCTGAGGCAGGAGAATGGCGTGAACCCGGGAGGCAGAGCTTGCAGTGAGCAGA
GATCGTGCCACTGCACTCCAGTCTGGGTGACAGAGCAAGACTCCGTCTCAAAAAAAAAAA
CAAAAACAAGAAACAAATTAAACTAATGTGATAGACTACTGCTTTGTTTTCAAAAGATAC
ACTCCCCAAAAGTTACTGATCTAAAATACAGTAGTACTATCTCTGTTTAGTAAGAACCCT
GACAACTAATAGTGTTCTTATATGTAAAATGCTATTCTTGCCTTTCATTTCAGAATATAC
TTTTTAAATGTGAATTTCTGGATTTTTTTTTATAG**CATGTTTGTGTCATTAGTGAAACTG**
GAAAAGCAAAATACAAAGCCTAAGATGAGAGTTCAAGTTGAGTTTGGAAACATCTGGAGT
CCTATTGACATCGCCAGTAAAATTATCAATGTTCTAGTTCTGTGGCCATCTGCTTAGTAG
AGCTTTTTGCATGTATCTTCTAAGAATTTTATCTGTTTTGTACTTTAGAAATGTCAGTTG
CTGCATTCCTAAACTGTTTATTTGCACTATGAGCCTATAGACTATCAGTTCCCTTTGGGC
GGATTGTTGTTTAACTTGTAAATGAAAAAATTCTCTTAAACCACAGCACTATTGAGTGAA
ACATTGAACTCATATCTGTAAGAAATAAAGAGAAGATATATTAGTTTTTTAATTGGTATT
TTAATTTTTATATATGCAGGAAAGAATAGAAGTGATTGAATATTGTTAATTATACCACCG
TGTGTTAGAAAAGTAAGAAGCAGTCAATTTTCACATCAAAGACAGCATCTAAGAAGTTTT
GTTCTGTCCTGGAATTATTTTAGTAGTGTTTCAGTAATGTTGACTGTATTTTCCAACTTG
TTCAAATTATTACCAGTGAATCTTTGTCAGCAGTTCCCTTTTAAATGCAAATCAATAAAT
TCCCAAAAATTTAACTGCTTTATGAATTCAATTTAAAAATCCTTAAAATAAGTCCTGTCT
CTTTAAAAAAACCTATGCATAGTTATCATTTCTCTACAAATTAACCTAGTTTAGTTTTCT
GTTGGTTCCATTTTCCTTGTTTGTTAAGTTTTAGTAGCTAGTTTAATTGTAATCTCAATG
ATTATGTGGTAGAATGGGTTGGCGGAGCTACAAAAATTCCTAGCTACTTCAGAGACATTA
AATTTCAGACACATGGTACACTTTATATTACATTTTACTATGCTAAAATAACACGGCTTT
CTTTTGGAATTCTGTTCAGTTTTTCAGATTGTAATCTCAGCTACATCTCAACAGATTGTT
CTCAGATATGTCCTATTACCTTCTTTGTGTAGATAGTGCTTTATTGACTAAGAACAATGA
CAACAACACCTTTTGTTTTCTGGGAATAGGAGAAAAGTTTTAAGCCAAAACTCTTAATTG
CTTATCTGCTCCACGTGAGGTATGAACTATCAAACTTAGGAGCCATCTAGCTTACACGTG
TTCCTTAAAAAGTTTGCTGTAGGCCGGGCACAGTGGCTCGTACCTGTAGTCCCAGCACTT
TTGGGAGCCCAGGGTGGGGGATCACTTGAGCTCAGGAGTTCAAGACCAGCCTGGGCAACA
TGGCAAAACGCCATCTCTACAAAAATACAAAAAAAAAAAAAAACGCTGGGTGTGGTGGCG
CACAACTGTAGTCCCAGCTACTTGGGAGGCTGAGGTGGGAGGATTGCTTGAGCTTGGGAG
GTGAAGGCTGCAGTGAGCCTTGACAGTGCCACTACACTCCAGCCTGGATGACAGAGTGAG
ACCCTGTCTCAAAAAAAAAGAGTTTGCTGTAATTCCCAGCAACAAAGTAGGAGACTCAAAC
TAAATAATTTTCTATAGTCCTAGAACTTCTTAGTTTACAAAACATTTTTACTTCTGTTAT
CTCATTTGATCTTCATACCCATGTAAGGTTGAGGTAGATGTTACCACATGTGAGTGCAAT
ATCCAGAACTCCTGAATCCCCTTCTTCCCCTAAAATGTCAGCCCGCTGAGGTCCACTTGG
CTACCCTCTTGAATACTGCATCCAGCTTCCCACTGCTGAACCTCTTTACTCTTTTTTTTT
CAGTTGCACTTACCACCTTCTAGTAAGTTGAACCATATGAAATTACCATTTTTGCAGGTA
AAAAATGGCCGGTGATAGGCAGTTTGGCGTCGTATAACCCAATAACATGTTATATAATTT
ACCCACAAGTGGTGGGTTGCTATGTCCTGGAGGAGTCAGCTTCAGACTCTAGCTAAATGA
TTGTATAACCTTGCAGCTCTCCCCTAAGTGAGGAGGCAATGTTGAAAGTCCCATGTCTTA

TCAGAACCAGGGAGGCAGATGAGAAACTGCCTTATGGCAGCTCCCACAACATAGGGAGGT
GGGTGACAAATGGCCTTGGGACAGCTTCTTCCCAAGACTGGTTATGTTACAGTGTTCCTG
GGAGGATCACATGGCATTCCTCCAAGATGGGTCAGACTGCTGTTGGCCTTGTCTGTGTGG
CGTATGTGAAGACATTCATGGCAGAGCTGTTCCCTTAGAAGCATCTACTAAATTGATCTT
TTCCTTTCTTACTTACTGTCTGTCTCCCTTAGTAGGCTGTCAGCTCCGTGAGTGCAGGAC
CTTGCCAGTCCTGGTCACTGCTATATCCCCAGCACCTACAAGAGTGCCTGGAAAATTGTA
GTGCTCAATAAATATTTGTTGGATAAATGATAGAATGATAGGAAGTTAAAAAGCAATTAA
AATACTTGAAAAGAAGCAAAACATTTTTCATGTTAAGCAAAAAAAAAAAAAAAAACTTATT
AAGGATAGCTAACATGTATTGAATTCTATATGCAATGGAATGATACTTAGCGCCTTTGAA
TCCTTATGATAACCCTATAAGGTAGGTTGTTTGGGTTTTTTTAATTGTCCCAACTTTACA
GATGAAGAAGTGCAGGTCCAGAGAGGTCACATAATTTGCCCAGGATCACACAGCTAGTAA
GTAGCAGATGAGGAATTTGAACCCAGGCAGTTGTATTCCACCATCTGCCCTCTTAGTTCA
TTGCCACTTAACCTATAATGCCCAGCTCTTGTGTAGAAATTAATACACTGATAACATAGA
GGAAAACATTAAGCTCATTGAATGTAATAAGTCCAGATGACTTGTACATTAAACACAGCT
TTTTGAGGTCACAGCTGATCTCTAAGAATGTAAACTGATTTCCTCTGGCACTAAAAAGCA
TTTTCAAAGACTGTTAAGAGAGTTTCTCCAACATTCTCTTCAGATTTTTCTGCTGGCTTA
TTTTATGATTCTGTGGACAGCTTCAGACAAAATAACTTTCTGGTATGAAGGATTGTGTTT
ACTCTGCTTTTTTTTTTGTTGTTTTTTGGGTTTTTTGTTTTGTTTTGTTTTGTTTTTGAG
ACAGTGCCTTGCTCTGCTGCTGCCCAGGCTGAGTGCAATGGCATGATCTCGGCTCACTGC
AACCTCTGCCTCCCTGGGCTCAGGCCAGGTGTATGCTACCACTCTCAGCTAATTTTTAAT
TTATTTTTTTAGAGATACGGTCCCACTCTGTTTCCCAGGCTGGTCTCAGAACTCCTGGGC
TCAGACAGTCCCCCGCCATGGCCTCCCACAGTGCTGGGATTACAGGCATCAGCCACCATG
CTCAGCTTGTTCTGCCATTTTCAAATGTGAATTTTATAGACACTTTAAACCACTTGAAAG
AGTGATGATGTTTTAATGATTTTCATTATTATTTGCAACTTCAAGCATTAAACACTGCCA
AATTAAGTTTCAAGTTTTCTCTTTACACAATATGGATGTACTTCATAATGGACTTCCTCA
TCATGATTAATGAGTGAAGTGACATTCAAACTTGGTAGCTTTTCAGTAGAACTTCCTTTC
CCAACATTTTTTCTGTTCCTTTAATTATGGCAATATCTGAGAGCTCTGAACATAAGTCAA
AGGTTTGATTATTTTTCATGTGGCTTCCTCTGCTTGGAACTTTCTGCCCCGCATCTTCCC
GTTGCCCCCTGTGTCCTCTTGTCATGCCCCTACCCTTTTTTGAGTGTGTCTATTTTCTGG
CACTACAAGACATAACAGGCTCATCTTGTGTTTTCCCTACCCTGACCCAGAATCAGCCAT
TACTTCAAGGAGCCCTGGTTCCATTATTGGAGAATACTATTAGAAACCAGGATCTGGTGC
TAGGCATGCTCATTTCTATTGGAGTGTCATACAAACAATTTGTAAATTGTTTGTAGGTCC
TCCCAGTGGATAGGATTAGGAAATAAAACATGCATACTAACCATGCATACACACACATCT
ACGTCTATTTCTGTATCTGTCTGTATACATATTAAAATAAACATGGGTTGATAACTAATG
TTTCTGCTGTAATCCACAGCCTTCATCCTAGCCTGCCACTCTTCTTCTTTTTAGCTTTTT
CAACAGTGGGAAATGTGGCTCTTGTTATGTACACTTTATTCACTTATTTGTTTGACCCTA
GTATCATAAAGTAGTTCCGTATGCCTGTAACAGATCGACTAACTAGAGTCCATTATTTGC
GGAAAGATCTTTTTGTCCGAAGCGTTACCGCAGGGGTGTCCAATCTTTTGGCTTCCCTGG
GCCACACTAGAAAAAGAAGAATTGTCTTGGGCCACACGTAAAATACACTAACACTAACGA
TAGCTGATAAGCTAAAAAAAAAAAAATCAAAAAAAATTTCATGATGTTTTAAGAAAGTTTAC
TAATTTGTGTTGGGCCACGTTTAAAGCCATCCCGGGTCGCAGGTTGGACAAGCTTGCCTT
ACAGTATCCAGTCAAAATAATGTTTTCCAAAATTACTTCTTTTCTTTTTCATCCCTTTCA
GTGTGGCCGTTATTTATAATGCAGTTTGGTTCATTAGTGTTTTTATTACAAATACACCCT
CAGCCTTCATATCCTAGTTTTAATGAATTATTACGGTGAAACATAATAAGAGTCAGAGCT
ATACAGAAAGGTCTACTCAGAGGTGCTTTGTTCCCTCCTATTCTGTTCCCACTACTCCTA
CTTTCCACTGACCCTGTAAGCATCATATTTATTTTTAATGGCAGTTACATTTTTACCAAG
TGCTTACTATCTGTAGGCACTTGGTGTGTATTGCTTCTTTTGGTGTTCACAGCAACCTCT
TGAGGTAAGCACTATTATTATCCCCCCTTTTTCTTTTTTCTTTCTTTCTTTCTTTTTTTT
TTTTTTTTTTTTGACAGTCTTACTCTGTTGCCCAGGCAGGAGTGCAGTGGCGCAATCTCG
GCTCACTGCAACCTCTGCCTCCCAGGTTCAAGTGATTCTCCTGCCTCAGCCTCCTGAGTA
GCTGCGAGTACAGGCACAAGCCACCACGCCCGGCTAATTTTTGTATTTTTAGTAGGGATG
AGGTTTTGCCATGTTGGCCAGGGTGGTCTCGAACTCCTGACCTCAGGTGATCTGCCCGCC
TCGACCTCCCAAAGTGCTGGGATTACAGGCACGAACCACTGCACCCGGCTATTATCCCCA
TTTTTTAGATGAGAAAGCTGAATCCCAGAGAGCATAAGAAGCTTGTCCAGAGTGACATCT
CTGATGCATAACCAGTACTCAAACCTATTTTTCTGACACCAAGGCCTGTGTGTAAACTGT
AAAGGGGCTGCTTGGCACCTACTTTCCTAAAGTTGTCCTATCCCTTCTCTGTCTGGGTCT
TCCTGAAGCTTGGCACTTCTGAAGTCACCTCTCTGAAAACATTCTGGTAACTGTTAAATC
CCTTGTTCTAGCTATTCATGTGTTCTGTGTGGTTAAACAAGGTTCACAATGGGCCACCTG
GCCTTTGGAACTTGGGTGAAGAGGCTGCCTTCAGTTGATCCTCCCCACTCCCATTTTCAA
AACATGGGTTTACATGAGTTATTTGTGAATTAGGAAACATAACCATGTTTTGAGCCTTCA
TAGAAAACAAACGTCTGGGGTCATACAGGTTAAAAGGAGTAACCAAATTCGGCACTATCA
TTGTTCTATTCAGTAGACAATTCTGGGGCCTTTCTGTGTCTCAGGTTCTGTACTAGTTGT
TTCAGGACTTTGGGATAAATACAAACTATCCCTGCCCTCAGGGGGCTTAAGGTCAGGTGT
ACAAGTGACTCTAATGTGAGGCAAGGCTGGATTCAGTGCTGCATATCTAATGCTATGGGA
ATTCAAAGAGGAAGTGATCAGAATGAGAAGGGAGGGATGGATCATTCCAGGAGAAGCTTC

AGGGAAAAGCAACATTTAAAATGAGACTTTTGAGAGTGAGGGAAATTTGGACAGGTGGAT
ATAGAGGATGCAAGGCTAGAGGAAAGGTTTTAGCCAGAAAGTCTGCTTGGGCAAATGCCT
GGGTAAAAAAAGAAAATCCACTTTGGGAGGACAAGGCGGGCAATCGCCTGAGGTCAGGAG
TTCGAGACCAGCCTGGCCAACATGGTGAAACCCCGTCTCTACTAAAAATACAAAAATTAG
CTGGGCGTGGTGGTGGGTGCCTGTAATCCCAGCTACTTGGAAGGCTGAGGCAGGAGAATC
ACTTGAACCCAGGAGGCAGAGGTTTCAGTGAGCCGAGATTGCGCCACTGCACTCCAGCCT
GGGCAACAAGAGTGAAACATCTAAAAAAAAAAAAAAAGAAAATCACAGGGCAGTGTGGGGA
ATGGTGAGTATTCTAATTTGGTTGTGGCAGAGAGGATGTAGAAGGAAGTGATAAGAGAGA
AAGCCGGATAGGAGGGCCTTTGTGCCAGTTAGGATGTTCTAGACTTCCAGCCAGGTTGCC
CAGCTCAAACTGGCTTAAACAATGAGGGGGTTTATTGGCTATGTAATTGGGAAGTGCAGA
GGTAGCTCAGGCCAGATCAGTTTGATCCACTGCTCCATTATGATGTCAAAGACCCATGCG
ATTTCCACCTCATTATTCTGCTGTCCATAGAGCCAACTTCATCCTAAGGCCAGTCCTTGT
GGTCAGACAAGGGCTGCCAATAGTAATCTGGGTGCAAGTTTCTTTGAGAAAATCTTTCTG
TGTCAACTCTCTTAAAAGGGGTGAAAAATCTCTCCTTAAGTCCCACTGGCCAGAATGGGC
CCATGCACCCATTTCTTAACCAGTCACTGGCAACTGGGGGTGGGATTGCCGTTTGCCCAA
TCAGGTCCATTTCTGGAGCTAAGATTAAACTCCATTTCCCTTGGGACACATTGAACAGAA
TCAGAATTCGATGAAGAAGGAAGAAGCGGAGAATTGGTTTGGTGTTGGGTAGGCAACCAA
AAATAACCTCTGTTGCCTCAAGTGCCAAGAAAGTGGTGTTTTGTGCTTGTTAGGGTAAAA
ATGGGGATCATGGAAAATATTTTAAGTTTCATAGACCAAAAAAATATTCCAGTGTTTCATC
AAATCTAAGAGGCTATCAATTATAAGATATACCATTATTTTATGTACCACCAAGGAAGAA
AAAATGCTGCCAGTGAAGTTAGGATGTATTGCAGGTTGGGTTCTCTGGGAAGCAGGCTGA
AAAGGAGGTGAGAATGCAGGACATTTATGGGAGAACACCCTTGGGATTAATACTGGAGGA
GGAGAACCAAGCAGGGTTGGTGGGGCACAGGGAGAAGTTGGGATGCCATGCAGTCACAAC
AAAGGCCTCAGCCAACCCCACGGGGAGCTCGAGAAGCTGAGATGGCCCTTCAGTGTTGCC
CTGCCTTGTGGTGAGTGAATTGGGTCTTCATATCCCCATGTTGACTGGTCATTGGATGTG
GGCTCCCTTAGGAATGGGCATCTCTTCAGCAGAGGTAGCTTTCTTCAAAAGAGGTGATTC
CAAAGAGTCACCCACTCACTGAGGGCTGTCTGCTGGCAGCATTCTCAGCCACTACTCAAA
GATGACCTGTCCAGGAAGGGGAACCTAGGTGGCATGACACATTGTCTATTACAACATGCT
ACTGATTATAAGAGCCGGGAGGTGGGGGGCAACACAATGTCTGAGATATTAAAATGGAAG
TCTCTTAGAAGAAATGGATAATTCTATAATTATAGTTAATCAGAAAGGGGAAGAAGTGGG
GAAATGGACCAAGGGCCTGAGAGAGAAAACAGACGCAACAGGCCACTAGAAAGATAGGAC
ACTGGAGGGTGGGAAGCCCTAGCAGTTTCTTCCAGGGTGGGCTGGGCACGGTGGCTCATT
CCTGTAATCCCAGAACTTTGGGAGGCCGAGGCGGGCAGATCATTTGAAGTCAGGAGTTGG
AGACCAGCCTGGCCAACTCCTGTTTCACCCTGTCTCTGCCAAAAATATAAAAAATTAGCC
GGGTGTGGTTGCATGCGCCTGTAATCCCAGCTACTTGGGAAGCTGAGGCAGGAGAATCGC
TTGAACCCAGGAGGCAGAGGTTGCAGCGAGGAAAAATCGTGCCACTGCACTTGAGCCTGG
GTGACAGAGTGAGACTGTCTCAAAAAAAAAAAAAAGTTTCTTCCAGGGTGGCTTCTGTGCC
AGAGTCAGGTGCCCCAGCTACCTCTAATTTATGGTCCTCCTGCACTGGGAAACAGATTTT
CTACTTTTGGTTTCATGATAAATAACATTTCCCCCTGATTTTAAAAGTTATGGATTTGGC
TGGGCATGGTGGCTCATGCCTGTAATCCTAGCACTTTGGGAGGTCAAGGCAGGCAGATCA
CTTAAGGTCAGGAGTTCCAGACCAGACTGGGCAACATGGTGAAAACCCGTGTCTACCAAA
AAAAAAAAAAAAAAAAAAATTAGCCAAGTGTGGTGGTACATGCCAGTAGCCCTAGCTACT
CAGGAGACTGAGGTGGGAGGATTACCTGAGCCCAGGAGATCAGGCCTGCAGTGAGCTGTG
ATTGTGCCATTTTACTCCAGCCTGGGTGACAGAGTAAGACCCTGTCTCAAAAATAATAGT
AATAGGCTGGGCGCGGTGGCTCAAGCCTGTAATCCCAACACTTTGGGAGGCCAAGGCGGG
CGATCAATTGAGGTCAGGAACTCAAGAACAGCCTTGCCAAAATGGTGAAACTCCGTCTCT
ACTAAAAATACAAAAATGAGCCGGGTGTGGTGGCGCATGCCTGCATTCCCAGCTACTCAG
GAGGCTGAGGCAGGAGAATCGCTTGAACTCGGGAGGCAGAGGTTGCAGTGAGCCGAGATT
GCACCACTGCACTCCAGCCTGGGTGACAGAGTGAGACTCCATCTTAATAATAATAAAATA
ATAAAAATTTTAAAAAGTTATGGATCTGGATGGAGGGAAATGGAATGTATAAAAGAAGTA
AACATACACAAGAAGATACAAATACAGAATAAAAGTAAAATGCAACCATCATCCCACTAC
CCCGATACCAGGGTATCCGTTTTTACATCTTTTCTTTCATTCTTTCTGTCTTTATATAAT
TGTATAAATGCTGCATAAACCTCCTCTTGCCTGCTGCCTCCTCAAAGACCTCCCTCCCTC
CTTCACTGCCCTTCTGCTCCTGGAGAGCCACCCTCTCTCCATTTATCCTTCCTATCAGCT
TCAGGTTCTTACCATGTTAACAAAAAGAAAATCTTATAAGCCTGTCACTCTCTACATACG
CCGCACCTCCTTTCATTCATAGCCTTTAAAACATATATATAGCAGTTATTGTGGTTATTT
TTCTGTTCACAAAATAAAAAAACACTCTTTCTAGAAAACTGGAATATAGAGGCAAGCTTT
TTTTTTTTTTCAGACGGAGTTTCGTTCTGTCGCCCAGGCTGGAGTGCAGTAACGAAATTA
CAGCTTACTGTAACCTCTGCCTCCTGGGTTCAAGATATTCTCTTGCCTCAGCCTCCTGAG
TAGCTGGGATTATAGGTGCCTGCCACCACACCCGGCTAATTTTTGTATTTTTAGTGGAAA
TGGGGTTTCGCCATGTTGGTCAGGCTGGTCTCGAACTCCTGACCTTGTGATCTGCCCATC
TCGGCCTTCCAAAGTGCTGGGATTACAGGTGTGAGCCACTGCACCCTGCCGAGGCAAGAT
TTTTTTTTTTTTTTTAAGAAAACCCAGTTATTCCATTACCCAATGAAACTCTAAACATG
TTGATGTACATCCTTCCAAAATTTCTTTTTATGACAACATGCTTTTTATTTTTAATTATT
TTTATTTTATTTTAAGGTCCGGGGTACATGTGAAGGATGTGCAGGTTTGTTACATAGGTA

AACGTGTGCCTTGGTGGTTTGCTGCACCTGTCAACCCATCACCTACGTATTAAGCCCCAC
ATGCATTAGCTATTGATCCTGATGCTCTCTCTCCCTGCTGGCTCCCCAGCAGGCCCCGGT
GTGTGTTGTTCCCCTCCCTGTTTATGAGAACACTTTCTTGACATAAAGATTTCATTTATT
CCCATGGAATTCTAAAGGCTTTTCATACTTGTGAAGGAATAATAGTTTAGAAATAAACTG
AACTTTAAAAGATACCATTTTGAAAAATAATATACAGCCATCAAAAATTATATTTATGGG
AACTATGCAATAATATTAAACTCTATCATCTGTTGACTGCCTCCTATATTCCAGAAACTT
TACATACACCAATTCTAATCCTTACAAGAACGCTGTGTAGGCTTTAGCATTAGATGGACC
AGGTTTCACCAACTGTATGGTCTTGGATAAGTACCCAACCTCCTGTCCCTAAGTTTCCTC
ACCTGTGAAAACACGGTTTCTACCAGCTTTCAAATAAGATGATCAATATAAGGCACTTGG
AACAGAACCTGACACATCATAAGCACTCTATAAATGTCTATTATCACCAAATAATTCCAG
GTGCCTTGAAAATTTAAATGAAAAACAAAATCAAACCATGACAATACTAGAAGCAAATTT
AGGTGAACACTTTTCTAATCCGGGGGTGGGCGGGGGCTGGGGGGAGGCAGGGAGAAGACC
TTTTTTTTTTCTTTTTGAGATGGAGTCTTGCTCTGTCCCCAAGCTGGAGTGCAGAGGCGT
GATCTCAGCTCACTGCAACCTCTGCCTCCTGGATTCAAGTGATTCTCCTGCCTCAGCCTC
CCGAGTAGCTGGGACTATACAGGTGCACACCACCACGGCCAGCTAATTTTTGTATTTTTA
GTAGAGATGGGGTTTACACCCTGTTAGCCAGGATGGTCTCAATTTCTTGACCTCGTGATC
CCATCCGCGTTGGCCTTCCAAAGTGCTGGGATTACCAGCATGAGCCACCGTGCCCGGCTG
GGAGAAGACCTTTCTAAGCATGATACCAAAGGCAGAGACAATAAAGGCAAAGAATTGACA
GAATTCACTATCCGATAAAAATCACTTCTGTGGCCGGGCGCGGTGGCTCACACCTGTAAT
CCCAGCACTGGGAAGCCGAGGTGGGCGGATTGCTTGAGGCCAGGAGTTCAAGACCAGCCT
GGCCAACATGGCAAACCTCCTGTCTCTACTAAAAATACAAAAAATTAGCTAGGCATGGTG
GCATGCCTGTAGTCCCAGCTACTCAGGAAGCTGAGGCATGAGAATCACTTGAACCTGGGA
GGTAGAGGTTGCAGTGAGCCAAGATCATGCCACTGCACTCCAACCTGGGTGACAAAGTGA
GACTCTGTCTCAAAAAAAATAACAATTAAAATAAAATCACTTCTGAATGGTGGAAAGCAC
CACAAAGTTAGAGGTCAAGCAATAATTTGGAGAAAAGAATTAGTAATTTGTTGGACAGAC
AAAAGACTTTTTTAATATAACAAAAACTTTAAAAATTAAAAAAATACACATTCGAGGACA
TTTTCCTAAAAACACAGGCAAAGGACATAAACAGCAAAGCAAGAAGACAGCTTGATGTGG
CCATTTTATCCAGGGGGACATTTTGGTGAGCCCTATGGACACAGCTGCCATGATGCCAAC
AATGTGACAGCTGTCCCCTTCAAAATGCGTTAGCCCCAGCTCTTCCTCTCCCCCAACCTC
CAGTCCAAAGGACTTGCACTTTCTACTTTACTCCTTTCTGCATTGTTTAATTTTCTTTTA
CAAATATGTTACTTGTCATCAGAAAAAATAAAGAAATAAATAAACTGTTAGAGTGTTAGC
CCCTTAAAGGGGAGCAAGAATCACCTTTCTAAAAGAAAGTTTATGTTAAATATAATATTA
GCATATGTGAATCCTGAGAGAAAAGTTAACAGTTTAGTTGAGTTATTTCCTCTGTAGTCT
GGAGCTAAAAATAGGGAATCTTATTCTGTCCTAAATCTTTTCCTTCCTCCACCCAGTGTC
TGTCTGGATCGAATTCATTCATTCACTCAGTAGGCACTCACTCAGCCAGGCATGGTGCTA
GGCCTCAGGACCTCGCTGTGAACCAGAAACTGTCCCTACCCCCATGGTGCAGGCATTCTG
CTTGGGAGTTGGAGGAGGAACAGGTAAAAAATAATTAAATATTCAGGTTAACGATATATT
GTCAGGTTTGAGGATTGAGGAAAGGGCGCAGAGAGTGGCAAGGGCTGCTGTTTTAGATAC
AGTGGCCAGGAGGCCTCCGATGAGGTGACCTTTGAGGAGAGACATGCAGGAGATGAGGGG
ACAGTGAAGAGGATTTCTAAGAACACTCCAGGCAGACAGAACAGCGACAGCCAAGGCCCT
GAAGTGGGTAGGGGCCTGGTGTGTGTGAGGAACCTCAGGATTGCCATCATGGCTGGAGCA
GAGACATGAAGCAAGAAGGCCATGGAGATGAGGGCAGGGAGATCCCGGAGTGGGGAGATC
AGATGGGGCTCTGTGTATCATGCAAAGGACTTTGCATTCTGTTCCAAGAGCTGGGAAGGT
TGACATAATTAGGAAAAAAGCCCAGAAAAGCAGAGGTATCCATTTTTCATGGTAAAGATG
ATAATTTCAATTAAAACACGATTCCTGGATATATGTAATTTGTAGGCCAAATGGTGCCCA
ATCCCTACCTCCCTCACCCCCTCACTTCCCTATCCCTAAAACCTGTACCTCAACTCCCGT
TCGTAAGTGATGGGAGTTAGGAATAGAGAAATCTCCCGGTTGGGTTTTCTGAGCAAAGAG
GTAACATAGCAGCTCTGTTATTTCTTTCACGTCTCCAAGGGAACCATGACTCACCCTTAG
CTATCCCCCGGGAATGTGGCCCTCAGAGTGTTCTTTTACTGATTCGTGATTTTGTTATGT
ACACCTGGAGTGATGGAACATACCATACCAGCTTGTCAGGGTTGCTTTGTGCAAAGATCG
ATGACGTGTGTGAACCCGGATCCATGCTTGGGGTCCTGAGTTTCAGGTGCCATGGCCAGT
TGCTAGCAGGTTGTATGTGTGTGACCAGCCCCTATGTGAGTCTCTCAGACCCTGAAACTC
CAAACAGGCTTCCCTGGGCAGAGACATTCTGTCCATGCTCTGTGGCTTGCTGCTCGAGAG
GGATAGATCACATCCTGTGTGGCTTCTTCTTAAATGAAGAAGGACATTGGAAGCCTGTGC
TGGGCTTCTCTGGACCCCCCGATGTATATGTATGTATATTAAAGAGAGACCAGGGTCTCA
CTCTGTTGGCCAGGCTGGTCTTGAACTGCTAGCCTCAAGAAATCCTCCCGCTTTGGCCTC
CCAAAGTGCTGGGATTACAGGCATGAGTCACCATGCCTGATGTATATATTTTTCCAGCTC
CCTTCTTTTCTGTATCATTTGCTATTACTACCTCTTAGCTATTAGTATAAACTGATCTTG
AGTTGTGTAAATCTTTCTGGTGATTCACTGTGATGGGATGATTGTGTCCTCTCAAAATTC
CTATGTTGGAGTCCTGACCCATGGTACCTCAGAAAGTGACTGTATTTGAAGATAGGTCTT
TAAAGAGGTCATTGTAAATTAATTAATAAGGTCATTAGGGTGGACTCTAATCCGATATGA
CTGGTATCCTTATAAGAAAAGGAAATTAGCACACAGACACACAATCAGAGGGAGAAGACA
GCCAGTCATCTACAAGCCGAGGAGACAGACCTCAGAAGAAACCAACCCTGCCTGCACCTT
GATCTTGGACTTCTAGTCGCCAGAACTGTGAGAAAACAAATCTCATGTTTAAGCCAGAAC
CTAGCACGTGGTACTTGTTAAGGCATCCCTAGAAAACTAATACACTCACTGAATGAGGCA

GGTAGCTGTTTCTTTTATTTTTTGAGACAGAGTCTCACTTTGTCTCCAAGGCCAGAGTGC
AGTGGAGCGATCACAGCTCACTGCAGCCCCTGCCTTCCAGGCTCAAGCCATCCTCCCACC
TCAGCTTCTCAAGTAGCTGGGACTACAGGCATGCACCACCACGCCCAGCTAATTTTTGTA
TTTTTTTTTTTTTTTGTAGAGACGGGGTTCACCGTGTTGCCTAGGCTGGTCTCAAACCC
CTGAGCTCAAGCAATCTGCCCTCCTTGGCCTCCCAAAGTGTTGGATTTACAGGCGTGAGC
CACTGTGCCTGGATATGGTAACTTTTTCATATGCTATTTGCTTGATGATTATTTTTCTGT
TTCTGATATAATGCTTTTTATTAGAGAGTTATCTGTTTGTTTTTATTTTTTAATGTTTGA
ATTTAAAAAATTAGTATAATTTGCATAATTGAAAAATTATATTTGAATAATTGAAATATA
TTTGTATAACCTTAAATTTAAAAACTATGATAGCGTATACAGTGAAATTTTCCTCTCATC
CCTTTTTTCCATTTAACCAGTGCACTTCCCAACAGCCAACAGATAATTTTAGTTTCCTCA
CTCCCTGAGCTATTTTATGTATATGCAAGTAGATATGTACATACATATTTCTGCCTTGTA
ACACAAATAGTAGCATACTATACAACTGCTCTGCTTCTTCCTTTTTTTAGCTAAGAATAT
TAAAAGAGTGAAAAAGATGTACGCTAACAAAAATCAAAAGAAAACTAGAGTGACATTATA
AGAACTGATGATGTAGATTTCAGAGCAATGATTACTGCTAGGAAAAAAGGGTCATTTTAC
ATTGATCAAAGAGGTCAACTCATCAGGAAGACATAATAATCCTAAACACTTATGTACTTA
ACAGAGCATCAAAATACATGAAGCATAAATGAAAGAACCGTGGGAGAAAGTAGACAAATT
AATGACTGTAGTTGAAGATTTCAGTATCCCTCTATGAAAATCAGGGTAGTACAAGTACAC
AGAAAATTGGTAAAGATATATGACTTGAACAACATTATCAACCAAATTGACCTCATTTAC
ATTTGTGGAATGTTCCAACTAAGAACGTCAGAAAACATACTCTTTTCAAGTGCACATGGA
ACATTTACCAAGATAGACAATATTTTGGGTCACCGCAAGTCTCAACACATTGAAAGGATT
CAGATCATATAAAGTATGCTCCATGACCATGATGGAATTGAATTAGAAACCAATAATGTA
TCTCTGGAAAATACACAAATATTTGGAAATTAATATGCCTTCTAAAAAATTTATGCATCA
AGAAGAAATCAAAAGGGATATTTGAAAGTACTATGAACTGATGGCCAGGCATGGTGGCTC
ACGCCTGTAATCCCAGCACTTTGGGAGGCCGAGAAAGATGGATGAAGTCAGGAGTTCAAG
ACCAGCCTGGGCAACATGGCAGAACCCCGTCTCTACTAAAAATACAAAAAATTAGCCGGG
CGTGGTGGTGGGCGCCTGTAATCCCAGCTACTCAGGAGGCTGAGGCATGAGAATCGCTTG
AAGCTGGGAGGTGGAGGTTGCAGTGAGCCAAGATCACGCCACTGCACTCCAGCCTGGGCA
ACAAGAGTGAAACTCGGACAAAGAAAGAAAGAAAAGAAAGAGAGAGAGAAGGAAGGAAGG
AAGGAAGGAAGGAAGGAAGGAGGGAGGGAGGGAAGGAGGGAAGGAAGGAAGGAAGGAAAG
AAGGAAGGAGGGAGGGAGGGAAACAAAAAAGAAAAAGAAAGAGAAAAAGGGAGGAAAGAA
AGAAAAGAAAGAAAGAAAAAGAAAGAAAGAAAAGAAAGAAACTAACTCACCATTTAGTAA
CCATAGTAGTAATAACTGATTTGGGCAAGAGTCATCAATGGGTGAAAGCTTGATGAGGAA
TAGGATACTTATATACCCCCAAACAAAGCCACAAATTATTAATAACAAAGGGGAAAGTAG
TAATTTTACAATGGCAAGACACCACTGTAAACGGATAATGAAAGAACATCAACAATAATG
GGAAAAAAATGATGCAATTTACCTCCTGCTATGCTGCTCTGAGAACAAGCATAATTTCTG
TGGTAGTCCTCCAAAAATGCATAACCTGAGTCTGTTCGTGGGGAGGCATCAGATAAACCC
AAATTGAGGGACATTGTACAAAACAACTGGCCTATATGCATCAAAAATGCCAATGTCACA
AAGTCTGGGGAACTCTCCCCAGTTCAGGAGACAATTAAATGTAATGTGTGATCCTGGACT
GTGAAAAAAGTAACTATAAAGGATGGTATTGAAACAGTTGTCAAGATTGGAGTATAGTAG
TAACAAATCAAGGCTAAATTTTCTTATTGTACTGTCGTGTGTAAAAGAATGTCTTTGTTA
GAAAATACACACTGAAATATCCACAGGTGAAGGAGAACAATAGCTCCAATTTACAAATGC
TTCTGAAAAAATATATATATACACACACACATACAGAGAGAAGGCAAGTGATAAAGCAAG
TGGGGCAAGTTTTTTTAAACTGGTGAATCTGGGTAAAGGATATTCAGGAATTCTTTGTAC
TATTGTTGCAACATTTCTGTAAGTTTGAAATTTAAAGTTTAAAAAGAAAGAGAGGCCGGA
CTCAGTGGCTCACGCCTGTAATGCCAGCACTTTGGGAGGCCGAGGCGGGCGGATCACGAG
GTCAGGAGATCGAGACCATCCTGGCTAACACGGTGAAACCCCGTCTGTACTAAAAATACA
AAAAATTAGCCGGGCGTGGTGGCGGGCGCCTGTAGTCTCAGCTACTCGGGAGGCTGAGGC
AGGAGAATGGTGTGAACCCGGGAGGCGGAGCTTGCGGTGAGCCGAGATCGTGTCACTGCA
CTCCAGCCTGGGCGACAGAGCGAGACTCCATCTCAAAAAAAAAAAAAAAAAGGAGAGAGAA
GGCATAAACAATATTAAAAATAAAACAGAACATTACTATAGACGTAGTAGAGATTTTAAC
GATCATGAAGAAAATACTAGAAATAAGTCTTTATGTTTTATTTTATTTTATTTTATTTAT
TATTATTATTTTTTGAGATGGAGTCTCATTCTGTAGCCCAGGATGGAGTGCAACGGCGCG
ATCTCTGCTCACTACAACCTCCGCCTCCCAGGCTCAAGCTATTCTCCTGCCTCAGCCTCC
CAAGTAGCTGGGATTACAGAAAAGCGCCACCACGCCCAACTAATTTTTGTATTTTTAGTA
GAGATGGGGTTTCACCATGTTGGCCAGGCTTATCTTGAACTCCTGACTTCAGGTGATCCA
CCTGCCTTGGCCTCCCAAAGTGCTGGGATTACAGGCGTAAGACACCGCGCCAAGCCCTTT
ATCTTTTAGATGTATATACTGAGATACTTATCATAAAATGGTGGGATATCTGGGATTTCC
TTGAAGATAATCCAGGGCGCCAGGTATGGTGGCTCACACCTGTAATCCCAGCACTTTGGG
AGGCCAAGGCCGGAGGATCACTTCAGCTCAGGAGTTCAAGATCAGCCTGGGCAACATGGG
GAAACCCTGTCTCTATTAAAAATACAAAAAGTAGCTAGGTGCGGCGGTAGCACATGCCTG
TAGTTCCAGCTACTTGGGAGGCTGAGTTGGGAGGATCACTTAAGCCTAGGAGGTAGAGAT
CAGCTTCAGCAACATGGCAAAACCCCATCTCTACAAAATATACAAAAATTAGCTGGACTT
GGTGGTGCACACCTATAGTCCTAGCTACTTGGGAGGCTGAGGTGGGAGGATTACTTGAGC
CCAGGAGGTAGAGGTTTCAGTGAACCGAGATCGTGTGACTGCACTCCATCCTGGATGACA
GAGTGAGACCCTGTCTCAAAAAAAAAAAACAAAACTGCACTCTTTTATGTATTTTTATACT

TTTGAAATGTTCTATAATATTTTTGAAATGTTCTATAATAAGAGAATATTGTGAAGACTT
TATAGAAATACATTTGAGAACCTAAATAGAGTAAATTACTAGAATCAAACTAAGGAAAAA
ATGGAAAATCTGAAAAAAATGCAATAATCATTAAAGAGATTGAATTGTCATTTGAAAGTC
TGTCCTCACAAAAGACCTCCTTCCTCTACTGTGGTCAGCATCTTTGGCATACATGAGATA
AAATGTATGGTCCAGTCCAGCCTCTAAGCCTGTGCAATTCATTCAGTCATTCAGCAAATA
TCTATTGAATACTACGTATGGGCCAGGCACTCTTCTGGCTACCGGGGATACATAAGTGAA
CAACAACAACAACAACAACAACAAAGGAAAAATGTCTCTAATTTTGTGGCACTTAATATA
CAACAAATAATAATGAATAAGTGATATATAGTATGTTAGGAGGAGAACATTACTATGGAA
AAATAGAGGAGGATAAAGGAGACTGAGAGGCAGAAGGATGGGCCAGTTTGTAATTTGAAA
GGCAAGGTTGTAGGCAAGGTTCAGATTGGAACACTGAAACACGGATTTCCCACAGAAATG
AACAGGTCTCCTTGGAGAGATGGCTAATTCAAGGTCTGGGTCAAGAAGTGTACAGGAAGA
GCCTGGAACATTTTGTTGCACCAAAAAGATAAAACAGAACAAAACAAAACTCGGTTAATT
AGTTACTGTTGGAAGATGCTAGCAAGCCAGTTCTTTAGTGATTTTTAAAAGACAGATAAT
TATAAGGAAACTTTTTAATAAACAAGCATTTATACTGTTTTCGACACAGACTACCACTGA
CTACTCAAATAATACATGAGGGGATATTTCTTTTTATGGAAGTATTCTGGCTAATAAGAA
GAATAGAATTAATCACCAGTGGCTGCCAGCACCACAAAAAGAGATACAACTAGATCTTAT
GTGTTTTACAATTTTTTTATATTTGGGGGGCTGGTCTCTTATATGTCTCTTTTGGAGAAA
CAGCCCACCACCTGGATTCATCTCCTAGGGCTTCCATAACAAAGTACCATAAAATGAGTG
TCTTAAAACAACACATGGCCGGGCACAGTGGCTCATGCCTATAATCCCAGCACTTTGGGA
GGCCGAGGCGGGCAGATCACTTGAGGTTAGGAGTTTGAGACCAGCCTGGCCAACAAGGTG
AAACCCCGTGTCTACTAAAAATACAAAAATTAGCCAGACATGGTGGCGGGCACCTGTAAT
CCCAGCTACTCAGGAGGCTGAGGCAGGAGAATCGCTTGAACCTGGGAGGCTGAGGTTGCA
GTGAGGTGAGATCTCGCCATTGCACTCCAGCCTTGGCAACAGAGCGAGACTCCATCTCAA
AAACAAACAAACAACAACAACAACAAAACACTCACGTTTATTCTCTCAAAGTTCTGGAGG
CTAAAACTCTGAAATCAAGGTGTCAATAGAGCTGTGCTCCCTCCAAAGGCTTTAGGGGAG
GATCCTTCCTTGCTTCCTCCTAGATTCTGGTGGTTTCTGGCAAGCTTTGATATTCTTTGG
CTTATGACTGCATAATTACAACCTCTACCTCCATATTCATGTGGCCATCTTCCCTCTATG
GGTGTCTGTGTCCAAATTTCCCTCTTATGGACACCAGTCATATTGGATTAGGCTTCCCCT
AATCCAGTATGACCTCATCTTAACTTGATTACATCTACAAAGGCCCTATTTCCAAATAAG
GTCACATTCACAGATTCCAGGTAGACATGAAATTTGGGTAACACTATTTAACCCAGTATA
CCACCTATGAAGTATTCTTTTTATTTTTCTCCTCAGCTTTTATTTTAAGTTCTGGGGTAC
ATGTTTAGGACGTGCAGTTTGTTACATAAGTAAACGTGCCATGGTGGTTTGCTGCACAGA
TCAACCCATCACCTAGGTATTAAGCCCAGCATCCATTAGCTATTTTTTTCTGATGCTCTC
CCACCACCCGCCCCGCCCCCGACAGGCCCCAGTGTGTGTTGTTCCCCTCCCTGTGTCCAT
GTGTTCTCATTGTTCAGCTCCCACCTATAAGTGAGAACATGCAGTGTTTGGTTTTCTGTT
CCTGCATTTGTTTGCTGAGGATAACAGCTTCCAGCTCCATCCATGTCCCTGCAAAGGACA
TGATCTTCTTCTTCTTTTTTATGGCTGCATAGTATTCTGAAGTATTCTTGACAAAAAGAA
AAAATCAACCTGAATCTATTCAAGCCTCTAGAGTCAACTAAAAATTTACAGAAAATACAG
GAGACTGAGTAACGTGTTAAATGACATCACAGGGATGCAATTAGCCAACTCCAGACTATG
GGAAACTCTACAGGACAAACTACCTGGTATCTTCAACAAATAAATTGCAGGGAATAAAGA
CATGATGGAGGAACCTAGACTAAAAGAGACTTAAAGATGAACCAGTAGCTATGTTTGGAC
CTCATTTGGATCCCAATTCAAACAAACTTTAAAATACACACCCATTTATGACACTTATGA
GACAATTGGAGATTTGAACACTGACTTGAATATCCATTGATATTAAGGAATTATTTTTGA
AAATGCGGAATAATACTAAGGCTATGGTTTTTTTTAGAGAGTCCTTATCTTTTAGAAACA
TATACTAAATTTGCAGATAAAAATGACTTGATGTTGGGGATTTGTTTCAAAATAATATGG
GAGTTGGGGGATGTGGGTGCAGATATGGCTGTGACAAGATGGTTCATGGGCTGATAATTG
TTGAAGCTGGGTGATGGGTACATGTGGGAGTTCATGATACTATATTTTTATGTGTTTCAA
TCTTTCCATAATAAAATTTTTTTTTAG**AAAAAAGAAAGAAAGGAAAGAAGGAAAGAAAG**A
AAAGAAAGAAAGAAAGAAAAAGAAAAGAAAGAAAGAAAGAAAGAAAAAGAAAAAGAAAGA
AAGAAAGAAAGAAAGAAAGAAAGAAAGAAAGAAAGAAAGAAAGAAAAAAATACGGTATCAG
GATAAGCCTAAATAAGAAGGTGACATTTGCTGTGGATCGTTCAGCATGTACAGGGCCCCT
GTCACCATCTGGCTTTAAAAATACTAAAAAATAAATCTTGTGTAGGGGCTGTGAGAGATT
AAAGACGGCCACAAATTCTTTTTTTAACCCAATTCTCCCATACCATTTATTTTAAAATTT
ATGCATTTTTATTAATTAATTTATTTATGTATTTAGTTTTAGAGACAGGGTCTCGCTATG
TTGCCCAGACTGGCCTTGAACTACTGGGCTCAAGTGATCCTGCCTCAGCCTTCTGAGTAG
CTGTAACTACAGGTGCCCACCACTGTGACTAGCCTCTGCCACAAATGCTTTTCTAATTCT
CCCCTTACCTAAAGCTAATTCCCCTCCCTTTGAATTTGGCCTGGCCTTAGAGACTGGCTT
GACCAATAGATGGCAGCAGAAGTGAAGTTCCAGGGCTTCTGAGGCTAAGTCATAAGAAG

Long fragment:

Primers pair chosen based on literature:

DNA Damage Induced by T-2 Mycotoxin in Human Skin Fibroblast Cell Line—Hs68

Janik-Karpinska E, Ceremuga M, Niemcewicz M, Synowiec E, Sliwinski T, Stela M, Bijak M. DNA Damage Induced by T-2 Mycotoxin in Human Skin Fibroblast Cell Line-Hs68. Int J Mol Sci. 2023 Sep 22;24(19):14458.

Primer-BLAST - Detailed primer reports
https://www.ncbi.nlm.nih.gov/tools/primer-blast/

Primer pair 1

	Sequence (5'->3')	Template strand	Length	Start	Stop	Tm	GC%	Self complementarity	Self 3' complementarity
Forward primer	AGGGCAAAGGATGTGTTACG	Plus	20	14680	14699	58.18	50.00	3.00	2.00
Reverse primer	AGTGGTTTCTGGTGCGACTT	Minus	20	15697	15678	59.82	50.00	3.00	0.00
Product length	1018								

Products on potentially unintended templates

>NC_000023.11 Homo sapiens chromosome X, GRCh38.p14 Primary Assembly

```
product length = 1018
Features associated with this product:
   hypoxanthine-guanine phosphoribosyltransferase

Forward primer  1          AGGGCAAAGGATGTGTTACG  20
Template        134474844  ....................  134474863

Reverse primer  1          AGTGGTTTCTGGTGCGACTT  20
Template        134475861  ....................  134475842
```

PCR Optimization

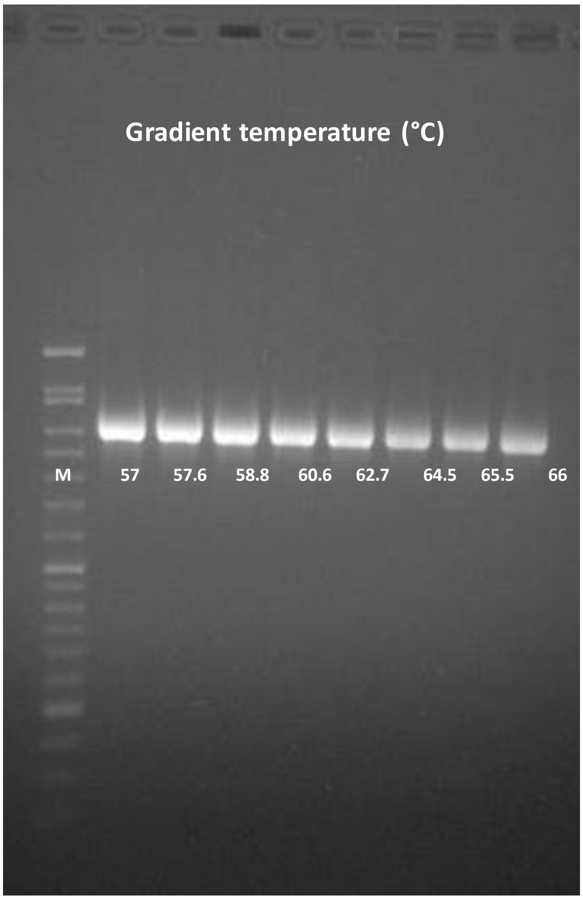

Gradient optimization of a PCR assay for *HPRT1* gene (long fragment). Gel photo showing PCR products formed at different annealing temperatures in a gradient optimization.

Real-time PCR Optimization

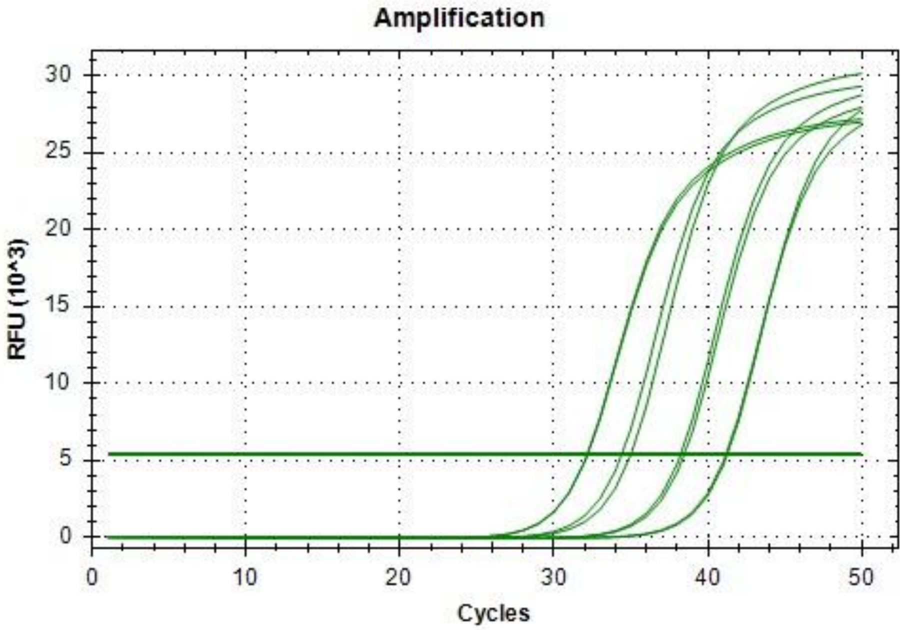

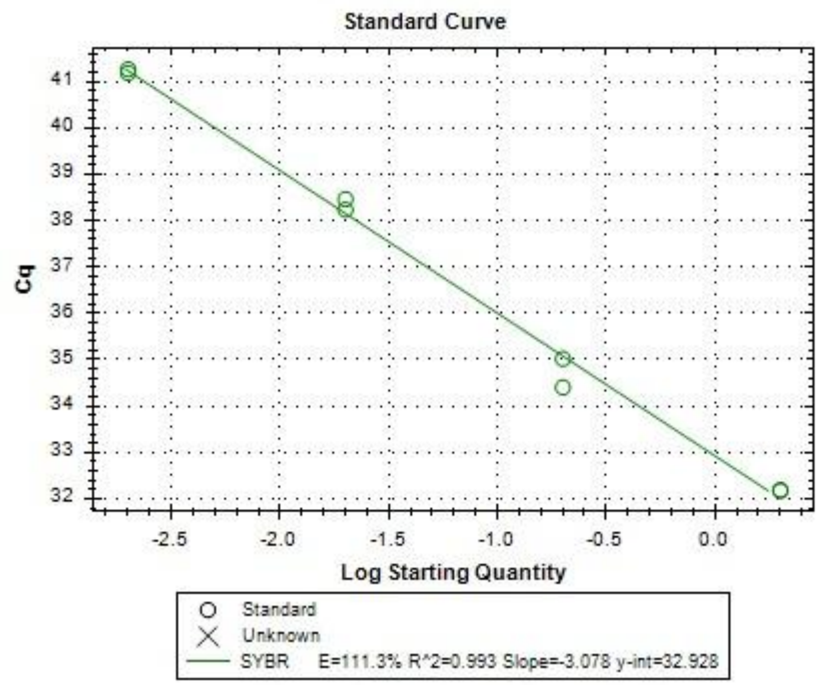

Standard curve with diluted sample: 1/1; 1/10; 1/100; 1/1000.

Short fragment

PRIMER PICKING RESULTS FOR

Template masking not selected
No mispriming library specified
Using 1-based sequence positions

OLIGO	start	len	tm	gc%	any th	3' th	hairpin	seq
LEFT PRIMER	5640	20	59.09	55.00	23.89	0.00	0.00	TGGGAAAGGCAGATCTGGAG
RIGHT PRIMER	5831	20	59.00	55.00	0.00	0.00	0.00	GGGGTGTGGGAGGACATAAA

SEQUENCE SIZE: 40504
INCLUDED REGION SIZE: 40504

PRODUCT SIZE: 192, PAIR ANY_TH COMPL: 0.00, PAIR 3'_TH COMPL: 0.00

```
  1 AGCTTCAGGCGGCTGCGACGAGCCCTCAGGCGAACCTCTCGGCTTTCCCGCGCGGCGCCG


 61 CCTCTTGCTGCGCCTCCGCCTCCTCCTCTGCTCCGCCACCGGCTTCCTCCTCCTGAGCAG


121 TCAGCCCGCGCGCCGGCCGGCTCCGTTATGGCGACCCGCAGCCCTGGCGTCGTGGTGAGC


181 AGCTCGGCCTGCCGGCCCTGGCCGGTTCAGGCCCACGCGGCAGGTGGCGGCCGGGCCCTG


241 AGGCGCGGGATCCGCAGTGCGGGCTCGGGCGGCCGGGCCCAGGGAACCCCGCAGGCGGGG


301 GCGGCCAGTTTCCCGGGTTCGGCTTTACGTCACGCGAGGGCGGCAGGGAGGACGGAATGG
```

361 CGGGGTTTGGGGTGGGTCCCTCCTCGGGGGAGCCCTGGGAAAAGAGGACTGCGTGTGGGA

421 AGAGAAGGTGGAAATGGCGTTTTGGTTGACATGTGCCGCCTGCGAGCGTGCTGCGGGGAG

481 GGGCCGAGGGCAGATTCGGGAATGATGGCGCGGGGTGGGGGCGTGGGGGCTTTCTCGGGA

541 GAGGCCCTTCCCTGGAAGTTTGGGGTGCGATGGTGAGGTTCTCGGGGCACCTCTGGAGGG

601 GCCTCGGCACGGAAAGCGACCACCTGGGAGGGCGTGTGGGGACCAGGTTTTGCCTTTAGT

661 TTTGCACACACTGTAGTTCATCTTTATGGAGATGCTCATGGCCTCATTGAAGCCCCACTA

721 CAGCTCTGGTAGCGGTAACCATGCGTATTTGACACACGAAGGAACTAGGGAAAAGGCATT

781 AGGTCATTTCAAGCCGAAATTCACATGTGCTAGAATCCAGATTCCATGCTGACCGATGCC

841 CCAGGATATAGAAAATGAGAATCTGGTCCTTACCTTCAAGAACATTCTTAACCGTAATCA

901 GCCTCTGGTATCTTAGCTCCACCCTCACTGGTTTTTTCTTGTTTGTTGAACCGGCCAAGC

961 TGCTGGCCTCCCTCCTCAACCGTTCTGATCATGCTTGCTAAAATAGTCAAAACCCCGGCC

1021 AGTTAAATATGCTTTAGCCTGCTTTATTATGATTATTTTTGTTGTTTTGGCAATGACCTG

1081 GTTACCTGTTGTTTCTCCCACTAAAACTTTTTAAGGGCAGGAATCACCGCCGTAACTCTA

1141 GCACTTAGCACAGTACTTGGCTTGTAAGAGGTCCTCGATGATGGTTTGTTGAATGAATAC

1201 ATTAAATAATTAACCACTTGAACCCTAAGAAAGAAGCGATTCTATTTCATATTAGGCATT

1261 GTAATGACTTAAGGTAAAGAGCAGTGCTATTAACGGAGTCTAACTGGGAATCCAGCTTGT

1321 TTGGGCTATTTACTAGTTGTGTGGCTGTGGGCAACTTACTTCACCTCTCTGGGCTTAAGT

1381 CATTTTATGTATATCTGAGGTGCTGGCTACCTCTTGGAGTTATTGAGAGGATTATAAGAC

1441 AGTCTATGTGAATCAGCAACCCTTGCATGGCCCCTGGCGGGGAACAGTAATAATAGCCAT

1501 CATCATGTTTACTTACATAGTCCTAATTAGTCTTCAAAACAGCCCTGTAGCAATGGTATG

1561 ATTATTACCATTTTACAGATGAGGAACCTTTGAAGCCTCAGAGAGGCTAACAGACATACC

1621 CTAGGTCATACAGTTATTAAGAGAAGGAGCTCTGTCTCGAACCTAGCTCTCTCTCTCTCG

1681 AGTAATACCAGTTAAAAAATAGGCTACAAATAGGTACTCAAAAAAATGGTAGTGGCTGTT

1741 GTTTTTATTCAGTTGCTGAGGAAAAAATGTTGATTTTTCATCTCTAAACATCAACTTACT

1801 TAATTCTGCCAATTTCTTTTTTTTGAGACAGGGTCTCACTCTGTCACCTAGGATGGAGTG

1861 CAGTGGCACAATCACTGCTCACTGCAGCCTCGACTTCCCGGGCTCGGGTGATTCTCCCCA

1921 GGCTCAGGGGATTCTCCCACTTCAGCCTCCCAAGTAGCTGGGACTACAGGTGCGCACCAC

1981 CATCCCTGGCTAATATTTGTACTTTATTTATTTATTTATTTATTTATTTTTTGAGATGG

2041 AGTTTCGCTCTTGTTGCCCGGGCTGGAGTACAGTGGCATGATCTCGGCTCAGTGCAACCT

2101 CTGCCTCCCGGGTTCAAGCGATTCTCCTACCTCATCCCCCTGAGTAGCTGGGATTACAGG

2161 CGCCTGCCACCATGCCTGGCTAATTTTTTGTATTTTTAATAGAGACGAGGTTTCACCATG

2221 TTGGCCAGGCTACTCTCGAACTCCTGATCTCAGGTGATCCACCCGCCTTGGCCTCCCAAA

2281 GTGCTGGGATTACAGGCGTGAGCCACTGCGCCCGGCCTAATATTTGTATTTTTTGTAGAG

2341 ATGGTGTTTTGCCATGTTGTCCAGGCTGGTCTTGAACTCCTGAGCTCAAGCGATCTGCCC

2401 GCCTCTGCTTCCCAAAGTGCTGGGATTACAGGCATGAGCCACCGTGCCTGGCCTAGGTAG

2461 ACGCTTTTAGCTTTGGGGTGTGATGCCTGCCCCAGTATATAGTGAATTTAATTATTGCTA

2521 GAGCTGGCTGTTTGTTAGTTTTCTTTGAACATAAGATACTCATTGTTTTTAGTTTGCAAA

2581 TCCCTCTTCCTTTTTAAAAAATTTCTTTCCCTTAAATTGTTTGCATGTTAGCAATAACAA

2641 ATGCTTAAATGGTGCTATGTGCTAGATACTCTTCTAAGCCCTGTTATGTATATTAACTAA

2701 TTTTTTAAATTACACAAATCAGAGAGGTTAAGTAACTTGCCCAAGATTACCCAACAATAC

2761 TAGGATTTGAACCTAAGTTTGTCTCACCCCAGATTCTGCTCTTAATCTCTAAACTTTTAA

2821 GTTAGTAGTGACAATAGTAGGTATTTATTGAATACTTAACTATGTTTTAGGCGTTGAAGT

2881 AAATATTTTGCAGGCATTATCTAATGTAAACACCCTAAAGTTACATAACAGGTACCCTTT

2941 AGGTAAATAAACACTAGTATGACCTTGGAGGCACAGATAGTTGAAGTAACTTGCCCAATA

3001 TCACTTACATGAAATTGGCCCTCAAATGTGTCTGATACAACCCATGCTGCTTGTAACTAT

3061 CGTTTTAAACTGCCAGGGTAAACTTGGACACACTTGAGCTAAGAAAAAGCTTTTAGATTT

3121 TTGCAAATTAATGTGAAAGATATGCTTTATGTGGATATAATATCTTCTAAATTTCGGGGA

3181 TGGTAGTCCTAGAAATGTAATCCTGCCCTAGCCGAGCTTACCCTGCCAATAATTTTTTAC

3241 AGAATTGGTAAAACGGAGCACCTTTTTTTTGTCCTTGGCCACACTGTTATCAACAGGGTG

3301 TAGATTGACATCAATCTGTAGGTGTAAACCAGAATTACTCTTTGTGACCACCAGGAAATA

3361 GAGCAGTTCAGTTCAGGGGTTTCTTTCTGTGAATTTAGCACTGTGACCTGCATACTACAA

3421 GTCTACTTTGTTTTCTATCCATTGTTTGTATCTGGGTATTGCAAAAGGTAGGAAAAGGAC

3481 CAACCAGATCAGCAGAGAAGAGTTGCCTTGGAGTTTTCTTTTAGTTTTCTGCAGTTCATT

3541 AGATAGTAACTAGGCCATGTCATTTTACTCCCTTGTAGTGAAGATATGTTGAAGTTGTAC

3601 TGGTATACTCTTCTACCTTTCTGTAATTTTATATTGTGTAGACTTGATAAAATTTATGTG

3661 TCAATCACCACCATTAATATCAATATTGAGCCTCAATTCTTATTTTTCTGCCCAGTGGCT

3721 GCCAAATTACTAACATTTACAATAATTCACTACTACTAAGATAATCTACTAGTTCGATCA

3781 CATACTTCAAATTGTTATGGAACTACTGTCTTCAGCATTGTGCTTCTGATAACTGATAAG

3841 TATAATTTTTTTTTTGTCCAGAGTGAACATGTCTATTCTTCCACTGTACACACTAATAAA

3901 AGGAAAAAATTGTAATATTGGGTAAATTCATGTCCTTACACATGTAGTAGTTATGAGCCCA

3961 TGTCCCTAGAATGAGTAATAATTTATCCCTCCCTTGGTTGAATAGTCAAGAATGCTGATT

4021 TTAATTCTTCTAACAGCTTTATCCCTCAGAAGGGAAGGCAAGCAAGTTATATATGTAGTT

4081 TATTTGTAAGACTGATATGAAATTGGAAGATGAATCTACTATTAGCTTTAATTATTTTTA

4141 CATTTAGGAATATTGCATCAGTAACTCATAATTTTGGTTTTCTGTTATCCTGAGTTAACA

4201 CAAATTATCCAAGGAGATGGCGGATCATCTGCTTTGAGGTGTTTTTTTTTGAGAATTTTA

4261 ATGTATCTGAATATAAAAGGTAAAAATATGCCAACTAGCAATTTCTGCCCATTCCAGAAG

4321 TTTGGAAATATTACTCATTACTAGGAATTAAATAAAATATGGTTTATCTATTGTTATACC

4381 TCTTTTAATTCACATAGCTCATTTTTATCTTTTATTTTTGTTTGTTTTTTTTGAGATGGA

4441 GTCTTGCTCTGTCACCAGGCAGGAGTGCAGTGATGCAATCTCGGCTCACTCTAGCCACCG

4501 ACTCCCTGGTTCAAGCGATTCTCCTGCCTGAGCCTTCTGAGTAGCTGGGATTACAGGCAG

4561 GCACCACCACGCCCAGCTAATTTTTGTAGAGACAGGATTTCACCGTGTTGGCCAGGATGG

4621 TCTCCATCTCCTGACCTCATGATCTGCCTGCTTCGGCCTCCCAAAGTGCTGGGATTACAG

4681 GTGGGAGCCACTACGCCTGGCCCACATAGCTCATTTTTAGACTCACTTCCATTAAGTCTT

4741 GTTTGGACCCACGAACATTGTCTTTTTTTTTTTAAGATGGAGTTTCACTTTTGTTGCCCA

4801 GACTGTAGTGCAATGGTGCAATCTCAGCTCACTGCAATCTCTGCCTCCTGGGTTCTAGCA

4861 ATTCTCCTGCCTCAGCCTCCCGAGTAGCTGGAATTACAGGCGCCCGCCACCACGCCCAGC

4921 TAATTTTTGTGTTTTTAGTAGAGACGGGGTTTCACCATGTTGGGCAGGCCAGGGGTGATC

4981 CGCCCACCTCAGCCTCCCAAAGTGCTGGGATTACAGGTGTGAGCCACCGCATCTGGCCAA

5041 CATGTCTTTTTTTTTTTTTTTCCTTTTTAACCACAAAGAGACTTAAGCAGTCCTTGTCACA

5101 GATGATGAATTGATGTTGCAAGTATTGTCTTAGCTTGGATTAATTTTCTTGCTTACTGTA

5161 ATTTTAGATAATATAGCTTTGTAATTAGAGATTTTATGTGTAAACCACAAAAATGTTTAC

5221 ATGAAGGCCATTATTACAGATGTGACGTGCATAATTATTAGTAATTTGTATGTTTACATG

```
5281 GGTCAGTCTGGCAAAAAATTATGAAGTTTTAAAAATTAAAAAAAATTATAATGCCAGTTT

5341 TACTGGAAAGTAAAATTATTTCAGTAATCGATTATAGCAAAAGTATTGATTTTCATTCCA

5401 GACAAAAGTCAGAATGAAAGGTAATTTCTCAATACTCTTTCAGATTAATAAAAGTACCTG

5461 TAGCGATTTTTATCATTCACAAGTATATCACAAGTAAGTTAGAATTTGAGAACTGTGTTC

5521 TAGATCTCTGAGGAGATGCAGTCAGATTTCTGAACTGTCTCAGCAAATGGTAAGTAACTT

5581 AGAGCTAGTAATTAATAACCTGTCCTTTGATTTCTGATTCAGCCAAGAATGGCCATATTT
                                                                >

5641 GGGAAAGGCAGATCTGGAGAGTAACCACGTTTTCATTCATTTACCACTTCTAGGCCCCTC
     >>>>>>>>>>>>>>>>>>>

5701 CAGAGCTCTCAGATATTTTGGGGTTGAGCCCTTCCCCAAAGCCATACAGGACCTTTTTTT

5761 TGTGATCTGTTCTAGCCATTTTTATGTTGGGTGCTTGTTATGGACTGAGCATTTATGTCC
                                                       <<<<<<<<<

5821 TCCCACACCCCCCCCATACCTTTTTTGAAGTCCTAACCCCCAGTGTGATGGTATTTGGAG
     <<<<<<<<<<<

5881 ACAGGGCCTTTGGAAGGTAATTACAGTTAGAAGAAGTCGGGAGGGTTGGGCCCAGGTCTG

5941 ATTGGATTAGTGCCCTTATATGAAAAGACACCAGGACGGGCGCAGTGGCTCACACCTGTA

6001 ATCCCAGCACTTTGGGAGGCCAAGGTGGGTGGATCACGAGGTCAGGAGTTTGAGACCAGC

6061 CTGGCCAATGTAGTGAAACACCATCTCTACTAAAAATACAAAAATTAGCTGGGTGTGGTA

6121 GCGGGCTCCTGTCATCCAAGCTACTCGGGAGGGTGAGGCATGAGAATCACTTGAACCCGG

6181 GAGTTGGAGGTTGCAGTGAGCCCAGATTGTGCCACTGTACTCCAGCCTGGGTGACAGAGT

6241 GAGACTCTGTCTCAAAAAAGAAAAAAAAAAAAAAAGAGACACCAGAGAGCTTGTTAGAAG

6301 AGGTCATGTGAGCACACAGTTAGAAGACCTTCAAGCCAAAGAAGAGGCCTGAGATTGAAA

6361 CCTACCTTGCAGGTACCTTAATTTTGGACTTCCCAGCCTCCAAAACTGTGAGAAATAAGT

6421 TTCTGTTAAGTCACTCAGTCTGTGGTATTTTGTTATGGCAGCCTGAGCAGGTAGTTGTTC
```

6481 TTTCAGAAGGTGTTGATAATAACCACATGCAACACCAAGTCACAAATAATAAAACAGATG

6541 TAACTTATATTCATACAGAAAGTTGGGCACTGCCATTGCCTTGTTGGTTTACACGGCTGT

6601 GCTAGTTCAGTAGCAGAAAGGTGCTGGTCTCCTTTACTCAGTTTACAATCTAGGCAGTAG

6661 AATGTAATCACTGCTTTAAACTTGATACTGCTTAGGGAGAGAATCATTGGTGCTGGGTAA

6721 CTTTGGGTTCTAGGTTTACTTTTTGTGTATATATAACTGTTTTTGGTAAATCACAAGTTT

6781 CTGGGCTTGTCGAATTAGATTTTGTTACAGATTATGAGCTTTATTATGCTATACAGTTAG

6841 TTGTATGTATATATGCCTTTCCCACTAGATTTTAAGCTTTTTTTTTTTTTTTTTTTTTGT

6901 GACGGAGTCTTGCTCTTGTCGCCCAGGCTGAAGTGGAGTGCAGTGGCACAATCTCGGCTC

6961 ACTGCAGCCTCCACCTCCTAGGTTCAAGCGATTCTCCTGCCTCGGCCTCCCAAGTAACTG

7021 GGACTACAGGCACGTGCCACCACACCCGGCTAATTTTTGTATTTTTTGTAGAGACAGGGT

7081 TTCGCCATGTTGGCTAGGCTGGTCTTGAACTTCTGGCCTCAGGTGATCCACCCGCCTCAG

7141 CCTCCCAAAGTGCTGGGATTTACAGGCATGAGCCACCACGCCCAGCTATAAGCTCTTTAA

7201 GGGTTGTAAATTTATAATCATTCTTTTACTCTCCTGCAAATTCTGTTGCACACTGCCTTA

7261 ATCAAGGTAGATGCTGAATGCATTTTTGTATAATTGAATATGTTGCAATCCCCAACTCTC

7321 TCCAACTGTTCCTGTCAAAGCAGCCACTGGATTGTTAACTAATCCATATTAGATGGGGTT

7381 AATTAATATCAGATGGGACAAGTAAGGGCTAATAAGATTATAGGCCACCAAGTAGATTTC

7441 TGTCTAGCTCTTATAGAGATTGAGTTTATTGGACCTGTTTGATAGGAAGTTTTGGTGTTT

7501 GGGATGATTAAAACTGAAGTTCCTATTTATTGAATTATACCTATTTATATTATTTCATAT

7561 CAGTGGTCCACATGCAAGTGAGGCTTCTGAGACAGAGTTTGAGTTCTCTCTTCAACTACC

7621 ATAACACTTAACCTGTATCTTTTTTTTTTTTTTTTTTTTTAGACGGAGTCTCGCTCTGTC

7681 ACTCAGGCTGGAGTGTAGTGGTATGATCTCGGCTCACTGTAACCTCTGCCTCCTGGATTC

7741 AAGCAGTTCTCCATGTCTCAGCCTCCCTAGTAGCTGGGATTACAGGCCTGTGCCACCATG

7801 CCTGGCTAATTTTTTTTTTTGTATTTTTAGTAGAGACGGGGTTTTACCACGTTGGCCAGGC

7861 TGGTCTCGAACTCTTGACCTCGAGCGATCAACTTGCCTTGGCCTCCCAAAGTGCTGGGAT

7921 TACAGGCATGAGCCACAGCGCCCAGCCGTCTTTTTTTTTAAATAGCAATTTAACACTGTT

7981 CACAGTTACTCATGTACATGTCATGCCATCTATTACACTGTAAGTTCTGTGAGGGTAGCT

8041 GTATCAAATTTATCTAACTCTCTCTAGTATGCATGACATAGTAAGTATTCAATAAATATT

8101 TGCATATTAGTGATAAGGATACAGGTTCTGAATAGTGGGTCCTTACCATTTAAGAATTAG

8161 TATTTGATGGCCGGGCGGGGTGGCTCACGCCTGTAATCCCAGCACTTTGGGAGGCTGAGG

8221 CGGGCGGATCATGAGATCAGGAGATCGAGACCATCCTGGCTAACATGGTGAAATCCCGTC

8281 TTTACAAAAAAAATACAAAAGAATTAACCAAGTGTGGTGGTGGGTGCCTGTAGTCCCAGC

8341 TACTGCTTTGTGAGGCTGAGGCAGGCAGATCACCTGAGGTGGGAAATTCAAGACCAGCCT

8401 GACCAACATGGAGAAACCCCATCTCTACTAAAAATACAAAATTAGCCGGGCGTGGTGGCG

8461 CATGTCTGTAATCCCAGCTACTCGGGAGGCTGAGGCAGGAGAATGGCGTGAACCCGGGAG

8521 GCGGAGCTTGCAGTGAGCCAGGATCGCGCCACTGCACTCCAGCCTGGGCGACAGAGCGAG

8581 ACTCCGTCTCAAAAAAAAAAAAAAAAAAAAAAATTAGTATTTGATATTTGATCATTAAATA

8641 TGAATTAAGAGGACTTAGACTTTTTGTTAAATGTCAAGCTGGGAAAAGTTGTCATTTAAA

8701 TGAATTGCCTCTTATTTAATTTCGTCTGATGATACATTTTGTTTTTATTTTGTAAAAAAT

8761 TATTTTTTTTCTTTTTGGAGACAGGGTCTTGCTCTGTTGCCCAGGCTGGTCACAAACTCC

8821 TGACCTCAAGCAATCCTCCTGCCTTAGCCTCCCAAAATGCTGGGATTACAGGCGTGAGCC

8881 ACCTCGCCCGGCCTTGTATTATGATACATTTTGAACAACTACAAGTAGACTTGGTATAAT

8941 GAACCTGCACGTACCCATTGCCAAGTTCTGACAACTGTCTGTCTATAGCCAATTATGCAT

9001 TTCTTAAATTAGAACCCCCCCAATATACCCAAATATATATATATGTGTGCATATATATAG

9061 TAAGTTGTAACAAAGTTGTGAATTCATACCTGAAGTATCTCAAGTGATGCAAGTTTTATG

9121 AATTTTTGTTTATGCCTTTTGGGAAGAGTTGTATTGACAAATTTTTTATGCTTAAAGTAA

9181 ACCATAAATCAAAAAAATAAAATCTAGGATGCAATAAAACAAAACAACTTCTTGACATAA

9241 GTATGGTATGTAAATCTGTTTTGATTGGAAATCAATTTGTTATATTGCCAGAATTCCTGT

9301 TTTAGAATACATCTCTGCTGATCTGTCTGTATTCTTAGACTGCATATCTGGGATGAACTC

9361 TGGGCAGAATTCACATGGGCTTCCTTTGAAATAAACAAGACTTTTCAAATTCTTAGTCGA

9421 TCTGCAGAACCTGTAGCCAGGCACTGAACCATTTTGATAGATGCAGTAATCGTTGCAAGT

9481 GTATATTTCAAGGAGTTCTGGCTGGGTCCTAGTTTATGCTTGTGGCAGAAGCAGTGAGTA

9541 ACTGGGAGGAAGTTGGTGAGTAAGCTTCAAGGAAGAAGTCATTTTTAGTACTCTGGATCT

9601 TCCTGATTTTAAAGCACTACAAAATGGTGCATTTTCATTCTTGTCAAGTGATAACAGATA

9661 TATTCTGATGAGCCTGAAATGAATATATATTGTATCATTTTTATAATATCTAGCAAGGTT

9721 TGTATTTTCCTAGAACTTGAACTAAATTTCAGTTCATAAAATTTATAAAATACTTAGTTG

9781 TTGTAAAATATTTTTGGAATGTTCACATAGGTGACACACAAATGTCCCATTTTCATTCTT

9841 TCTATAGTAAATATGTTCTGATATGTGAAGGTTTAGCAGATGCATCAGCATTTAATCCTA

9901 GAGGATCTGGCATAATCTTTTCCCCCAAGAATAGAAATTTTTTCTGCTTATGAAAGTAGT

9961 ACATGTTTCTTTAAAAACAAATCAATATTGACTTCTGCCTGCTGTATAGCACTATGCCTC

10021 CACCTGGCCATGACCAGGGGCATGTCCTGGTCCACCTACCTGAAAATGTTTGCAACCAGC

10081 CTCCTGGCCATGTGCACAGGGGCTGAAGTTGTCCCACAGGTATTACGGGCCAACCTGACA

10141 ATACATGAAGTTCCACCAAAGTCTGAGAACTCAGAACTGAGCTTTGGGGACTGAAAGACA

10201 GCACAAACCTCAAATTTCTCAGCACTGGAAACCTCAAAATATAACTGAATTCCATAAATA

10261 AGATTTTAAGTCTTAAATATGTATTTTTAAATGTATTAAAAGTCAAGCTGCTTGTATTTA

10321 AGCACCTAATACAATGCTTAGGTTGTAAAAGGAGATGCTCAATAGGTACTAACTGATATA

10381 TTGAGATTTAATTATGGTTTGACCAATATTTATTGGAAACCGCCAAAGCTTAAATCATCA

10441 GCTTCTTGAATGTGATTTGAAAGGTAATTTAGTATTGAATAGCATGTGAGCTAGAGTATT

10501 TCATTCTTTCTGGTTTATTTCTTCAAATAGACTTTGAATATAATGGTGAATGGGTATTAT

10561 AAATTAACTAATAAAAATGACATTGAAAATGAAAAAATATATATATTAAAGTGTAGAAAG

10621 TGACCAGGCGTGGTGGCTCACACCTGTAATCCAAGCACCTTGGGAGGCTGAGGCAGGAGG

10681 ATCTCTTGATCCCAGGAGTTCAAGACCAGCCTGGGCAACATAGCGAGACTTCGTCTCTAA

10741 AAAAAAAAAAGAGAGAGAAAAAAATTTTTTTATTTAAAAAAAGTGTAGAAAGTGTCAAG

10801 ACCCCACTTCTTACCATTATTTGGTATATTTCTCTATACCCACCCACCCTTCCTCCTTAC

10861 TCCCTCCCTCCCTTCCCAATCTTTTTATCTTTTTGTATTCTGATTTTTTGTTTGTATATT

10921 TTGCTTTAATTTAATGTATCCTTTAAAAATTTCCCATACATTTTATATGTATATATAAAA

10981 ACGCATGCTGCCAAAGATAATTTATAAGAAAGACCATTGAATTTTTTTAAAAGTGATATA

11041 TATTCATTGAAAAAAATTTAGAATATATAGCAAAGCAATAAAGAACTAAATAAAATTGCT

11101 GTAACTCCTCTTTCAAAGATAAGTGCTTTTATGATTTTGTTGTATTTTTTTCTGTATATA

11161 GGTACATATATAGTATTTATAAAGCTGTACTCATAGTACATTTTCACATCACAGGTACCA

11221 TATCAGTGTTATTAAATATTTTGTATGCCAGGGGCTAGACATACCAAGACAACCAATATG

11281 TGGTTCTACTTAAATAATATTAGAGTATCTTTTATGATGACACTTCATGAGTTGACTATA

11341 ATAATCTTAGACTTCTAAGAGTTTGGGTTTTCAAAAGATCACTTAGCTTTTTTGGGTGAT

11401 TTTTCCCCCTTACTGTGAGATGAGAGAGGCTGTTTGGATTTGGGATTGGGGTAGCGGGGA

11461 CAGCAACTTTTCTTTTCTTTTTCTTTTTTATTTTGAGGTAGGGTATTGCTGTGTCACCCA

11521 GGCTGGAGTGCAGTGGTGTGATCTCGGCTCACTGCAACCTCCACCTCCCGGGCTCAGGTG

11581 ATCCTCCTGCTTCAGCCTCCCCAGTAACTGGGACTACAGGCGCGTGCCACATGCCTGGCT

11641 AATTTTGTATTTTTAGTAGAGATGGGGTTTCACCATGTTGGCCAGGCTGGTCTCTAACTC

11701 CTGACCTCAGGTGATACGCCCACCTGGGCCTCCCAAAATACTGGGATTACAGGCATGAGC

11761 CGCTGCATCAGCCAGCAGTTTTTCTTGTGGTTTTTTTTGTTTGTTTTGTTTTGTTTTGTT

11821 TTTGAGATAGGGTCTTACTCTGTTGTCCACGCTGGAGTGCTGTGGTATGATCGTAGCTCA

11881 CTGCAGCCTCAAACTCCTGGGCTCAAGTGATTCCTTCTGCCTCCGCCTCCCGAGTAGCTG

11941 GGACTACAGGTATGCACCACCATACCTGGCAAATTTTTACAAAGTTTTTTGTAGGGACGG

12001 GGTCTTGCTACATTCCCCATGTCGGTCTTGAACTCCTGGCCTCAAGCAACTCTCCTGTCT

12061 CAGCCTCCCAAAGCACTGGGATTACAAGTGTGAGCCACCACACCATGCCAGTTTTTCCTG

12121 TTCAGTGTGATATTTTATCTTGTTAGACTACAGTGTGTTAAAACTTGTTTTACTAAATTT

12181 TCAAACATACTCAAAAGTGGAGAGAATAGTATAATGAATACCCGTATGTTCATCACCCAT

12241 GTTTAGAATATTATTAAATATAAAGATTTTGCTGCGTTTGTCTTAGCTCTTTAAAATTTT

12301 TCTTTTTCTCTTTGTGACCTAAAGGAAATTCCATATCTTATCACTTTACTTCTACATTCT

12361 TGACTAAGATGACTAAGACATATAGTTACATGGTTTTTTGTTTTGTTTTTTGTTTTTTAA

12421 AGACGAAATCTCGCTCTTGTCCCCCAGGCTGGAGTGCAATGGTGCCATCTCAGCTCAGTG

12481 CAACCTCTGCCTTCTGGGTACAAGCGATTCTCCTGCCTCAGCCTCCCAAGTAGCTGGGAT

12541 TACAGGCTCCTGCCACCACGCCTGGCTAATTTTTGTATTTTTAGTAGAGACGGCGGGGGG

12601 AGGTTTCACCATGTTGACAAGGCTGGTCTGGAACTCCTGACCTCAGGTGATCCACCCGCC

12661 TCGGCCTCCCAAAGTGCTGGGATTACAGGCGTGAGCCACCGCGCCCAGCCTGTTTTTTTG

12721 TTTGTTTGTTTTGTTTTTTTTGAGACAGAGTCTTGCTCTGTTTCCCAGGCTGGAGTGAAG

12781 TGGCGCATTCTTGGCTCACTGCAACCTTCACCTCCCAGGTTCAAGTGATTCTCCTGCCTC

12841 AGCCTCCCAAGTAGCTGGGACTACAGGCATGTGTCACCACACCCGGCTAATTTTTTTGTA

12901 TTTTTAGTAGAGACGGGATTTCACCGTGTTGCCCAGGCTGGTCTCGAACTCCTGAGCTCA

12961 GGCAGTCTGCCTGCCTCAGCCTCCCAAAGTGCTGGGATTACACGTGTGAACCAACCCGCC

13021 CGGCCTGTTGTTTTCTTACATAATTCATTATCATACCTACAAAGTTAACAGTTACTAATA

13081 TCATCTTACACCTAAATTTCTCTGATAGACTAAGGTTATTTTTTAACATCTTAATCCAAT

13141 CAAATGTTTGTATCCTGTAATGCTCTCATTGAAACAGCTATATTTCTTTTTCAGATTAGT

13201 GATGATGAACCAGGTTATGACCTTGATTTATTTTGCATACCTAATCATTATGCTGAGGAT

13261 TTGGAAAGGGTGTTTATTCCTCATGGACTAATTATGGACAGGTAAGTAAGATCTTAAAAT

13321 GAGGTTTTTTACTTTTTCTTGTGTTAATTTCAAACATCAGCAGCTGTTCTGAGTACTTGC

13381 TATTTGAACATAAACTAGGCCAACTTATTAAATAACTGATGCTTTCTAAAATCTTCTTTA

13441 TTAAAAATAAAAGAGGAGGGCCTTACTAATTACTTAGTATCAGTTGTGGTATAGTGGGAC

13501 TCTGTAGGGACCAGAACAAAGTAAACATTGAAGGGAGATGGAAGAAGGAACTCTAGCCAG

13561 AGTCTTGCATTTCTCAGTCCTAAACAGGGTAATGGACTGGGGCTGAATCACATGAAGGCA

13621 AGGTCAGATTTTTATTATTATGCACATCTAGCTTGAAAATTTTCTGTTAAGTCAATTACA

13681 GTGAAAAACCTTACCTGGTATTGAATGCTTGCATTGTATGTCTGGCTATTCTGTGTTTTT

13741 ATTTTAAAATTATAATATCAAAATATTTGTGTTATAAAATATTCTAACTATGGAGGCCAT

13801 AAACAAGAAGACTAAAGTTCTCTCCTTTCAGCCTTCTGTACACATTTCTTCTCAAGCACT

13861 GGCTATGCATGTATACTATATGCAAAAGTACATATATACATTTATATTTTAACGTATGAG

13921 TATAGTTTTAAATGTTATTGGACACTTTTAATATTAGTGTGTCTAGAGCTATCTAATATA

13981 TTTTAAAGGTTGCATAGCATTCTGTCTTATGGAGATACCATAACTGATTTAACCAGTCCA

14041 CTATTGATAGACACTATTTTGTTCTTACCGACTGTACTAGAAGAAACATTCTTTTACATG

14101 TTTGGTACTTGTTCAGCTTTATTCAAGTGGAATTTCTGGGTCAAGGGGAAAGAGTTTATT

14161 GAATATTTTGGTATTGCCAAATTTTCCTCTAAGAAGTTGAATCATTTTATACTCCTGATG

14221 TTATATGAGAGTACCTTTCTCTTCACAATTTGTCTCTTTTTTTTTTTTTTTTGAGACAAG

14281 GTCTCTGTTGCCCAGGCTGGGGTGCAGTGCAGCAGAATGATCACAGTTCACTGCAGTCTC

14341 AACCTCCTGGGTTCAAGCGATCCTTCCACCTCAGCCTCCTGAGTAGCTGGGACTATAGGT

14401 GTGCGCCACCACTCCCAGCTAATATTTTTATTTTGTAGAAACAGGGTTCGCCATGTTACC

14461 CAGCCTCCCAAAGTGCTGGGATTACAGGCATGAGCCACTGGCCCAGTTTCTACAGTCTCT

14521 CTTAATATTGTATATTATCCAAGAAATTTCATTTAATCAGAACCTGCCAGTCTGATAGGT

14581 GAAAATGGTATCTTGTTTTTATTTGCATTTAAAAAAAATTATGATAGTGGTATGCTTGGT

14641 TTTTTTGAAGGTATCAAATTTTTTACCTTATGAAACATGAGGGCAAAGGATGTGTTACGT

14701 GGAAGATTTAAAAAAAATTTTAATGCATTTTTTTTGAGACAAGGTCTTGCTCTATTGTCC

14761 AGGCTGGAGTGCAGTGGCACAATCACAGTTCACTCCAGCCTCAACATCCTGCACTAAAGT

14821 GATTTTCCCACCTCACCTCTCAAGTAGCTGGGACTACAGGTACATGCTACCATGCCTGGC

14881 TAATTTTTTTTTTTTTGCAGGCATGGGGTCTCACTATATTGCCCAGGTTGGTGTGGAAGT

14941 TTAATGACTAAGAGGTGTTTGTTATAAAGTTTAATGTATGAAACTTTCTATTAAATTCCT

15001 GATTTTATTTCTGTAGGACTGAACGTCTTGCTCGAGATGTGATGAAGGAGATGGGAGGCC

15061 ATCACATTGTAGCCCTCTGTGTGCTCAAGGGGGGCTATAAATTCTTTGCTGACCTGCTGG

15121 ATTACATCAAAGCACTGAATAGAAATAGTGATAGATCCATTCCTATGACTGTAGATTTTA

15181 TCAGACTGAAGAGCTATTGTGTGAGTATATTTAATATATGATTCTTTTTAGTGGCAACAG

15241 TAGGTTTTCTTATATTTTCTTTGAATCTCTGCAAACCATACTTGCTTTCATTTCACTTGG

15301 TTACAGTGAGATTTTTCTAACATATTCACTAGTACTTTACATCAAAGCCAATACTGTTTT

15361 TTTAAAACTAGTCACCTTGGAGGATATATACTTATTTTACAGGTGTGTGTGGTTTTTTAA

15421 ATAAACTCCTTTTAGGAATTGCTGTTGGGACTTGGGATACTTTTTTCACTATACATACTG

15481 GTGACAGATACCCTCTCTTGAGCTACATCGGTTTGTGGGGAGTCAAAAGTCCTTTGGAGC

15541 TAGGTTTGACAAATAAGGTGGGTTAACACTTGTTTCCTAGAAAGCACATGGAGAGCTAGA

15601 GTATTGGCGAATTGAAGAAATCCCCCTTTTTTTTTAACACACTTAAGAAAGGGGACTGCA

15661 GGTATACTCAAGAGAGTAAGTCGCACCAGAAACCACTTTTGATCCACAGTCTGCCTGTGT

15721 CACACAATTGAAATGCATCACAACATTGACACTGTGGATGAAACAAAATCAGTGTGAATT

15781 TTAGTAGTGAATTTCATTCATAATTTGATCGTGCAAACGTTTGATTTTTATTACTTTAGA

15841 CTATTGTTTCTGATTTTATGTTGGGTTGGTATTTCCTGTGAGTTACTGTTTTCCTTTAAA

15901 ATAGGAATTTTTCATACTCTTCAAAGATTAGAACAAATGTCCAGTTTTTGCTGTTTCATG

15961 AATGAGTCCTGTCCATCTTTGTAGAAACTCGCCTTATGTTCACATTTTTATTGAGAATAA

16021 GACCACTTATCTACATTTAACTATCAACCTCATCCTCTCCATTAATCATCTATTTTAGTG

16081 ACCCAAGTTTTTGACCTTTTCCATGTTTACATCAATCCTGTAGGTGATTGGGCAGCCATT

16141 TAAGTATTATTATAGACATTTTCACTATCCCATTAAAACCCTTTATGCCCATACATCATA

16201 ACACTACTTCCTACCCATAAGCTCCTTTTAACTTGTTAAAGTCTTGCTTGAATTAAAGAC

16261 TTGTTTAAACACAAAATTTAGACTTTTACTCAACAAAAGTGATTGATTGATTGATTGATT

16321 GATTGATGGTTTACAGTAGGACTTCATTCTAGTCATTATAGCTGCTGGCAGTATAACTGG

16381 CCAGCCTTTAATACATTGCTGCTTAGAGTCAAAGCATGTACTTAGAGTTGGTATGATTTA

16441 TCTTTTTGGTCTTCTATAGCCTCCTTCCCCATCCCCATCAGTCTTAATCAGTCTTGTTAC

16501 GTTATGACTAATCTTTGGGGATTTGTGCAGAATGTTATTTTAGATAAGCAAAAACGAGCA

16561 AAATAGGGGAGTTTAACTTTAATATTTTCTTTTAAAAAGCATTTCATGTTATAAGATCAA

16621 TTCTGAGTGGTAGAAATGCTTTGACATTTTATTTCCATTTTCTACTTTTAGTTTTTTTCC

16681 TATTTGTTTAAGATCTTAGAGGATTATTAAGCTGAACTCCTCAACTGATAAAAAGCATGA

16741 CATCTTAAACATAAGCAAAGCATATTTTTAGGTTAATTTTCACATAGAAAACAGTTTATT

16801 TTATGTGAAATTCTATGTAGATATACTATTTTTTTGGTATTTATTGATATGTTTATTTTA

16861 TTTTATTTTATTTTATTTTATTTTATTTTATTTTATTTATTTATTTTTTTTTTTGAGACA

16921 GAGTCTCACTCTGTTGCCCAGGCTGGAGTGCAGTGGCATGATCGTAGCTCACTGCAACCT

16981 CCACCTCCCGGGTTCAAGCAATTCTTCTGTCTCAGCCTCCCGAGTAGCTGGGACTACAGG

17041 TGCCTGCCACTATGCCCGGCTAATTTTTGTGTTTTTAGTAGAGATGGGGTTTCACCTTGT

17101 TGGTCAGGCTGGTCTCGAACCCCTGACCTCAGGTGATCCACCCACCTCAGCCTCCCAAAG

17161 TGCTGGGATTATAGGCATGAGCCACCGTGCCCGGCCGACATGTTAATTTTTTAAAAAAGG

17221 CTTTACTGGGGTATATTTTATATAATATAATAATCACATGTTTTAACTATACAATTCCAA

17281 GCTTTTTAGTATATTTATAGGGCTATGCAAGGAAGATATACTGTTAAACAGTAGAAATTG

17341 AGAAAGCTCTTCTGATAATATCTCTTGATTTGATGATGGCTCATGCCTGTAATCTCAGTG

17401 CTTTGGAAGGCCAAGACAGCAGAATCACTTGAGGCCAGGGGTTCGAGACCAGCCTGGGCA

17461 ACACAGCAATACCCTATCTTTACAAATAATAAAAATATCTGTTGATTTGAAGTAAAGTTT

17521 TTTTTTAAAGACAAGGTCTCATTCTGTCACCCAGGCTGGAATGCAGTAGCAAGATCACAG

17581 CTCACTGTGGCCTTGACCTTCTGGGCTCAAGTGATTCTCCCACTTCGGCCTCCCGAGTAG

17641 CTGGGACTAACAGGTGTGCACCACCATGGCTGGCTAATTTTTTTTTATGTTTGTAGAGAT

17701 TGGGTCTTACTGTGTTGCCCAGGCTGATCCCGAACTCCTGGGCTCAAGCAGTCTTCCTGC

17761 CTCAGCCTCTAAAATTGCTGGGATTACAGGCTTGAGTCACCATGCCCAGCCTGAAGTAGC

17821 ATTTCTACCCTGTTTAATAATTCAGCAGCTTGTCATGTAAGATATTCATATATGCATATA

17881 AAACATTAGGCAGCTTAATTTGGTAAAACTGTAAAATGGAAATTTTAAATTGTTTGCAGC

17941 ATCAATAACATTGATGTCAGTATGATTTTTACATGCTGATCTTGACCAATTTGAAACAGT

18001 GAGTTAAAATCTGGCTGATCCGTACTAATCCTAAAGAAATATTCTATGAACTATTAAATG

18061 TTTCCAGAATATATAAAGAAACATTATGATGTCAACACACCCATCTATTTTTTTTTGGAA

18121 ATAAAAACTCCATTTTTCTTATTAAAGAAAACATGCTTATTAGAAAACATACGGCTGGGT

18181 GCAGTGGCACACATGTAATTCCAGTGCTTTGGGAGATCGAGGTGGGAGAATCACTTGAGG

18241 CCAGGAGTTTGAGACCAGCCTAGACAACATAATGAGACCCCCTCTCTACACAAAAAGAAT

18301 TAGTTGTGCATGGTGGCGTGCACCTGTAGTCCCAGCTACTTGGGAGGCAGAGGCAGGAGC

18361 ATCCCTTGAGCCTAGGAGTTTGAGACTGCAGGAGTTCGAGACTGAGTGGAATGCAGTGGA

18421 ACTGCATTCCAGCCTGAGTGACAGAGGGAGACCCTGTCTTAAAAAAATAAGAAAGAAAAC

18481 ACAACTGCAGAAAATTATAAAGGATTTAAGTCATTCCAAATATCACTGCCACTTTTTATT

18541 TAGAATATTCTAAAGAATTCTCTCTCTGTGTACACACACACATATGCGTACTCTTAATCC

18601 AAGTAGCTTGGTAGGATTTTATTTACCTAGTGCCTAGATGGGAAATTGCCTGGGGATTCC

18661 AAATACCTATTTCATTAAATTAAAGATGTCACTGATTTTAAGACTTAACACTATTTTTCA

18721 TACTGCCAAGAAAGAAAACACTACCAGTTATAAATGTAAATTGCCATCAATTGTAATACA

18781 TCAATTTTAGAGCTATTATTAATAAAATGTGAATGTGCATCTTAGAGCAATGAAATATAG

18841 TACTATATATTTGATGACCTTTTCTGCCCTGTGATATTCAGAAAGTGAAAGTTAAATATG

18901 GGCTGAGCATGGTGGCTCACACCTGTAATCCCAGTACTTTGGGAAGTCAAGACGGGAGGC

18961 TGGCTTGAACCCAGGAGTTCAAGACCAGCCTAGGCAATGTAGCGAGACGCCATCTCAAAA

19021 TATTAAAAATAAGTAAATAAGTAAATAAAAAGAAGGTTAAGTATACAAATGTATTTCCTT

19081 TGTTGTGAATTTATTTCAATTTTATAGTGATTTTTTTTTTTTGAGACGAAGTCTCACTCT

19141 TGTCCCCCAGGCTGGAGTGCGATGGCGTGATCTCAGCTCACTGCAACCTCTGCCTCCCAG

19201 GTTCAAGCTATACTCCTGCCTTGGCCCCCCGAGTAGCTGGGATTACAGGCGCCTGCTACC

19261 ATGCCTGGCTAATTTTTGTATTTTTAGTTGAGATGGGGTTTCACCATGTTGGCCAGGCTG

19321 GTCTAGAACTCTTGACCTCTGGTGATCCACCCGCCTCGGACTCCCAAAATGCTGGGATTA

19381 CAGGCGTGAGCCACCGTGCCTGGCCAGTGGTTTTTTGTTGTTGTTGTTGTTGTTGTTTTG

19441 TTTTTGTTTTTGTTTTTGTTTTGAGACAGGATCTTGCTCTGTCACCCAGGCTGGAGTGCA

19501 GTGGTGCCATCTTGGTTCACTGCAACCTCTGCGTGGGCTCAAGCAATCCTCCCACCTCCC

19561 TTTCCAGAGTAGCGGGGACCACAGGTGTGTGCCACCACACCTGACTAATTTTTGCATTTT

19621 TTTTTGTAGAAACAGGGTTTTGCCATGTTGCCCAGGTTGGTCTGAAACTCCTGAGCTCAA

19681 ACAATCCAACTGCCTTGGCTTCCCTAAGTGAAATTACAGGCATGGGCCACTGTACCCAGT

19741 CTAGTGATTTTTTTATTTTTATTTTTATTTTATTTTATTTTATTTTTTTACCAAAAAAAC

19801 AACAAAGCCTCAGGAGGAAAAGTTGATACACAAGTAAATTTTATTGGAAATGTTTTTTGTG

19861 TGGACCTTAAGCAGAGGGAAAATTAGTCTGCATTATGGTGTATCCAGACTAAATGACTGA

19921 TATTAAAATGAAATTATTCTTAGGATTTGCAATCTTAGAGAAAACTTTTTCATTTTTATT

19981 TTTTTGAGTTACAAATTATCTTCATTTACATTTGAGAACAGTGAGTCACAGAGGGATTAA

20041 GTAACTTACTCAAGATCATACAAGTCTTTGATTTGAACCCAATCTTTTAACTCTGCAGAA

20101 CTCAGAGTCACTCTTATTTGGAAAAACTTTTTAACTGATGTGGATCCTCTAATATGGGCT

20161 TCCTATTATTCATTCTCTATTAGTCAGAAGTTTTGCAAGCAGACAGAATTCATTTTGCCA

20221 ATTACGGGATTTTCCCTCAGTTGCAGTCAAGGTTCATAAAACTATAACTCTTTATCTTTA

20281 ATTAGAAATGTTTTTTTTTTTGAGACAAGGTCTTGCTCTGTTGCCCAGACTGGAATGCAG

20341 TGGCATAGTGGCCCATTGCAGCTTTGAACTCCTGGGCTCAAGGGATCCTCTGCCTCAGCC

20401 TCCCAAGTATCTGAGACTACAAGTGCGTGCCATCACCCATGGCTATTTTAAAAAAAAAAA

20461 AAATTGTAGAGATAGGGTCTTGCTGTGTTGCCCAGGCTGGTCTCAAACTCCTGGTCTCAA

20521 GCAATCCTTCTGCCTTGGTCTCCCAAAGTGCTGAGATTACAGGTGTCAGCCGTTGCACCT

20581 GGCCAAAACGATAACTTAAAATACACACACACACACACACACACAAACACATATGTGTAT

20641 TTGTGTGTGTGTGTGTGTGTGTGTGTCTCAAAAGGTATCAAAAGAGAATAGCTATAACTT

20701 TAGTGTTGATCTTGATAGTGACTTGATTAGGCTCTGTTTAACATCAAAGATGCAAATTAA

20761 TACTTTCTTTGAACATATTAAAAATGCAGAAAATATTGGAGTATTTTATTTTAAATAAAT

20821 TGTATTCTGTATATTTAAGGTATACAACATGATGTTATGGGATACATATAGGTGGTTAAA

20881 AGATTACTGCAGTGAAGCAAATTAACGTATCCCTCAACTCACATAGTTACCCATTTTTTT

20941 TTTGTTTTGGTGGCAAGAGGAGCTTAAAATCTCATTTAGTGTGAATCCCAAATACAGCAC

21001 AATTTTATTACCTATATACTTCATGTTGTACATTATATTTCTAGACTTGTTCATCCTACA

21061 TATCTGCTACTTTGTATCCTCTGAGCTACATCTCCCCATTTTCTCACTTGCCCCCCAAGT

21121 AGTTTCTTAAAGTGTCTCATGTAAGAGGGCAGTAGCTTTCAGCTTAAACTTTTTCTCTGT

21181 ATGTAGTCGATTTCTTTGAGGTATACTTTTCTCTCCAGAATAGTTAGATGTAGGTATACC

21241 ACTTTGATGTTGACACTAGTTTACCTAGAACTTATCTTCTGTAAATCTGTCTCTATTTCC

21301 ATCTCTGTCTCCATCTTTGTCTCTATCTCTATCTGTCTATCTCTATCTATCTATCTATCT

21361 ATCTATCTATCTATCTATCTATCTATCTATCTATCTAAAGCAAATTCATGCCCTTCTCCT

21421 ATTTATTGAATCGAGACCATAGACAGGGGTGAGAGAAAGAATTTGGCAGGAATGGGGATG

21481 TGTATTATCTGTGGCATAAGGAAACTTTACAGAACTAGGTTCAAAAGTATACTTTCTAGT

21541 TCTTTCCCATGGCTTTTCACTTTGATGTAGTCCTTATCAGGTAACTGAGGTTTTATATAA

21601 GTCCCCTGATTCTTAGAACATGAAGGTGTAGTAGTCAAGGTTGGTCCCTTGAAACCACAA

21661 ATTTTGTGAAAAAAAATTAAGAAAATTGAATAATTTCCTCAGCAAATACATATTGATCAT

21721 CTGTTATACAGCCATGAGAAGTGGTTCTGTTGCACACGTTTATTTTATCAGATCCTAATC

21781 CCAAACCAGGCATAAAATGGAAACCATGAAGATAGGATGAAATAACTTCTGAATGTTTGA

21841 ATGTTTGAAAATAGTGTACTTAAAAATACCAGGTGGTTTTTGTTTGTTTTTTGTTTTTTT

21901 CTTTTTTTGAGACAGGGTCTCACTCTGTCACCCAGGCTGGAGTGTAGTGGTGCAATCTCA

21961 TCTCATTGCAGTCTTGACCTCCCAGGCTCAGGTTATCTCCCACCTCAGCCTCCCAAGTAG

22021 CTGGGACTACAGGCACATGCCACCACGCCCAGCTAATTTTTTGTATTTTTTGTAGAGACG

22081 GGGTTTCACCCTGTTGCCCAGGCTGGTCTAGAACTCCTGGGCTTAAGCGATCCTCCCACC

22141 TCAGCCTCCCAAAGTGCTAGGATTACAGGCATGAGCCACCATGCCTGGCAGAAAATACCA

22201 GGTTTTTAAGTATCAGCACTTACTCTTCAATCTTTTCTATTACTATGTTGTGCTAAATGG

22261 TATTTTTTATTTAATTAGAGCAATGCTGTTCAATAGAACTTTCTTTGAGGATGGAAATCT

22321 TTTATGTTTCTGCTATGTGGTACAGAGCCACTAGTGACATGTGGCTTTTGAGCGCTTGAC

22381 ACATCTTGTGCAACACAGGAACTGAATTTTTAAGTAATTTATATTGCCACATGTGGCTAC

22441 CGTATGGGACAGTGTAGTACTAGATGATCTGTAAGGGCTGTGCTTCATCAGTGTCGTTTT

22501 TTAACTGACAAAAACCTTTAGTTTTTTTTTTAGTAATGTGTTTATTTAAAAGAATTCATA

22561 AAATACAAGTAAACAAATTAACTTGTTACCTGAGCATATGTCCTTTCATACTTATTTTTT

22621 CTGCATACATATTTTGGAAAATGGAATATCTGCCCTTTTTTTTTATCTGAGATACAGTCT

22681 ACCTCTAAAAATACATGATTCTAACATTCTCACTTTTGTTGGCATTTGATCAGGGTATAG

22741 AAAAACAGTTAAAAGGACAGAGAATGGTTGAGAGATTATGATATGAAGAGAAAATGTGAT

22801 TGAGTGTGGTAGACTTGGGGCCTGCTTGAATGTTGAGAGAATGACTGTTTTCCGATAAAA

22861 AAAAAAAGTCCATTCTAGGATCCTAAAAGAAGGGTCTGAAGTTCACTGCAGAAAGCAAGC

22921 TACATAGTACTAAGCCACTAAGGGGACATGGAGCCCTTAGTAATTCCTACCTTAGTAATA

22981 GTCTCATCATGCCCTCTTGGGAACCCAGCCTTGTTGATTAGCCTCTCTGCTTTCTCTCCT

23041 TATAGTTCAACCTCCCTGTTTGTTCCAAGCAGTTCTTTTCCTGCCCATTTATTATGCATT

23101 TCTATACAGCTTTCCTCCTCTTTTTCTATACCATGCTGCAGTTCTTATTGCTACCTAGAG

23161 GTTTTCAAAATTCCTAGGGGCGGATAAGTAGGCATAAACAAAGTTCTTCCCTATTATCCT

23221 TCCTATTTTTTCACCTAGACTGAAGAGGTAGACAAAATAGAAATAAAGACATTAAGGGTA

23281 TGTGTTTGTAGTCCCAAAGAGCTTCTCTGGCAATTTTATGTAGTTGACAGTGACGCTCTG

23341 AGTTCAGGACAGATTGGACTCCTTGGCTGAGAGGAGTGAGGAGATAGGACGGTAGAGGAG

23401 AGGGTAGAGCAACTCTGGAGGAAGCTTTCCCCTCACCTTTGCCAGTCCTGTTATCCTAGA

23461 CTTAACCATAATTAAAGATGAGGGAGGCACTCAGTAAAGGGATCTAGTGGGAAGCTTGTT

23521 CCAGACAGCCAAGGAGGGAGGTTCGCGCAGTTCCTTTGGCCACCCAGGTGGGGTAATTGA

23581 TCCATGTATGCCATTCATGTACAATGTAGGCACTTATACCTGTATTCCAATGTAGTGAAC

23641 TATACCATTACTCTTAAATTAATATTCTTTATTAGCTTCCATGGTGGCTATAGGCCAGGC

23701 AAGAGAGTTAAGAAAAAATAAATAGCCAGGTATGGTGACTCAAGCCTGTAATCTCGGCAC

23761 TTTAGGAGGCCGAGGCAGGAGGATCGCTTGAGTCCAGGAGTTCAAGACCAGCCTGAGCAA

23821 AATAGTGAGATCCTGTCTCTATTTTTTAAAAAAGCCTTGGGGCAAACAGGAGTATGGAGG

23881 TTTGGATGCTAATAGAACAGCAGTGTCTTACTGCTTGGAGTTCTCTTGTTTCTTGTCCTA

23941 TCACCGTAGCCTTTGGATCACAGCAATTTTTCCATGACTCCATACTTTTCAGTTCTTGAA

24001 TATTTTTTCCTTTATTCCTCTTGTCTCTGTAAAGACATCAACTGGAGTTGGACTGTAATA

24061 CCAGGTATCTCCAGAAGATGGCACTATTTAACAGATTTTATAAATAATTTGATGTGAGTC

24121 ACTGTCATCTGAAGCTTGTTGCCTTTTCTTTCTTTCTTCTTTCTTTTTTTTCCCCATCAA

24181 TTCTGTATGTTTGAAATGCTGGGTTTTAAGTTAGTTAGAATAAGGGATGTCTGTAATTTC

24241 CCTAAATTGAGAAGTAATATGCAAAGGTTGATATCAGAAGTCATATGCTCACCTTGCAAC

24301 ACCAAATAATACTGGCCCATTTGTGATTTTTGAAAGTAACACTCCATAATAAATGGATGT

24361 ATATATAGAAGCATAACAAAAATAGAAGCACATAAAAGTGAAAAGTCTCATAAACGCCAT

24421 TGTCACTACTCATGTAATTGCTGTTACAAATTTGTTTAAATGTTGAATAAAAATGGTGTC

24481 ATAGGCAACACAGTGTTCCACTACTTGGTGTTTTTAATAGCATTATTCTGTCTCAGTGTG

24541 CTTTGGATTATCAGGTGCTTTTTAATAGTTGCATGGTATTACATTGTGTAGATGAACTTG

24601 ATTAATTTAAATGGTTCCCTGTTAATGGACATGTTGGTTTGTTTTTGTGAACAACTGATA

24661 CAGTGAACATTTATTTTTTAAATAAAAAAAAGAGAGACAGGGTCTTGCTGTGTTTCTCGG

24721 GCTGGCCTTGAACTCCTGGGGTCAAGCGATCGTCTTGCCTCTGCCTCCCTGGGATTACAG

24781 GCATGAAGCCACCGCACCCGGCCCAGTGAACACTCTTGAATGTATCTTTGTATACTTGTC

24841 AGTGTTTTTGTAGCATTGATTCCCAGAAGTGGGATTACTGGATTAAGTGACATGCATGTT

24901 TGCAATTTTAACAGGTATTGCTATGTCATTTTCAAAAGAAGCTATGCCAATTAATACTCT

24961 CACCAACAAGAGTGCTTATTTCCCCTCAGCATATTATCAGGCTTAAGTTTTGCCAGTATG

25021 GGTGGGAGAACAGTAGAATCACATTGTTTTAGTGTTTGTTTCTCAGATAGATATAATTTT

25081 ACACCTTATAACCTTCTCTTCTATAAATTGTCTATTTGTGTTCATTCTCCATTTTCCTAT

25141 GGGTTCTTATTGTTGGAGCCCAATATATAAAAGGGGGTATTTGTTACAGAACCTCTTCAG

25201 TTTTGGTTCATGTCATGCCTGGGTTTTTACCCTTTCTACGGATGTTAAAAAAAATTCTCT

25261 ATTTTCTTCCAGTCCACTTATGGCTTTATTTTTTACATTTAGATTTTAATCCGTCTGGAA

25321 TTTATTTTTGTGTATGCTGTGAGGTAGGGACCATACTTTTATTTTTTCCCAAATGGGTTA

25381 CTAGTTGGCCAAACATCATTTATTGAATAATTCATCTTTTCCCTACTGACTCGAAATACC

25441 ATCTTTATTGTATACTAAATCCTCATATAGTTCTGGGTCTGTTTCTGGGCTCTACTTTGT

25501 TCATTTACTGTGCTGGTACTGCACCGTTGTAATTGCTGTGGCTTTGTGGTATGGTATGGC

25561 TTGCTCTCTGCTAGGGCAAGTCGAAGCTCTTTTGTTCACCTGCTCTTTCACCCAAATTTT

25621 CTGTCCTGAATCCAGCACAGCCAAATTATGGTCATTGTCACCACCAACTACAGTGGGTGT

25681 TGAGCATTTCCCATTGAATCTCCTGTAAGGGTTTTATTGGATTCTGTGATAGCAGTAAAA

25741 TGGGAGCCTAAGAGGTATTCCTTAAAGGACTACTAATCAGACCTGGTTTCCCAGATGATG

25801 CTGAAGATGACGGGGCCTGGGCTAGACTTTTGAGGGACATATCCTTGGGGTTGGGTGTGA

25861 TATAGACCAGCCCTTACAATTTGCTTGACTCATGGGAATCGTACAGGGCCAGAACCAGAC

25921 ACCTGTCATGCTAATAACTTCCCTCACAATTCAGAAATCACTGTGATTGAAGATGGGTGG

25981 CTGTTATAATACTACCCACTTAAAAATGGATGTAACCCATTTTTTAGGACTCTTAAAAAC

26041 ATCAAATCAGTAATGGCCGATTAGGACTTTTTAATTTTTACTAATCTCTACTTGAAAGTT

26101 TTCTAGTCATTCATTTCAGGAAACCTAATTCTTATAATTCATATCATTTAGAATATCATA

26161 ATGCTATGGATATTAGCTAGCTAACTTCTCAAATCTTCTAGTTCTCATTTAATTTGAAGT

26221 TTGTGTGTGTACATAAGGATATACATATACATATGTGTGTGTAGATATATATATATATAG

26281 TTTTTTTTTTTTTTAACTAGAATGACCAGTCAACAGGGGACATAAAAGTAATTGGTGGAG

26341 ATGATCTCTCAACTTTAACTGGAAAGGTATGTATCTTGAAAGGGAAGAAAAAAAAGCACT

26401 TCATACCGAGTCAATTAGTAACAGTGTGCTTTCAATCAATCACTAAGAGATAATTTACAT

26461 AGTATAACTAAATGGGTTATTTAACCCTTGGAAGCAGTCTAGGTTAATTATCGTTCCCTA

26521 GGTCATGTAGTAAAAAGACAGTAGAATCCAACATTAACCTTAAATGTCCATATTGTCAAG

26581 TACTGCTGTCTGCCTCTGTGGGACTCTAATTTGGGATCCTTCAAAAAACATTGATGGGGG

26641 AAAAGATAGCCTTTAAAAAAAAAAAAAAAAACAAACCTATGTGAGTCTATGTGAGGTAGAC

26701 TCACATAGTTTCCTAAAAGATAGCAAAGCAGTATTATGTAGTGGCTGAAAGTGTGAGTTC

26761 CGGAGCCTGACAACTGATTCAAAGCATGGCTTAGTACTTCCTAACTCTGACCTTGGGCAA

26821 GTTACTTAACCTCTCTGTGTCCCATATGTGATTAGGGTGAGGTTGATAATAGCAGCCATA

26881 GAGTTAAGAGGATTAAGTGCTATAATGCAAGTAGAGCTCTTACAACAGTTTCTGGTAAAT

26941 CACTCAATAAATTCAGACATACTATTATTTTAAGAAATCTCAAAGAGTTTTCTTGTACCT

27001 TAAAATTCTCCTAGTGTGAACCATTGGTTTTGGTATATTGTGCTTCCATGTAGTTTAATA

27061 TCAAGATGTTTTTAGATTTCCCTTTTAATTTATTTGTTGACCCATTGGTTGTTCAGGAGC

27121 ATGCTGTTTACCTGAAAATAATGGAGATATTAAGGTATTTGAATATTTATCTTCTAGTAC

27181 ATTGAAAAACTTTTTGAGAGTAACCAATAATAAATGATGGAATGCTACTGCTTTTTTTTT

27241 TTGAAGCTGCCAGTTATTGTTTACTTACACTATGCCAAATATAAAGGCATTAATCTCATA

27301 AAAGTTTCACAACAATCCTGTGAGGGAGACGATATCCCCATTTTACAAATCAGGAAATTA

27361 AGACTTAATAAGGTTAAAAGACTTGCCCAAAGTCACAGAACCAGTAAGTGGTAGAGCTTG

27421 AATTTGAATACAGACCTGACTCTAAAGCTCTTTTCTTTCTTTAGATTTTAGTGTTCATTG

27481 CTTACTTGAATGAGTATCTATAAGAAAACTTTAACATGTAAAACTTCTGTGAAATTATCT

27541 TGTCCCATATCAGGGTCATGTCAAACTAATGTCCTCCTCAGCATCTTTGGAAAACTTCAG

27601 AGGAGAAATGAGCTTTGCCCCTCCTGTTCATTTCATATACCACTGTTAGACCTGTCCTTC

27661 CCTTTCAGCATGCTTTGTCCATATTTAGAAGCTGTTGAAGCCATTACTTGTCTGGTCAGT

27721 TTTTAGTGCTGGAATGGACCTAGCCTTTTAGGCCTTCTGAGATTTAGTTTGATCTCGTCT

27781 TTCCCACCTAATGGCTCTGTTCTACTACATAGATTTGATCTGAAACAGTTCTCTGTTTCT

27841 AAAATAACTTTCTTTTCATGATAGTCACAGTAAAGTACATTTATTATGGAAAAATCAATA

27901 AGTATAACGAGTGAAAGTTATTTCTTGGTGGTAAGATTATGGGATTATTTGAACTTTCTG

27961 TTTCATTGTATTTTATTTATTTATTTATTTTTGTGATGGAGTCTCACTCTGCTGCCCAGG

28021 CTGGAGTGCAGTAGTACGATCTTGGCTCACTGCAACCTCCCCTTCCCAGTTCAAGTGATT

28081 CTCCTGCCTCAGACTCCCAAGTAGCTGGGATTACAGGCGCACGCCACCATGCCTGGCTAA

28141 TTTTTTTATCTTTAGTAGAGACAGGGTTTCACCATGTTGACCAGGCTGATCTCCAACTCC

28201 TGATCTCAGGTATCCACCTGCCTCAGCCTCCCAAAGTACCGGGATTACGGGTGTGAGCCA

28261 CCCTGCCTGGCCTCATTTTGTCTTTTGGGGGTATTTTTGTGTGCAGATATATATGTATAT

28321 AAATATTTTTCCCTCTTTTCCCCAGTTAGTATTTGAGCAGATGAACTTTGGACCCGAATA

28381 CCTGTATTCAAGTCTCTAATACCACTTCTTGGCTATTTTCATTTTATCAAATGGCCTCTT

28441 ATCCTCGTTTTTCTCATTTATTAAGTAGAGATGTAACTACTTGATATAATTCAAAAACTC

28501 AATAATGGCATTCTTTTGTTTTTTAGACTCTAGTGTCTGTACTCGTTGTACCATGCTGGG

28561 ATTCATTTGAACAATTGCATGGCTTTTTTAGTGTATTATTAAATTTGCAGTTTACTTAGA

28621 ATTTACTGGGACCTCATACAAATGGGAAAAAAACATAACTGTGTTACTCATTTGCTGTGT

28681 GCCTTTGGATTGACCCTATTTTTTGTATTCATTTTCTCCCCATGTCCTGAGTTCCACTTT

28741 GAATAAAAAGTAATTTTTTTCCTGCCTGTAAAATAGGCTACCAATAGGCTGCAGTTGTCT

28801 ATAGTAGCTGCTTCACTGAGGAGAGCTCAGCATGAGAGAAATAGTATGAATTGCTTGCCA

28861 CAAGTTATGGGCTAGCCTTACTTCATTCTGTACTTGGACCTGTTTAGGCTTCTAAGAGAT

28921 CTTACCTCCAACAATAAACTGCTTTGAGACATGAAAAGGTGGAAGCTTTACTTGGTTATA

28981 ACTTTACTTTTAATACCTAGAACAGTGAGTCTTCAAACTTGTATTTGCATGCCCAATTTA

29041 TAAAAAGTTTCCTGAGCATTTACCCCTAATATATGCATTTTAAATTATATATGATTTATG

29101 GTAATAATAATATATATGTTACAAAATACATACAAAAATATAGATTAAACAAGGTGAGGT

29161 AAAAAATTTAAAAGTTCTAATCTTTCTTGCAAACCAGTGGATCTTTTGTGCCTTACTCTG

29221 GTAAACACTGTCTTAGAAGAATATATAGAACATTAAAATCTTAATGCTATAGTTATATGA

29281 CAGAGTATGATGAGAGCTACAGATAAACAACACATCATGAATCTTCTTGTGGCAGTGTTT

29341 ATAACCATTATGTGAAATGCTGCCTCATTCTTATAACTAGCATAAGAACAGATAGGACTT

29401 TCTCGATTTTGAGGGGTAATTATTAGATGGTATTTTCTGTTAAGGACTCTTCCAGCTATA

29461 AAATTCTTAAATGTAGAAAGCGAAGTGAGGGTTTATGGTGAGAGGAAGCATTGGTATCAT

29521 GTTTTAGTGTAGTCCAAGAATATGGACACATCCAGAAAATGCAGATCAAGTTTAGCCTAA

29581 TGAGAAAATATATTTTGGAGTCCATATGGTAAATTAAATTATGTGATTTTTGAGTTATTG

29641 TACAAATATAATTCTTAGAATGTTAGAGTCAGGAGACTATAAGAGACCAACTGCTTCAAG

29701 TTTCATTTAACACATGGGAAACTAAGGCCAGAGAAATTTCAAGACTTGCCCAAGATTAGA

29761 CCTCTTGTTAAGTAATGAAAGTGTTTTAAAAACAGGTGGGTCAAATTCTGTTTTTAAAAT

29821 TTCCATTATGATGAAAATTTCAGTATTACAGGCTTCCAAATCCCAGCAGATGGGCCACTT

29881 GTTTAAAGGAGAGTTTGATATAATAAAGCATCTAAAAACAAGAGTTTGGATAATTCCTTA

29941 GGGTTGTTATGATGTGATTTGACTTATAATTGGAAATACCGTTTTATTCATTGTACTGAT

30001 TTTCATTTCTCTTTTTCTTCTAGAATGTCTTGATTGTGGAAGTAAGTTCACATTTACTTT

30061 TAATATAACATTTATGACTTTTCTAACTTAGTATGCACCATCCTAAAGGTAAGCCAGGGA

30121 GAGAAATTCCTCTGCATCAGTTTTAATGGTGGGCTTGTGTTCTAAAGGAGTGAGATTGGT

30181 TTTTTGTAAAGACTACTTAGTAATTTGTTTTTACCAATAATGGAATGGTATACTTCCTAC

30241 CTCTCTTTTTTTAGTTTGAAGTATTTTCTTTCTAAACATAACTCTCTCTCTCTATTTATC

30301 TATATATAATATATACATATATATCTTATATTTTATGTATATATATATATATCTTGCTTA

30361 GATTTTGTCTTATGTAATATTTGGTACATAAAAAATAATATTTATAATTTATAGACTATT

30421 TTCCATGTGTTATTATGTGCTAAAGTATTTTGTATCTTAGCACCGAGAGGCTAAGCAGTT

30481 TCCTAGGGTTACCAGCTAGTAAACTAAGGGAAACCTTTACTTCCTTTAGCTCAGTGGTTC

30541 TCAAAATGTGGTTCCCTAGACCAAAAGTATTAATATCAGACAAGAACCTACCGAATCAAA

30601 ATATCTGTGATGAGGCCCAGCAAGCTATGCTTTAACAAGTTTCCGAGTGATTCTGATGCA

30661 TGCTAAGGTTTAGGATCCCTTGTTTTTACTCATAAGTCACTTTCTCATTAAGGCCTTCCC

30721 TGGCCATCCTATATAAAATCTCATGTTTTCACACCGTCAACTTCGTATTCCTCCTCAATA

30781 CTTTTATTTTCCTGATCACTTATCACTAACAGCCTCTCTCTCTCTCTCTCTCTCTCTCTA

30841 TGTATATATATATATATATCACTTATCACTGTCTAACAGCCTCTCTTTATATATATATAA

30901 TCTATAGATTATATATATATATGCAGCATTGTGCAATCATTATCACGCTCAATTTTAAAACA

30961 TTTTCATTGCCCCACAAAGAAACCCAATCCCCTTAGCCATCACTCCCCATTTCCCCTTCC

31021 CCCAGCACCTAGCAAACTGATCATCTACCTACTTGCTGTCTATAAGATTTGCCTATTCTG

31081 GACATTTTGTATAAATAGAATCATACAATATGTGGCCTTTTGTATCTGGCTTCTCTCACT

31141 TAATGTTTTCAAGGTTCATTCATGTTGTGGAGTATATCTGCACTCATTTCCTTTTTATTG

31201 CCAAATTGTATGGATAGACAGGTGTTCCTCAACTGTGTCCTGATAAACCCATCTGAAGTT

31261 GAAAATATCATAAGTTGAAAATGGATTTACTACTTTGATAAATCTATCCTAAAGTCAGAA

31321 AAATCTCATGTTGAACCATCGTAAGTTGGATACCATCTGAATTACATTTTTGTTATCCAT

31381 TCACTGGTTGACAGACGTTAGGTTGTTTCCACTGTTTGCTCCTTATTTCTCGTACCTGAA

31441 ATGTCCTTATTCCCTCCCTTCTTATCCCATGTTTAAGTCATTTAAGACCCAGCTCAAACG

31501 TCACCTCCACAAAACCTTCCTTGATACCCCTTTCCTCTTCAATTCACTTGGACCTTTTGC

31561 ATTTAATTTTAATTTTTATTTTTTTTAAGACAGAGTCTCACTCTGTCACCAGGCTGGAGT

31621 GCAGTGGTATGATCTCAGCTCACTAACTACTCTGCCTCCCAGGTTCAAGCAATTCTCATG

31681 TCTCAGCCTCCCAAGTAGCTGGGACTACAGGTGTGCGCCACCATGCCTGGCTAATTGTGT

31741 GTGTGTGTGTGTGTATGTATGTATGTATATATGTGTGTGTGTGTATATATATATATACAC

31801 ACACATATATAAATATATATACATATATATATATACACACATATATAAATATATATACAT

31861 ATATATATATACACACACACACACATATATATATATATATAGTTTTTTTTTTTTTTAAGTAG

31921 AGATGGGGTTTTGCCATGTTGGCCAGGCTGGTCTGGCCTCAAGCCATCCTCCCACCTCGG

31981 CCTCGCAAAGTGCTGGTATTATAGGCATGAGCCACTGTGCCTGGCCTGCATTTCATTTTA

32041 ATTATAAAATATTTTGAACTCAGAAAAAAGGGTATGCTGAATACCTACGTACCCACAAAA

32101 GTATTAACATTTTGCCATATTTGCTTCTGATCTTATTTTTTTTGAGAAATTAAAGATCAT

32161 AATACAACTAAAGCCCCATTTCTTTCCCTTCATTCCCAGAAGTATGACAATTATCCTTAA

32221 AGTTGATATATATCATTCCCATGCATGTTTTTTATACTTCCCTAGTACAAGTTAGCTGTA

32281 TCCTCTGCTCAGGGGCTCATCAAGCTGAATCAAGGGACTCATGATCCTCTTCAAAGTTCC

32341 TTCAGGTTGTTGGCAGAATTTAGTTCCTTGTGATTGTAGGACTGAGGGCCCGTTTTCTCA

32401 CTGGCTGCTGGCCAGGGGTTGCTCCCAGATATTTAAAGGCTCATGCCCTAGCCCATGACA

32461 GTCTCACAACATGGCAGCTGACTTCTTCAAAACCAGCAGGAGAATCTTGCTCTAGTCTAC

32521 CACATAACCTAATCACAGGAGCGGCTATCCCGTTATTTTCACAGATCCTGGTCACATTCA

32581 AGGGGAGGGAACCCTTCTGTGTGTGTACACCAGGAGGCAGGAATTTTTTTTTTCTTTTTC

32641 TTTTTTGTTAAAAAGTCTTAAAGTCTTTTATCCCTAAAGGAGGCAGGAATTTTGAGAGCC

32701 ATCAGAATTCTGCCTACCACAGCCCAGAAATCTGCATTTTTCACAAGTCTCCAGCCATGA

32761 TGTTTCTGATGGCTCACACTGCTTTATTCCATTTTTAAAGAGTATTTTTATTGAAAAGCA

32821 TTAGGGTTATGGTTTAAAAAATATTTTCCCTAACAAAGATGGGTTTGTTTAGAGTCCTAC

32881 TTTTGACTAAATAGCTGAGATTCACTTTTATGTAAAGTCATTTTATAGCGTTATTAATTT

32941 GGGTGCCTTTAAAAATAGTATAAAGCATGTTTCTCGAGTGTAGTCTGTTAGCCACCTATA

33001 TTGGAGAGTTGGGAGGAGAGAGTCTCTATCTTGAATTTATGGGAAAAATTCTAAAATACT

33061 TTTTATAATGAAGGACAACATCATAACTCCCTAATAAAATGTGCATGTATATATTCAAAT

33121 TTGCTGTCATTGATCCTGCACCTACAAAATCCAGTCCTGGGGGCTGGCATTCTTACTGCT

33181 TGCTGAGGGCCAGATGATATAGATTCCAGAATATCTCCATGTAGATTTTGGTGAGAATTA

33241 CTGTGCTGAAAAGAATGACAGTATTGCAGTTATACATGGGGGTTTTGGTACTTTATATTG

33301 TGACTCTGAATTTAAAGCTATGCAATGTCTTCTTTTTTGAAAGGATATAATTGACACTGG

33361 CAAAACAATGCAGACTTTGCTTTCCTTGGTCAGGCAGTATAATCCAAAGATGGTCAAGGT

33421 CGCAAGGTATGTATGACATTTTGACACAGAATATTTTCCTCATTTGAAGGGGGATTAAGT

33481 GATTGCTTCTTTTTAAGGATAAATGTTTTCAACTGTCATTTTATCTTCGAAAAGTAATGT

33541 AATCTCATATAAGACTTAAGATATAATCCTTTTAAATAATTTTGTCATGTGTTAATAAAG

33601 CTCATAATTACAGTCACTTCCTTGCCTAATATTAACATTTGGTTTTTCAGCATGCTAATT

33661 ATATCAGTTTGTCCTGAATAGCATGGCAGAGGATTTTGGGCCCCCTTGCAAAATTAAGAA

33721 TAAGGATTCCAAAGCGGGTGAGGAAGTGATAGGAAGGGGTGGGCCCTGAAGATCTGGACC

33781 TCCTGGAATTGAGTGATGAATGCTGCATCTTCTTTGTGTCTGTAGTGAAATTTTATAATG

33841 CCTGCTTCCTTTTTTATTAAGTCGGCCTCACCTCCTCACCTTACCTATGCTGTTTTACTT

33901 TTGCTTTTATAGTTCTACCTGTGTTTATTTCTCATTTTCGTTTCATCTCTCAACAACTCT

33961 GGGGTGGCATTATTATTCCCACTTTTCAGATAAGGTTACTGAGGCATAGGGAATTGTCCA

34021 AAGGTACAGAGCTAGTCCGCTATAGAGATGAGATTTGAACCCAGGGAACCTGGCTCACAG

34081 TTTATGCTTTTGCTACCTTAAGTTTTTAATAGAGTGACATCAAACAAACATTTAAGAATA

34141 TGTTTTTCTTTTCCTTTTATAATTTCATTAAAAACATTAAGTCTCTGATCAGTCTGCAGT

34201 TTTTATGTAGGGGTCAGGTAATGTTCTAACTTCTGCTTTTTCCTAAGTGATTAACAGGTT

34261 TTTATAAGCCCTTTTGAAAAAATCACGGTATCTGTCGAGCATCTTTGAATCAGAGTAAGC

34321 CTTCTAGTGAGTCATATGTCAGCAGTTTGACTGTATGGGCTTTTCTAATATCCAGTTCAA

34381 GTGTTTATCAGTGAGTTTTTCTTTTAAATAGATTTGGGACAGGTACTATGAGAGTATATA

34441 AGTGATACGTTATAGGACACTAACTAGTATCCTATGAAATGGCAAAAACTGCAATCACTT

34501 TTGCACCAACCAAATAGAAACTAATCAGTGCACTTGCTTATTTTTCTACATGCTCTTTAG

34561 GGTTTTAAATGTCAACCTACTGTGGCATAGACTTTAATCCTCTGGGTATTCTTTTGTTGT

34621 TCTTTCCTGGTATATGCTGTGGAATTGAGATAGACTGGTTCGTGAGCGAGAGATTTTGTG

34681 TTGCCACAGGTAGGACATGCTCAAACAATACTTGGGTCATTTCTTGACCCAAGTCATCTA

34741 TTCACCATAGTTTTGTAGCACCGATCTTGCATACATTTCATGTATCTTCTTTGAACCCCA

34801 CGTCAGTGCTGCTTATATGATACTCAGAAATTAAACACTAAGGAATAAGATTTTCAGGTA

34861 GGATTGAGTTTTGGAGGGTCACAAATCTTGTAATGTCTAATATTTCCACTCTCCCTGCTG

34921 AGAATTAGTTTTGGCTTCCTTGGAGGTGATATCGCCTCTGTTGAGTATAAGTGGCCTACT

34981 GTGATCACACCACTGCACTCCAGCCTGGGTGACAGAGTGAGACCCTGTCTCAGAAAAAAA

35041 AAAAAAAAAAAAGAATGCATGGCCTAGATGACTTCTAAGGTTTTTCCCACCCAGTTCCAG

35101 TTTTCATGTTCTAGGCAGAGCAGTAAAGTGAGAAACACATGGACTTGGGAGTTTAGTCTC

35161 GCATTTCACTGCCACTTAATCTGAGCGACTATTCCATATTTAATCTCTCTGAATGTATTT

35221 ACTCATCTTTAAAGGGGAATGATTATTAACATCTTTTTCTCAGGGAAACTATATGAGTCA

35281 AGGAGATAATATATTTGAAAATCTTTTTAACTGCAAAGCGCTGTTTCACTGTTGGTTATA

35341 ATGTGATTGATCTCATTGTAGTGAGCAGCTGCTTAATTGCGTTTTAGAATGTAGGGAAGA

35401 TAGTAATATTTTTCACATTATATATGTAGCTGGTTCTGGAACTGTAAACATACTCCTTTT

35461 TTATGGAGATCTGAGTCACGTACCATAAAATTCACTCTTTTAAAGTTGTACAATCCAGTG

35521 GTTTTTGATATATTCAGAGTTGTGCATCTGCTACCACTATTTCATTTTGGAACCCAAAGA

35581 AACCTTGTACCCATTAGCAGTCATTCTCCCTTCTCCCAGCCCCTGGCAACTACTAATCTA

35641 CTTTCTACAGAAAGTCCGTACAGATTTGTGTATTATGGACATTCCATATAAATGGACTCA

35701 TGCAATATCCTGTCTTCTTTCACTTAGCATAGTGTTTTCAAGGTTCATCTAGGTTGGGGC

35761 ATGTATCAGTACTTCATCCCTTGTTTTGGCTGAATAATATTTCATTGTACAAATATATCA

35821 CATTTTGCTTATCCATCTGTTGGTGAACATTTGAGTTTCTACCTGTTGGCTTTTATGAAT

35881 AATGTTGATTTGAATGTTTGTGTACAAGTATGAATACCTGTTTTCAGGTCTCTTGAGTAT

35941 ATAGTTGCTAGGTCATATAGTAACTCTGTGTTTAACATTTTGAGGAATTGCCCGACTATT

36001 TAACAAGGTATATGTACTGTTTTACACCAGTAACATATGAGGGTTCCAATATCTCCACAT

36061 CCTTGACAACACTTGTTACTGTCCTTTTTATTGTAGCCATCCTAGTGGCTATGATGTGGT

36121 ATCTCATTGTGGTTTTGATTTGTGTTTCTCTGATGCTGATGATGTTGAACATGTTTTCAT

36181 CTGCTTATTGGCCATTTACATATATCTTCTTAAGAACGGTTACCCATTTACAGTATGGAA

36241 AATGCTTCAGATGCAACTCTAGTCATGCCTTAGAGATGGAGCTTTATTAAACATTCAGAT

36301 CTCTAGGCATATGAAGTGCTGAGTTCTCTTGAACTCCTAATACAGATTGCACTGAGTTTA

36361 GTGATACCTTTTCTGGAGCATTCCTGAGTTCAGGTAGGGAGAAGGGTTTTTGCTGTGATT

36421 GGCTTGTTATGTTCTTTCTAAATGGAAATAGAATTGAAGTGTCTCCTCTCTCCATTTATT

36481 GGAAGAGTCATGAGGGACATAATTAGATGATCCCTTGGAGTCTCCGGCTTAGGTCAGTGG

36541 TTATCTACTTAGGCTGCACATTGGAATCACCTGAGAGTTAAAAAACCAGGATAACCTCTG

36601 CCTGTGTCTCATCTCCAGCAATTCTGATGTAATTGGTCAGGGCTGTGGCCCGAGTAGGTG

36661 AGTTCTGGTTTTTTAAAGCTCCCAGGTGATTCTGATGTGCAATCCAGGTTGAGATCACTT

36721 TGGGCCCTTTCCAGCTCTTTAAACATATATATTTATCTAGGAAGGTATGAAAGCATAAGT

36781 TTTCTTGAGACTGCCTTTAACATCTGTAAAGGCTTTCAAAGCAGCTTCTGTAGTTTTTTT

36841 TAAATGGCTGAATATTTTTCAACAGGCAGCATTTGGGTTATAAAATTAGCTTTTGGTAGA

36901 GTTGACTTATACCACCTCCAGCTTTTGTTCCAAAAATAAATACTGGTTCTTTTGGCACAC

36961 TAGTTGTTTTACCCTAAAGTTCCTCTTTGTAAGCCAGTTATTAAAAGTTGTGATGCAGCC

37021 AGGGCGAAGTGGTACACATCTGTAGTCCCAGCTACTCGGAAGGCTGAGGGGGGAGGATCG

37081 CTAGAGCCCAAGAAGTCAAGGCTGCAGTGAACTGTGATTACACCACTGCACTGCAGCCTG

37141 GGCCACAGAGCGAGACTCATCTCTTTAAAAAAAAGAATGTTGTGAGGCCGGGCGCAGTGG

37201 CTCACGCCTGTGATCCCAGCACTTTGGGAGGCCGAGGTGGACGGATCACCTGAGGTTGGG

37261 AGTTCGAGACCAGCCTGACCAACATGGAGAAACCCTGTCTCTACTAAAAAAAATACAAAA

37321 TTAGCCGGGCGTGGTGGCACATGCCTGTAGTCCCAGCTACTCGGCAGGCTGAGGCAGGAG

37381 AATCGCTTGAACCTGGGAGGCAGAGGTTGTGGTGAGTTGGGCGAGCCATTGCACTCCAGC

37441 CTGGGCAACAAGAGCAAAACTCCATCTCAAAAAAAAGAAAAGAAAAGAAAAGAATGTTGT

37501 GGCCAGGCGCGGTGGCTTACGCCTGTAATTTCAGCACTTTGGGAGACCGAGGTGGGCGGA

37561 TCACGAGGTCAGGAGATCAAGACCATCCTGGCTAACACAGTAAAACCCCATCTCTACTAA

37621 ATACAAAAAAAAATTAGCCGGGAGTGCTGGCGGGTGCCTGTAGTCCCAGCTACTCAGGAG

37681 GCTGAGGCGGGAGAATGGCGTGAACCCAGGAGGCAGAGCTTGCAGTGAGCGGAGATCGCG

37741 CCACTGCACTCCAGCCTGGGCAACAGAGCGAGATTCCGTCTAAAAAAAAAAAAAAAGAAT

37801 GTTGTGATAAAAGGTGATGCTCACCTCTCCCACACCCTTTTATAGTTTAGGGATTGTATT

37861 TCCAAGGTTTCTAGACTGAGAGCCCTTTTCATCTTTGCTCATTGACACTCTGTACCCATT

37921 AATCCTCCTTATTAGCTCCCCTTCAATGGACACATGGGTAGTCAGGGTGCAGGTCTCAGA

37981 ACTGTCCTTCAGGTTCCAGGTGATCAACCAAGTGCCTTGTCTGTAGTGTCAACTCATTGC

38041 TGCCCCTTCCTAGTAATCCCCATAATTTAGCTCTCCATTTCATAGTCTTTCCTTGGGTGT

38101 GTTAAAAGTGACCATGGTACACTCAGCACGGATGAAATGAAACAGTGTTTAGAAACGTCA

38161 GTCTTCTCTTTTGTAATGCCCTGTAGTCTCTCTGTATGTTATATGTCACATTTTGTAATT

38221 AACAGCTTGCTGGTGAAAAGGACCCCACGAAGTGTTGGATATAAGCCAGACTGTAAGTGA

38281 ATTACTTTTTTTGTCAATCATTTAACCATCTTTAACCTAAAAGAGTTTTATGTGAAATGG

38341 CTTATAATTGCTTAGAGAATATTTGTAGAGAGGCACATTTGCCAGTATTAGATTTAAAAG

38401 TGATGTTTTCTTTATCTAAATGATGAATTATGATTCTTTTTAGTTGTTGGATTTGAAATT

38461 CCAGACAAGTTTGTTGTAGGATATGCCCTTGACTATAATGAATACTTCAGGGATTTGAAT

38521 GTAAGTAATTGCTTCTTTTTCTCACTCATTTTTCAAAACACGCATAAAAATTTAGGAAAG

38581 AGAATTGTTTTCTCCTTCCAGCACCTCATAATTTGAACAGACTGATGGTTCCCATTAGTC

38641 ACATAAAGCTGTAGTCTAGTACAGACGTCCTTAGAACTGGAACCTGGCCAGGCTAGGGTG

38701 ACACTTCTTGTTGGCTGAAATAGTTGAACAGCTTTAATATACAATAATTGTTGCATTATT

38761 ATTTCAGATGATAAATGTGGTCATAAGTAAGAAATAAATGATCGAGTTTAGTCTTTTAAT

38821 TCACTGTCCTTTGAATACCTGCCTCTTACTCTGGAGGCAGAAGTCCCATGGATGTGTTTA

38881 TGAACATGGTTGAGGAAGATTTAGGAAGACTGCAACAGTACACTACCTAAAGCAGGTTTT

38941 TTACTCCATCTTTTTTTGCCACGTACACTGGCCTCCCACTTTGATATGCTTGAAATTATC

39001 TCCTTGATTTGTCTTTCAAAACTACATATTGAGGCTGGTTGCGGTGGCTCACACCTGTAA

39061 TCCTAGCACTTTGGGAGGCCAAGCCGGACAGATCACTTGAGGTCAGGAGTTCGAGACCAG

39121 CCTGGCAAACATGATGAAACCCCACCTTTACTAAAAATACAAAAATTAGCCAGGCGTAGT

39181 GGTGTGTGCCTGTAACCCAGCTACCTGGGAGGCTGAGGCAGGAGAATCACTGGAACCCGG

39241 GAGGCAGAGGCTACAGTGAGCCAACATCACGCCACTGCACTCCAGCCTGGGTGACAGAGC

39301 AAGACTCTGTCTCAAAACAAAACAAAAAACAAAAAACTACGTATTAAGACAAGAAACAGA

39361 CTGGGCGCGGTGGCTCACGCCTGTAATCCCAGCACTTTGGGAGGCTGAGGCGGGCGGATC

39421 ACAAGGTCAGGAGATCGAGACCATCCTGGCTAACACGGTGAAACCCCGTCTCTACTAAAA

39481 AATAGAAAAAATTAGCTGGGGTGGTGGCGGGCGCCTATAGTCTCAGCTACTCGGGAGGCT

39541 GAGGCAGGAGAATGGCGTGAACCCGGGAGGCAGAGCTTGCAGTGAGCAGAGATCGTGCCA

39601 CTGCACTCCAGTCTGGGTGACAGAGCAAGACTCCGTCTCAAAAAAAAAAACAAAAACAAG

39661 AAACAAATTAAACTAATGTGATAGACTACTGCTTTGTTTTCAAAAGATACACTCCCCAAA

39721 AGTTACTGATCTAAAATACAGTAGTACTATCTCTGTTTAGTAAGAACCCTGACAACTAAT

39781 AGTGTTCTTATATGTAAAATGCTATTCTTGCCTTTCATTTCAGAATATACTTTTTAAATG

39841 TGAATTTCTGGATTTTTTTTTATAGCATGTTTGTGTCATTAGTGAAACTGGAAAAGCAAA

39901 ATACAAAGCCTAAGATGAGAGTTCAAGTTGAGTTTGGAAACATCTGGAGTCCTATTGACA

39961 TCGCCAGTAAAATTATCAATGTTCTAGTTCTGTGGCCATCTGCTTAGTAGAGCTTTTTGC

40021 ATGTATCTTCTAAGAATTTTATCTGTTTTGTACTTTAGAAATGTCAGTTGCTGCATTCCT

40081 AAACTGTTTATTTGCACTATGAGCCTATAGACTATCAGTTCCCTTTGGGCGGATTGTTGT

40141 TTAACTTGTAAATGAAAAAATTCTCTTAAACCACAGCACTATTGAGTGAAACATTGAACT

40201 CATATCTGTAAGAAATAAAGAGAAGATATATTAGTTTTTTAATTGGTATTTTAATTTTTA

40261 TATATGCAGGAAAGAATAGAAGTGATTGAATATTGTTAATTATACCACCGTGTGTTAGAA

40321 AAGTAAGAAGCAGTCAATTTTCACATCAAAGACAGCATCTAAGAAGTTTTGTTCTGTCCT

40381 GGAATTATTTTAGTAGTGTTTCAGTAATGTTGACTGTATTTTCCAACTTGTTCAAATTAT

40441 TACCAGTGAATCTTTGTCAGCAGTTCCCTTTTAAATGCAAATCAATAAATTCCCAAAAAT

40501 TTAA

(primer3_results.cgi release 4.1.0)

Primer pair 1

	Sequence (5'->3')	Length	Tm	GC%	Self complementarity	Self 3' complementarity
**Forward primer**	TGGGAAAGGCAGATCTGGAG	20	59.09	55.00	8.00	2.00
**Reverse primer**	GGGGTGTGGGAGGACATAAA	20	59.00	55.00	3.00	0.00

Products on target templates

>NC_000023.11 Homo sapiens chromosome X, GRCh38.p14 Primary Assembly

```
product length = 192
Features associated with this product:
   hypoxanthine-guanine phosphoribosyltransferase

Forward primer  1          TGGGAAAGGCAGATCTGGAG  20
Template        134465804  ....................  134465823

Reverse primer  1          GGGGTGTGGGAGGACATAAA  20
Template        134465995  ....................  134465976
```

PCR Optimization

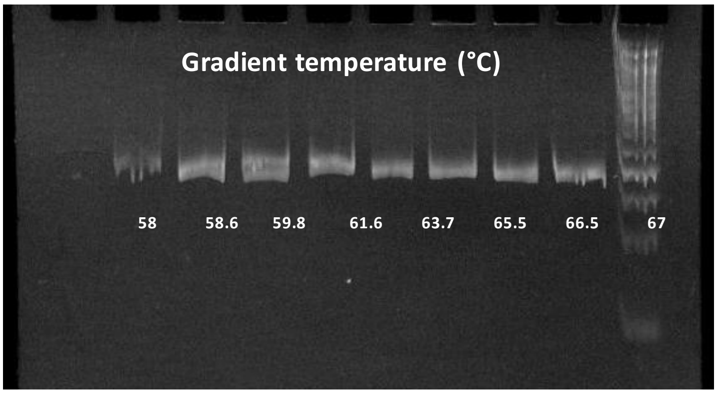

Gradient optimization of a PCR assay for *HPRT1* gene (short fragment). Gel photo showing PCR products formed at different annealing temperatures in a gradient optimization.

Real-time PCR Optimization

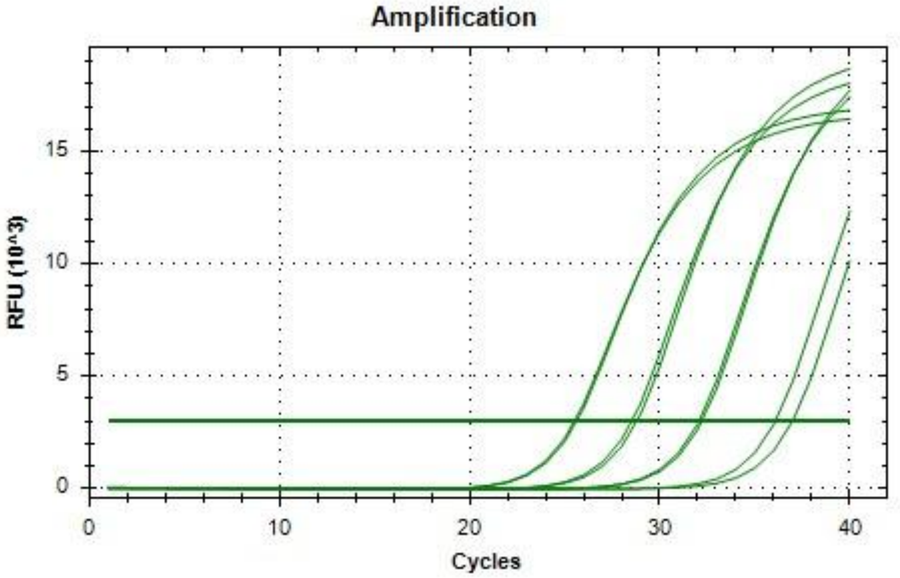

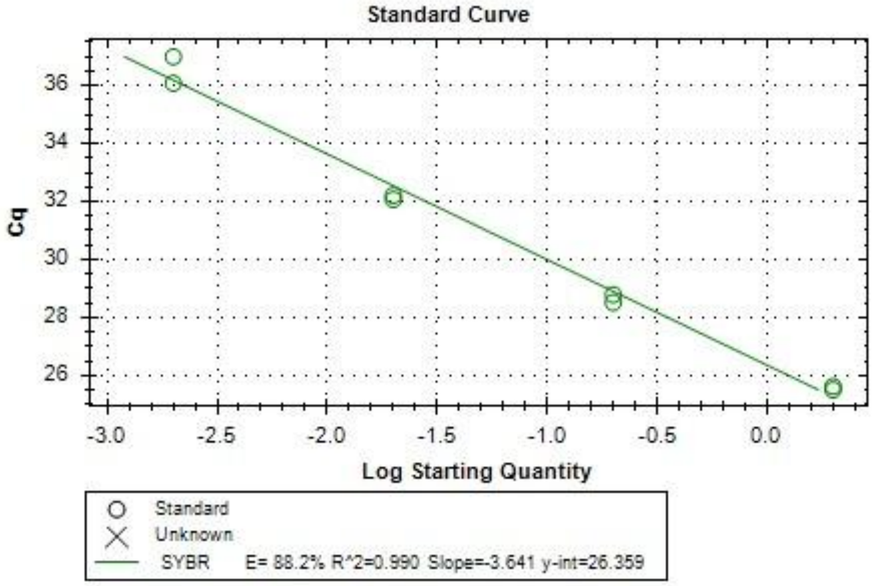

Standard curve with diluted sample: 1/1; 1/10; 1/100; 1/1000.
