# Supplementary material for: Altered Mitochondrial Base Excision Repair and Mitochondrial DNA Instability in Peripheral Leukocytes of Patients with MASLD
Source: Cells. 2026 Aug 5;15(15):1415. doi: 10.3390/cells15151415 (PMC13463988; doi:10.3390/cells15151415)
Supplement: Supplementary file 1 [file cells-15-01415-s001.zip › Supplementary Data S2.pdf]

Supplementary Data S2. List of stable isotope-labelled standard (SIS) peptides used for targeted PRM analysis. The table presents the target proteins and corresponding proteotypic peptide sequences selected for quantification. Please note that several proteins are referred to by alternative names or abbreviations in the main manuscript. The corresponding nomenclature used throughout the manuscript is provided in the table.

| Peptide | Protein | Protein abbreviation used in manuscript |
|---|---|---|
| LDLVLPSGQSFR | sp\|O15527\|OGG1_HUMAN | OGG1 |
| ALCILPGVGTK | sp\|O15527\|OGG1_HUMAN | OGG1 |
| NPEVPFESSAYR | sp\|Q96FI4\|NEIL1_HUMAN | NEIL1 |
| FFNGIGNYLR | sp\|Q96FI4\|NEIL1_HUMAN | NEIL1 |
| EGYSGVGLLSR | sp\|P27695\|APEX1_HUMAN | APEX1 |
| VSYGIGDEEHDQEGR | sp\|P27695\|APEX1_HUMAN | APEX1 |
| NAGFTPQER | sp\|P27695\|APEX1_HUMAN | APEX1 |
| TLSSFFTPR | sp\|P18858\|DNLI1_HUMAN | LIG1 |
| AQIHALEGGEVK | sp\|P18858\|DNLI1_HUMAN | LIG1 |
| DIEQIAEFLEQSVK | sp\|P18858\|DNLI1_HUMAN | LIG1 |
| HVLDALDPNAYEAFK | sp\|P49916\|DNLI3_HUMAN | LIG3 |
| VIQEGLEGLVLK | sp\|P49916\|DNLI3_HUMAN | LIG3 |
| LIADVAPSAIR | sp\|P39748\|FEN1_HUMAN | FEN1 |
| LPIQEFHLSR | sp\|P39748\|FEN1_HUMAN | FEN1 |
| HPDVEVDGFSELR | sp\|P09874\|PARP1_HUMAN | PARP1 |
| TLGDFAAEYAK | sp\|P09874\|PARP1_HUMAN | PARP1 |
| DSPFGLSFVR | sp\|P18887\|XRCC1_HUMAN | XRCC1 |
| YSQVLGLGGR | sp\|P18887\|XRCC1_HUMAN | XRCC1 |
| NHVAVPTHFFK | sp\|Q14249\|NUCG_HUMAN | ENDOG |
| ASGLLFVPNILAR | sp\|Q14249\|NUCG_HUMAN | ENDOG |
| AVLEQFGFPLTGTEAR | sp\|Q9Y2C4\|EXOG_HUMAN | EXOG |
| LSGLVFFPHLDR | sp\|Q9Y2C4\|EXOG_HUMAN | EXOG |
| YGPEGEAVPVAIPEER | sp\|P54098\|DPOG1_HUMAN | POLG |
| IYGAGQPFAER | sp\|P54098\|DPOG1_HUMAN | POLG |
