# Supplementary material for: Altered Mitochondrial Base Excision Repair and Mitochondrial DNA Instability in Peripheral Leukocytes of Patients with MASLD
Source: Cells. 2026 Aug 5;15(15):1415. doi: 10.3390/cells15151415 (PMC13463988; doi:10.3390/cells15151415)
Supplement: Supplementary file 1 [file cells-15-01415-s001.zip › Supplementary Data S3.pdf]

Supplementary Data S3A. Non-significant Spearman's correlations between clinical parameters and molecular markers analyzed in the study. Correlation coefficients and corresponding p-values are reported for all tested associations that did not reach statistical significance ($p \geq 0.05$).

Protein (Normalized peak area ratio)	Clinical parameter	Spearman's r	p-value
APEX1	BMI	0.4618	0.0738
	ALT	-0.00884	0.9759
	AST	-0.1917	0.4757
	HDL	0.09172	0.7365
	LDL	-0.4842	0.0592
	TG	-0.09706	0.7213
	GGT	0.08536	0.7521
	Uric acid	-0.1973	0.4608
	HSI	0.4824	0.0606
	FLI	0.3294	0.2127
PARP1	BMI	0.2559	0.3376
	HbA1c	-0.02817	0.9187
	ALT	0.3918	0.1337
	AST	0.1947	0.4687
	HDL	0.02959	0.9167
	LDL	0.1133	0.6743
	TG	0.2176	0.4168
	GGT	0.06475	0.8112
	Fasting glucose	-0.1794	0.505
	Uric acid	-0.07953	0.7689
	HSI	0.2588	0.3319
	FLI	0.4353	0.0937
FEN1	BMI	0.2176	0.4168
	HbA1c	0.2105	0.431
	ALT	-0.2121	0.4274
	AST	-0.3805	0.1468
	HDL	-0.3669	0.1628
	LDL	-0.1943	0.4678
	TG	0.2559	0.3376
	GGT	-0.2163	0.4181
	Fasting glucose	0.1324	0.6248
	Uric acid	0.2342	0.3798
	HSI	0.2353	0.3791
	FLI	0.4029	0.1229
XRCC1	BMI	0.1941	0.4701
	HbA1c	0.4522	0.08
	ALT	0.01767	0.9497
	AST	-0.351	0.1828
	HDL	-0.2101	0.4337
	LDL	0.08389	0.7566
	TG	0.2559	0.3376
	GGT	-0.4283	0.099
	Fasting glucose	0.2735	0.3043
	Uric acid	-0.2504	0.3469

	HSI	0.3176	0.2302
	FLI	0.3118	0.2393
OGG1	BMI	0.4559	0.0779
	HbA1c	0.215	0.4212
	ALT	0.4595	0.0748
	AST	0.1888	0.4826
	HDL	0.02663	0.9254
	TG	0.2853	0.2832
	GGT	0.06328	0.8157
	Fasting glucose	0.03529	0.8997
	Uric acid	-0.2342	0.3798
	HSI	0.3676	0.1618
	FLI	0.4853	0.0589
LIG3	BMI	-0.2765	0.299
	HbA1c	0.07413	0.7844
	ALT	0.243	0.3616
	AST	0.0708	0.7961
	HDL	-0.4615	0.0739
	LDL	0.08683	0.7482
	TG	0.2765	0.299
	GGT	-0.2281	0.3926
	Fasting glucose	-0.1088	0.6886
	Uric acid	0.1178	0.662
	HSI	-0.1971	0.4632
	FLI	-0.1824	0.4979
LIG1	BMI	0.4235	0.1036
	HbA1c	0.2268	0.3955
	AST	0.3038	0.2521
	HDL	-0.07101	0.7956
	LDL	0.1575	0.5577
	TG	0.4706	0.0679
	GGT	0.05298	0.8455
	Fasting glucose	0.2088	0.4363
	Uric acid	0.1546	0.5647
POLG	BMI	0.4853	0.0589
	HbA1c	0.3084	0.2436
	ALT	-0.05155	0.8498
	AST	-0.2153	0.4219
	HDL	-0.1627	0.5464
	LDL	-0.1884	0.4817
	TG	0.1618	0.5486
	GGT	0.04415	0.8713
	Fasting glucose	0.1971	0.4632
	HSI	0.3088	0.244
	FLI	0.3706	0.1583
ENDOG	BMI	0.2206	0.4103
	HbA1c	0.338	0.1994
	AST	0.41	0.116
	HDL	0.1331	0.6229
	LDL	0.337	0.2008

	TG	0.1824	0.4979
	GGT	-0.1383	0.6069
	Fasting glucose	0.1353	0.617
	Uric acid	-0.1708	0.5242
	HSI	0.1941	0.4701
	FLI	0.1824	0.4979
EXOG	BMI	-0.08235	0.763
	HbA1c	-0.3247	0.2186
	ALT	-0.07364	0.7858
	AST	0.01475	0.9606
	HDL	-0.3521	0.1814
	LDL	-0.3709	0.157
	TG	-0.1382	0.6092
	GGT	-0.01619	0.9542
	Fasting glucose	-0.06471	0.8137
	HSI	-0.07059	0.7967
	FLI	-0.1029	0.7049
NEIL1	BMI	-0.3382	0.2001
	HbA1c	0.01927	0.945
	ALT	0.4698	0.0679
	AST	0.4041	0.1218
	HDL	-0.1598	0.5538
	LDL	0.2119	0.4276
	TG	0.008824	0.9781
	GGT	0.007358	0.9804
	Fasting glucose	-0.1088	0.6886
	Uric acid	0.2784	0.2942
	HSI	-0.3265	0.2169
	FLI	-0.2324	0.3852

Supplementary Data S3B. Correlations among molecular markers assessed in different experimental approaches (gene expression. mitochondrial protein levels. mtDNA copy number. and DNA damage parameters).

Variable 1	Variable 2	Spearman's r	p-value
NEIL1 mRNA level	Copy number of mtDNA	0.02904	0.817
APEX1 mRNA level	Copy number of mtDNA	-0.08015	0.5033
FEN1 mRNA level	Copy number of mtDNA	-0.03182	0.7907
PARP1 mRNA level	Copy number of mtDNA	-0.0382	0.7483
XRCC1 mRNA level	Copy number of mtDNA	0.2329	0.0523
LIG3 mRNA level	Copy number of mtDNA	0.1009	0.4061
OGG1 mRNA level	Copy number of mtDNA	0.2022	0.0884
EXOG mRNA level	Copy number of mtDNA	0.1432	0.2301
ENDOG mRNA level	Copy number of mtDNA	-0.00467	0.9687
NEIL1 mRNA level	mtDNA lesions	-0.1201	0.3151
APEX1 mRNA level	mtDNA lesions	0.1357	0.227
FEN1 mRNA level	mtDNA lesions	0.1475	0.1945
PARP1 mRNA level	mtDNA lesions	0.1859	0.0945
LIG1 mRNA level	mtDNA lesions	-0.1071	0.3738

LIG3 mRNA level	mtDNA lesions	-0.1376	0.2327
EXOG mRNA level	mtDNA lesions	-0.119	0.2931
ENDOG mRNA level	mtDNA lesions	0.06759	0.5463
NEIL1 mRNA level	nDNA lesions	0.03485	0.7778
APEX1 mRNA level	nDNA lesions	-0.03728	0.7475
POLG mRNA level	nDNA lesions	0.001451	0.9901
FEN1 mRNA level	nDNA lesions	-0.02372	0.8399
PARP1 mRNA level	nDNA lesions	0.07589	0.509
XRCC1 mRNA level	nDNA lesions	-0.00657	0.956
LIG1 mRNA level	nDNA lesions	0.09512	0.4439
LIG3 mRNA level	nDNA lesions	-0.102	0.3907
OGG1 mRNA level	nDNA lesions	-0.02555	0.8266
EXOG mRNA level	nDNA lesions	0.01364	0.9069
ENDOG mRNA level	nDNA lesions	-0.0778	0.4984
NEIL1 mRNA level	NEIL1 protein	0.1297	0.6595
APEX1 mRNA level	APEX1 protein	0.1560	0.5944
POLG mRNA level	POLG protein	0.002198	0.9999
FEN1 mRNA level	FEN1 protein	0.07253	0.8083
PARP1 mRNA level	PARP1 protein	-0.1736	0.5526
XRCC1 mRNA level	XRCC1 protein	0.07253	0.8083
LIG1 mRNA level	LIG1 protein	0.2220	0.4448
LIG3 mRNA level	LIG3 protein	-0.09451	0.7500
OGG1 mRNA level	OGG1 protein	0.04615	0.8796
EXOG mRNA level	EXOG protein	-0.3407	0.2335
ENDOG mRNA level	ENDOG protein	0.4488	0.1087
NEIL1 protein	Copy number of mtDNA	-0.3706	0.1583
APEX1 protein	Copy number of mtDNA	-0.02059	0.9432
POLG protein	Copy number of mtDNA	0.2794	0.2937
FEN1 protein	Copy number of mtDNA	-0.1382	0.6092
PARP1 protein	Copy number of mtDNA	0.07353	0.7882
XRCC1 protein	Copy number of mtDNA	-0.02353	0.9345
LIG1 protein	Copy number of mtDNA	-0.2559	0.3376
LIG3 protein	Copy number of mtDNA	-0.3	0.2583
OGG1 protein	Copy number of mtDNA	-0.1824	0.4979
EXOG protein	Copy number of mtDNA	0.1118	0.6806
ENDOG protein	Copy number of mtDNA	-0.00882	0.9781
NEIL1 protein	mtDNA lesions	0.2735	0.3043
APEX1 protein	mtDNA lesions	0.2029	0.4496
POLG protein	mtDNA lesions	-0.01765	0.952
PARP1 protein	mtDNA lesions	0.2794	0.2937
XRCC1 protein	mtDNA lesions	0.1735	0.5193
LIG1 protein	mtDNA lesions	-0.1618	0.5486
LIG3 protein	mtDNA lesions	0.2265	0.3976
OGG1 protein	mtDNA lesions	0.1265	0.6405
EXOG protein	mtDNA lesions	0.1765	0.5122
ENDOG protein	mtDNA lesions	-0.1206	0.6564
NEIL1 protein	nDNA lesions	0.08824	0.7462
APEX1 protein	nDNA lesions	-0.02353	0.9345
POLG protein	nDNA lesions	0.07941	0.7713
FEN1 protein	nDNA lesions	0.3912	0.1351

PARP1 protein	nDNA lesions	0.1971	0.4632
XRCC1 protein	nDNA lesions	0.2059	0.443
LIG1 protein	nDNA lesions	-0.15	0.5786
LIG3 protein	nDNA lesions	0.1353	0.617
OGG1 protein	nDNA lesions	0.1265	0.6405
EXOG protein	nDNA lesions	-0.00588	0.9869
ENDOG protein	nDNA lesions	-0.1382	0.6092
Copy number of mtDNA	nDNA lesions	0.1489	0.1818
mtDNA lesions	nDNA lesions	-0.09456	0.3646
